# Implementation of e–Mental Health Interventions for Informal Caregivers of Adults With Chronic Diseases: Mixed Methods Systematic Review With a Qualitative Comparative Analysis and Thematic Synthesis

**DOI:** 10.2196/41891

**Published:** 2022-11-30

**Authors:** Chelsea Coumoundouros, Erika Mårtensson, Giulia Ferraris, Justine Margaux Zuidberg, Louise von Essen, Robbert Sanderman, Joanne Woodford

**Affiliations:** 1 Healthcare Sciences and e-Health Department of Women’s and Children’s Health Uppsala University Uppsala Sweden; 2 Centre for Gender Research Uppsala University Uppsala Sweden; 3 Department of Health Psychology University Medical Center Groningen University of Groningen Groningen Netherlands; 4 Department of Women’s and Children’s Health Uppsala University Uppsala Sweden; 5 Department of Psychology, Health and Technology University of Twente Enschede Netherlands

**Keywords:** informal caregivers, e–mental health, implementation, chronic diseases, systematic review, thematic synthesis, qualitative comparative analysis, Consolidated Framework for Implementation Research

## Abstract

**Background:**

Informal caregivers commonly experience mental health difficulties related to their caregiving role. e–Mental health interventions provide mental health support in a format that may be more accessible to informal caregivers. However, e–mental health interventions are seldom implemented in real-world practice.

**Objective:**

This mixed methods systematic review aimed to examine factors associated with the effectiveness and implementation of e–mental health interventions for informal caregivers of adults with chronic diseases. To achieve this aim, two approaches were adopted: combinations of implementation and intervention characteristics sufficient for intervention effectiveness were explored using qualitative comparative analysis, and barriers to and facilitators of implementation of e–mental health interventions for informal caregivers were explored using thematic synthesis.

**Methods:**

We identified relevant studies published from January 1, 2007, to July 6, 2022, by systematically searching 6 electronic databases and various secondary search strategies. Included studies reported on the effectiveness or implementation of e–mental health interventions for informal caregivers of adults with cancer, chronic obstructive pulmonary disease, dementia, diabetes, heart disease, or stroke. Randomized controlled trials reporting on caregivers’ mental health outcomes were included in a crisp-set qualitative comparative analysis. We assessed randomized controlled trials for bias using the Risk of Bias 2.0 tool, and we assessed how pragmatic or explanatory their trial design was using the Pragmatic Explanatory Continuum Indicator Summary 2 tool. Studies of any design reporting on implementation were included in a thematic synthesis using the Consolidated Framework for Implementation Research to identify barriers to and facilitators of implementation.

**Results:**

Overall, 53 reports, representing 29 interventions, were included in the review. Most interventions (27/29, 93%) focused on informal cancer or dementia caregivers. In total, 14 reports were included in the qualitative comparative analysis, exploring conditions including the presence of peer or professional support and key persuasive design features. Low consistency and coverage prevented the determination of condition sets sufficient for intervention effectiveness. Overall, 44 reports were included in the thematic synthesis, and 152 barriers and facilitators were identified, with the majority related to the intervention and individual characteristic domains of the Consolidated Framework for Implementation Research. Implementation barriers and facilitators in the inner setting (eg, organizational culture) and outer setting (eg, external policies and resources) domains were largely unexplored.

**Conclusions:**

e–Mental health interventions for informal caregivers tend to be well-designed, with several barriers to and facilitators of implementation identified related to the intervention and individual user characteristics. Future work should focus on exploring the views of stakeholders involved in implementation to determine barriers to and facilitators of implementing e–mental health interventions for informal caregivers, focusing on inner and outer setting barriers and facilitators.

**Trial Registration:**

PROSPERO (International Prospective Register of Systematic Reviews) CRD42020155727; https://www.crd.york.ac.uk/prospero/display_record.php?ID=CRD42020155727

**International Registered Report Identifier (IRRID):**

RR2-10.1136/bmjopen-2019-035406

## Introduction

### Background

Informal caregivers provide essential care and support to individuals with chronic diseases such as cancer and dementia [1,2]. Health care systems rely on informal caregivers to provide a significant portion of the care that individuals with long-term care needs receive [3]. The number of people needed to take on an informal care role is anticipated to increase in the future, as formal care is increasingly shifting to community-based settings [4-6]. Despite the significant societal value of informal care, the informal caregiving role can be a source of burden to informal caregivers [1,2,7,8]. Providing informal care often impacts informal caregivers’ mental health, with informal caregivers reporting worse mental health compared with noncaregivers [7,9]. Meta-analyses have estimated the prevalence of depression among dementia and cancer caregivers to be at 31% [10] and 42% [11], respectively. This is substantially higher than that in the general adult population, in which the prevalence of depression has been estimated to be approximately 8% [12,13].

Despite the prevalence of mental health difficulties among informal caregivers, some evidence suggests that few informal caregivers access mental health support [14,15]. Informal caregivers may not seek mental health support because of common access barriers such as stigma and negative views of mental health interventions [16]. However, informal caregivers can experience additional barriers related to the caregiving role, such as competing demands, limited time available for self-care, lack of awareness of available support, and feelings of guilt for seeking support for themselves instead of focusing on the person they are caring for [16-19]. Delivering mental health interventions using internet-based technologies, referred to as e–mental health interventions [20,21], may improve access to mental health support [22,23]. e–Mental health interventions offer flexible access to mental health support by eliminating the need for informal caregivers to spend time traveling to appointments and can often be used according to the informal caregiver’s schedule [24].

### e–Mental Health Effectiveness and Implementation

e–Mental health interventions can be effective for informal caregivers [25,26]; however, implementation challenges often prevent the integration of e–mental health interventions into practice [27-30]. Implementation challenges span many levels, including factors related to policy (eg, difficulty in navigating regulations), organizations (eg, lack of infrastructure or lack of training), and individual characteristics (eg, negative attitudes and beliefs about e–mental health) [29,31,32]. Evidence suggests that only 3% of evidence-based psychosocial interventions for informal dementia caregivers are translated into practice [30]. Other research suggests that eHealth and e–mental health interventions for dementia caregivers are generally not implementation ready [28].

Currently, the evidence base regarding e–mental health interventions for informal caregivers focuses on intervention effectiveness and efficacy [25,33-38]. Consequently, little is known regarding factors related to the intervention and implementation context that are important to ensure e–mental health interventions for informal caregivers remain effective when implemented. Pragmatic trials may provide more insights into which factors influence intervention effectiveness, given that they are designed to reflect the conditions under which an intervention would be implemented in real-world settings [39]. However, systematic reviews often do not distinguish between evidence derived from pragmatic or explanatory (ie, efficacy) trials [40,41]. Identifying pragmatic trials and examining the conditions under which an intervention was evaluated may provide useful insights into the factors that should be considered when implementing interventions in practice.

Although the literature identifies several barriers to the implementation of e–mental health interventions in real-world settings [28,42-44], few reviews have focused on barriers to and facilitators of implementing eHealth interventions for informal caregivers [27,45-47]. To the best of our knowledge, no review has focused specifically on the implementation of e–mental health interventions for informal caregivers. However, considering contextual factors that may influence intervention effectiveness and implementation is vital for developing interventions optimized for implementation in real-world practice [48]. Implementation is influenced by a variety of contextual factors that many frameworks seek to define [49,50]. The widely used Consolidated Framework for Implementation Research (CFIR) groups factors that influence implementation within five domains: (1) intervention characteristics (eg, the source of the intervention); (2) the outer setting (eg, external policies and incentives); (3) the inner setting (eg, culture of the implementation setting); (4) individual characteristics (eg, knowledge and beliefs about the intervention); and (5) the implementation process (eg, engaging stakeholders in the implementation process) [51]. Consideration of each domain is important for improving our understanding of key factors that influence the implementation of e–mental health interventions for informal caregivers.

### Aims

This review adopted two approaches to examine factors related to the effectiveness and implementation of e–mental health interventions for informal caregivers of adults with chronic diseases: (1) a crisp-set qualitative comparative analysis (QCA) to explore the combinations of intervention and implementation characteristics (eg, provision of support) that are sufficient for intervention effectiveness and (2) a thematic synthesis to identify barriers to and facilitators of the implementation of e–mental health interventions for informal caregivers.

## Methods

### Overview

The protocol for this mixed methods systematic review has been published [52] and reporting follows guidelines defined by PRISMA (Preferred Reporting Items for Systematic Reviews and Meta-Analyses; Multimedia Appendix 1) [53] and PRISMA-S (Preferred Reporting Items for Systematic Reviews and Meta-Analyses-literature search extension; Multimedia Appendix 1) [54], the extension to the PRISMA Statement for Reporting Literature Searches in Systematic Reviews. This review was prospectively registered in PROSPERO (International Prospective Register of Systematic Reviews; CRD42020155727). Full details of the methods can be found in the published protocol [52].

### Selection Criteria

Selection criteria were defined based on the PICOS (population, intervention, comparators, outcomes, study designs) approach [55,56].

#### Population

Informal adult caregivers (aged ≥18 years) provide unpaid care to adults with cancer, chronic obstructive pulmonary disease, dementia, diabetes, heart disease, or stroke. These chronic diseases were selected because they are globally responsible for a significant proportion of disability-adjusted life years due to chronic physical diseases [57] and commonly involve informal care [58,59]. Studies were excluded if they exclusively focused on caregivers who were (1) experiencing severe mental health difficulties, (2) caring for individuals who were non–community dwelling, or (3) caring for an individual near the end of life.

#### Intervention

e–Mental health interventions were defined as those using internet-based technology; for example, web-based platforms or mobile-based apps [21,60]. Interventions designed to target the treatment of common psychological health difficulties (eg, caregiver anxiety, depression, psychological distress, and stress) were included in the review. Any type of mental health treatment, including active or passive psychoeducation [61], was eligible for inclusion. Therapeutic materials had to primarily be delivered using internet-based technology. However, support may have been provided using any delivery mode such as telephone, videoconferencing, or face-to-face contact. Interventions delivering therapeutic materials exclusively using videoconferencing technologies, telephone, or email were excluded.

#### Comparators

For the QCA, which incorporates intervention effectiveness (eg, effect sizes) into the analysis, randomized controlled trials (RCTs) with nonactive controls [62] (eg, usual care, waitlist control, or information on the health condition of the care recipient) were included. For the thematic synthesis exploring barriers to and facilitators of implementation, studies with any design, regardless of the presence or absence of any control type, were included.

#### Outcomes

RCTs included in the QCA reported quantitative data on caregivers’ mental health, specifically anxiety, depression, psychological distress, and stress. Outcome measures were required to have at least acceptable reliability (Cronbach α≥.7; Multimedia Appendix 2 [51,63-72]). Studies included in the thematic synthesis reported barriers to or facilitators of implementation and included either quantitative (eg, questionnaires) or qualitative (eg, interviews or focus groups) data. Barriers to and facilitators of implementation were defined as any aspect related to the CFIR framework [51] (Multimedia Appendix 2) or the implementation outcomes framework, which classifies implementation outcomes related to acceptability, adoption, feasibility, fidelity, reach, appropriateness, implementation cost, and sustainability [73]. Papers describing the development or initial usability of an intervention were included only if it was clear that the intervention was an e–mental health intervention and all other inclusion criteria were met (eg, the paper reported on factors within the CFIR or implementation outcomes, such as acceptability).

#### Study Designs

For the QCA, only RCTs were eligible for inclusion. For thematic synthesis, studies with any type of design (eg, case study or process evaluation) were eligible.

### Search Strategy

Electronic database searches were conducted in CINAHL Plus with Full Text, the Cochrane Library, Embase, PsycINFO, PubMed, and Web of Science databases. Additional searches were conducted in clinical trial registries [74,75] and OpenGrey [76] to identify relevant trial registries and gray literature (eg, research reports).

The literature search was constructed based on terms related to the following PICOS criteria: (1) informal caregivers (eg, caregiver, caregiver, spouse, and partner); (2) chronic diseases targeted in this review (eg, dementia, Alzheimer disease, cancer, stroke, diabetes, or cardiovascular disease); (3) technology (eg, internet, app, or eHealth); (4) mental health (eg, depression, anxiety, or stress); and (5) therapy (eg, psychoeducation or counseling, intervention). A librarian was consulted when constructing the search strategy, and the search strategy was peer reviewed by 2 researchers with experience conducting reviews in similar fields according to the Peer Review of Electronic Search Strategies peer review guidelines [77]. The full search strategy for PubMed is provided in Multimedia Appendix 2, and the search strategies used for all databases and Peer Review of Electronic Search Strategies peer review feedback can be found in the protocol [52].

Studies published between January 1, 2007, and July 6, 2022, were eligible for inclusion. Studies in languages other than English, Dutch, German, or Swedish were excluded.

Forward and backward citation screening was conducted for all included studies, in addition to screening the first 3 pages of the *similar articles* function in PubMed. Experts in the field (n=16) were also contacted for recommendations of studies to be included in the review.

### Study Selection

Database searches were deduplicated using EndNote X9 (Clarivate) [78] and imported into Rayyan [79] to facilitate independent screening of all records by 2 reviewers (CC and EM, GF, or JMZ). Following title and abstract screening, the full texts of all remaining records were checked for eligibility against all elements of the PICOS selection criteria. Conflicts during the screening process were resolved by discussion, and a third reviewer (JW) was consulted as needed. Authors were contacted, at most, twice if more information was needed to determine eligibility. Abstracts, theses, books, commentaries, editorials, and letters-to-the-editor were excluded because of resource limitations. Reviews, trial registries, and protocols were not included; however, (1) references of relevant reviews were screened for studies of interest to the review, (2) published results of relevant trial registries and protocols were sought, and (3) unpublished results from relevant trial registries and protocols were sought from the authors if published results were not yet available. Secondary search strategies (eg, citation screening) were conducted by CC.

Records retrieved from an updated search for papers published between October 2021 and July 2022 (1858 deduplicated records) were screened by only 1 reviewer (CC).

### Data Extraction

Data from the included reports related to (1) study characteristics, (2) participant characteristics, (3) intervention characteristics, and (4) study outcomes were extracted using an Excel spreadsheet (version 2016; Microsoft Corporation). The type of support provided by each intervention was classified based on an adapted version of existing support taxonomies [63,64] (Multimedia Appendix 2). Two independent reviewers (CC and EM, GF, or JMZ) extracted quantitative data to evaluate effectiveness and data related to key intervention characteristics. All other data were extracted by 1 reviewer (CC) and confirmed to be accurate and complete by another reviewer (EM, GF, or JMZ). Data extracted from 6 reports found in the updated search were extracted only by 1 reviewer (CC). The original publication was referred to if differences in extractions were identified, followed by a discussion among reviewers, involving a third reviewer (JW), if needed. Reports with data related to implementation were imported into NVivo (version 1.5.1; QSR International) [80].

### Risk of Bias

The Cochrane Risk of Bias 2.0 tool [81] was used to assess the quality of all included RCTs. The robvis web-based tool was used to visualize the risk-of-bias assessment [82].

Scoring was performed by 2 independent reviewers (CC and EM or Oscar Blomberg), followed by a discussion to reach consensus. As required, a third reviewer (JW) was involved in the discussion.

### Pragmatic Explanatory Continuum Indicator Summary 2 Tool Scoring

RCTs were assessed to determine how pragmatic they were; that is, how well the trial design reflected the real-world setting in which the intervention was likely to be placed. This was evaluated using the Pragmatic Explanatory Continuum Indicator Summary 2 (PRECIS-2) tool [39]. PRECIS-2 evaluated RCTs across 9 criteria. For each criterion, a study receives a score from 1 (very explanatory) to 5 (very pragmatic). Scoring was conducted by 2 independent reviewers (CC and Tasneem Ishrat or JW) followed by discussions to reach a consensus, involving a third reviewer (JW) when needed.

### Data Analysis

#### Overview

A subset of reports included in the review were used for each analytic approach. The QCA included RCTs that evaluated the effectiveness of e–mental health interventions for informal caregivers. The thematic synthesis included reports on factors related to intervention implementation.

#### Qualitative Comparative Analysis

##### Overview

A crisp-set QCA [83] was conducted to explore the sets of conditions that were sufficient for interventions to be effective. QCA is well-suited to the study of complex interventions, such as e–mental health interventions, given that multiple solutions (ie, sets of conditions sufficient for intervention effectiveness) can be identified and contextual factors can be incorporated into the analysis [83,84]. A crisp-set QCA involves dichotomizing intervention outcomes and conditions, producing results that can be interpreted more easily by stakeholders [84]. Effectiveness was measured as the standardized mean difference between the mental health outcomes of the control and treatment groups’ mental health outcomes, calculated using Hedges g and Comprehensive Meta-Analysis (version 3; Biostat Inc). Hedges g was determined for all mental health outcomes of interest in this review; however, only the RCT’s primary mental health outcome was used in the QCA. If RCTs did not identify a primary outcome, the Hedges g for depression scores, the most frequently reported mental health outcome, was used. Hedges g was calculated using data corresponding to the intention-to-treat or modified intention-to-treat analysis, when possible. Conditions explored in the QCA could be related to intervention (eg, provision of professional support) or implementation (eg, provision of training) characteristics.

##### Data Table

To complete the data table for the crisp-set QCA, intervention effectiveness and conditions were dichotomized. To dichotomize effect sizes, interventions were classified as effective (Hedges g ≥0.3) or not effective (Hedges g <0.3). The cutoff used to categorize study effectiveness was based on meta-analyses of e–mental health interventions [85-88]. Conditions were classified as being either present or absent.

##### Truth Table

Truth tables were created to display (1) all potential combinations of conditions used in an analysis, (2) how many interventions had each combination of conditions, and (3) how many interventions with a particular set of conditions were effective. Consistency and coverage scores of 0.75 were used to identify sets of conditions that could be used for Boolean minimization [89,90].

The software fs/QCA (version 3.0; University of California) was used to perform the analysis [91].

#### Thematic Synthesis

##### Overview

The thematic synthesis followed approaches adopted by relevant literature on using qualitative analyses to identify barriers to and facilitators of implementation [92,93]. Data related to implementation were primarily deductively coded using the CFIR. However, data that did not fit within the CFIR were inductively coded. Qualitative and quantitative implementation data were integrated by creating narrative summaries of quantitative data (if not described in the original report) and coding the narrative summary to the relevant CFIR constructs [94]. Initially, approximately 10% (n=4) of the reports included in this analysis were independently coded by 2 reviewers (CC and EM). This was followed by a discussion between 3 reviewers (CC, EM, and JW) to arrive at a shared understanding of the CFIR constructs. The remaining reports were coded by 1 reviewer (CC), with a regular discussion of coding decisions with another reviewer (JW or EM).

After initial deductive coding of data to the constructs within the CFIR, inductive coding was used within each construct to identify a preliminary list of implementation barriers and facilitators. The preliminary list of barriers and facilitators, with all supporting statements from included reports, was shared with a second reviewer (EM) for discussion. A revised set of barriers and facilitators was developed based on these discussions. The revised list of barriers and facilitators was reviewed a final time by a third reviewer (JW), leading to a final set of barriers and facilitators.

##### Professional Stakeholder Involvement

Professionals with expertise in the fields of eHealth and e–mental health (n=4) were consulted for feedback on the results of the thematic synthesis. Professional stakeholders reviewed identified barriers and facilitators and responded to written questions regarding which of the barriers and facilitators they had encountered in practice and what barriers and facilitators they had experienced that were not identified in this review.

### Protocol Changes

After beginning the review process, the following modifications were made to the original protocol [52]:

- Originally, we planned to only include pragmatic RCTs, defined as RCTs with a mean score of ≥3 on the PRECIS-2 tool in the QCA. However, RCTs were not excluded on the basis of their PRECIS-2 scores, as planned, because of the low number of RCTs retrieved. Note that all RCTs included in the review met the planned PRECIS-2 cutoff score for inclusion; therefore, this change did not impact the inclusion or exclusion of any reports.
- After deductive coding of data to the CFIR was completed, only 1 reviewer, rather than 2, independently identified barriers and facilitators with each CFIR construct. However, the barriers and facilitators, with supporting statements initially identified by 1 reviewer (CC), were reviewed in detail by a second reviewer (EM) and were regularly discussed and reviewed by JW.
- The results of the thematic synthesis were only presented to professional stakeholders. Informal caregivers were excluded from the review.

## Results

### Overview

**Figure 1:**
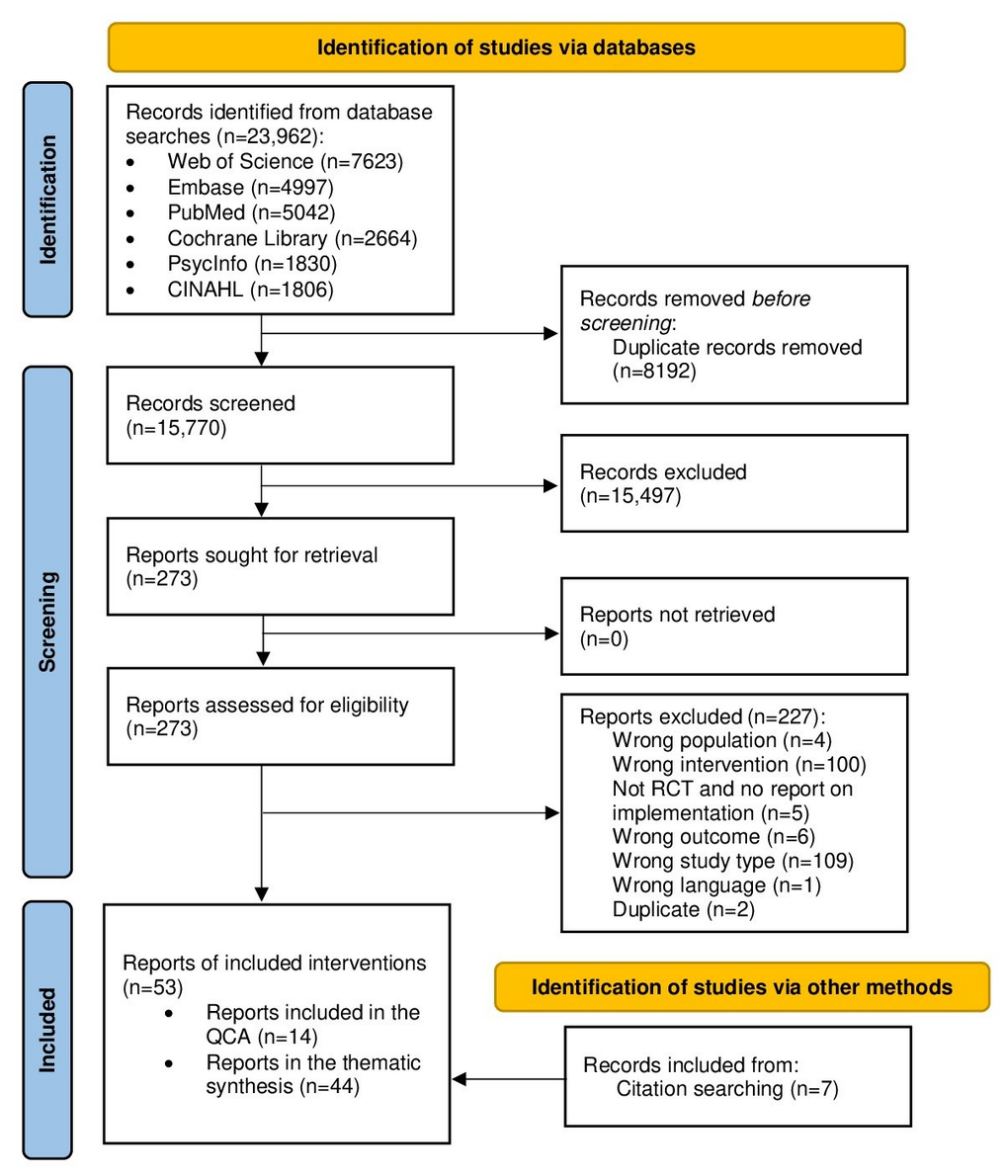
PRISMA (Preferred Reporting Items for Systematic Reviews and Meta-Analyses) flowchart of the study identification process. QCA: qualitative comparative analysis; RCT: randomized controlled trial.

The database searches yielded 23,962 records (Figure 1). After duplicate records were removed (n=8192), titles and abstracts (n=15,770) were screened before full texts (n=273) were retrieved for eligibility screening. Seven included reports were identified using secondary search strategies (eg, backward and forward citation searching). In total, 53 reports (representing 29 interventions) were included in the review (see Multimedia Appendix 3 for a list of excluded studies). The QCA included 14 reports of RCTs, and the thematic synthesis included 44 reports. Five reports were included in both the QCA and thematic synthesis.

### Descriptive Characteristics

#### RCT Characteristics

The characteristics of the 14 reports of RCTs (14 interventions) are presented in Table 1. The RCTs included between 31 and 638 participants, with an overall attrition rate between 11% and 67%. Five RCTs included a follow-up observation point beyond the postintervention follow-up time point [65,95-98], and no RCT included a follow-up beyond 6 months after the intervention was completed. Depression was the most commonly measured mental health outcome (n=12), followed by anxiety (n=8), stress (n=6), and distress (n=1). General mental health was measured in 2 RCTs.

**Table 1:** Descriptive characteristics of randomized controlled trials (n=14).

Caregiver characteristics			Care recipient health condition		Type of control		Follow-up time points, and study attrition rates
**Baruah et al [99], 2021; India**							
	N=151 (I^a^: 74, C^b^: 77) Mean age (years)—I: 46.5 (SD 14.1), C: 42.2 (SD 11.9) Female I: 46% (34/74), C: 47% (36/77) Spouse I: 20% (15/74), C: 21% (16/77) Mean baseline mental health score (CES-D^c^ 10)—I: 10.3 (SD 5.1), C: 11.1 (SD 5.1)	Dementia		Information		Postintervention follow-up: I+C: 64% (96/151), I: 61% (45/74), C: 66% (51/77)	
**Blom et al [100], 2015; Netherlands**							
	N=251 (I: 151; C: 100) Mean age (years)—I: 61.5 (SD 11.9), C: 60.8 (SD 13.1) Female—I: 70% (104/149), C: 69% (66/96) Spouse—I: 60% (89/149), C: 56% (54/96) Mean baseline mental health score (CES-D 20)—I: 17.9 (SD 9.1), C: 16.6 (SD 9.7)	Dementia		Information		Postintervention follow-up: I+C: 30% (76/251), I: 40% (61/151), C: 15% (15/100)	
**Bodschwinna et al [98], 2022; Germany**							
	N=60 (I: 30, C: 30) Mean age (years): I: 47.7 (SD 8.9), C: 47.7 (SD 10.5) Female—I: 68% (23/30), C: 62% (18/29) Spouse—I: 100% (30/30), C: 100% (29/29) Mean baseline mental health score (PHQ-8^d^): I: 8.9 (SD 4.8), C: 8.2 (SD 4.5)	Cancer		WLC^e^		Postintervention follow-up: I+C: 18% (11/60), I: 27% (8/30), C: 10% (3/30) 2-month follow-up: I+C: 30% (18/60), I: 33% (10/30), C: 27% (8/30)	
**Boots et al [101], 2018; Netherlands**							
	N=81 (I: 41, C: 40) Mean age (years): I: 67.8 (SD 10.2) C: 70.2 (SD 10.1) Female—I: 71% (29/41), C: 60% (24/40) Spouse—I: 90% (37/41), C: 93% (37/40) Mean baseline mental health score (CES-D 20)—I: 13.1 (SD 8.7), C: 13.1 (SD 9.0)	Dementia		WLC		Postintervention follow-up: I+C: 16% (13/81), I: 24% (10/41), C: 8% (3/40)	
**Christancho-Lacroix et al [95], 2015; France**							
	N=49 (I: 25, C: 24) Mean age (years)—I: 64.2 (SD 10.3), C: 59.0 (SD 12.4) Female—I: 64% (16/25), C: 67% (16/24) Child—I: 64% (16/25), C: 54% (13/24) Mean baseline mental health score (PSS^f^-14)—I: 24.2 (SD 9.0), C: 24.5 (SD 6.7)	Alzheimer disease		TAU^g^		Postintervention follow-up: I+C: 18% (9/49), I: 20% (5/25), C: 17% (4/24) 3-month follow-up: I+C: 31% (15/49), I: 32% (8/25), C: 29% (7/24)	
**DuBenske et al [65], 2014; United States**							
	N=285^h^ (I: 144, C: 141) Mean age (years)—I: 56.6 (SD 12.9), C: 54.6 (SD 12.2) Female—I: 66% (82/124), C: 70% (86/122) Spouse—I: 73% (91/124), C: 70% (86/122) Mean baseline mental health score (negative mood^i^)—I: 0.88 (SD 0.77), C: 1.1 (SD 0.88)	Lung cancer		TAU and information		Postintervention follow-up^j^: I+C: 40% (115/285), I: 41% (59/144), C: 40% (56/141) 2-month follow-up^k^: I+C: 49% (139/285), I: 48% (69/144), C: 50% (70/141)	
**Fossey et al [102],** **2021; United Kingdom**							
	N=638 (intervention 1^l^: 213, intervention 2^l^: 213, C: 212) Mean age (years)—intervention 1^l^: 60.2 (SD 12.1), intervention 2^l^: 60.2 (SD 12.6), C: 59.2 (SD 12.0) Female—intervention 1^l^: 85% (182/213), intervention 2^l^: 85% (182/213), C: 85% (181/212) Spouse—intervention 1^l^: 43% (91/213), intervention 2^l^: 48% (102/213), C: 40% (85/212) Mean baseline mental health score (GHQ-12^m^): intervention 1^l^: 16.3 (SD 4.1), intervention 2^l^: 16.3 (SD 4.1), C: 16.5 (SD 3.9)	Dementia		Attention		Postintervention follow-up: intervention 1^l^+intervention 2^l^+C: 67% (430/638), intervention 1^l^: 75% (160/213), intervention 2^l^: 53% (112/213), C: 75% (158/212)	
**Gustafson et al [103], 2019; United States**							
	N=31 (I: 16, C: 15) Age (years): 55-64 (I: 3/16, 19%, C: 3/15, 20%); 65-74 (I: 7/16, 44%, C: 9/15, 60); ≥75 (I: 6/16, 38%, C: 3/15, 20%) Female—I: 69% (11/16), C: 53% (13/15) Spouse—I: 94% (15/16), C: 87% (13/15) Mean baseline mental health score (PHQ-8): I: 4.1 (SD 3.5), C: 4.4 (SD 4.3)	Dementia		Information		Postintervention follow-up: I+C: 16% (5/31), I: 13% (2/16), C: 20% (3/15)	
**Hepburn et al [96], 2022; United States**							
	N=261 (I: 96, control 1^n^: 111, control 2^n^: 54) Mean age (years): I: 66.0 (SD 10.9), control 1^n^: 63.8 (SD 11.6), control 2^n^: 63.7 (SD 10.7) Female: I: 75% (72/96), control 1^n^: 66% (73/111), control 2^n^: 72% (39/54) Spouse: I: 72% (69/96), control 1^n^: 61% (68/111), control 2^n^: 65% (35/54) Mean baseline mental health score (CES-D-20): I: 13.1 (SD 10.0), control 1^n^: 12.1 (SD 10.1), control 2^n^: 11.1 (SD 8.3)	Dementia		Attention; WLC		1-month follow-up^o^: I+control 1^n^+control 2^n^: 25% (64/261), I: 26% (25/96), control 1^n^: 25% (28/111), control 2^n^: 20% (11/54) 4-month follow-up^o^: I+control 1^n^+control 2^n^: 23% (61/261), I: 24% (23/96), control 1^n^: 27% (30/111), control 2^n^: 15% (8/54)	
**Kajiyama et al [104],** **2013** **United States**							
	N=150 (I: 75, C: 75) Mean age (years): I: 55.2 (SD 11.3), C: 57.0 (SD 12.5) Female—I: 83% (38/46), C: 86% (49/57) Spouse—I: 56% (26/46), C: 51% (29/57) Mean baseline mental health score (PSS-10)—I: 18.5 (SD 5.2), C: 16.2 (SD 6.9)	Dementia		Information		Postintervention follow-up: I+C: 31% (47/150), I: 39% (29/75), C: 24% (18/75)	
**Köhle et al [105], 2021; Netherlands**							
	N=203 (intervention 1^p^: 67, intervention 2^p^: 70, C: 66) Mean age (years): intervention 1^p^: 57.0 (SD 9.9), intervention 2^p^: 56.4 (SD 11.2), C: 54.2 (SD 11.0) Female: intervention 1^p^: 70% (47/67), intervention 2^p^: 71% (50/70), C: 70% (46/66) Spouse—intervention 1^p^: 100% (67/67), intervention 2^p^: 100% (70/70), C: 100% (66/66) Main baseline mental health score (HADS^q^): intervention 1^p^: 12.5 (SD 0.7), intervention 2^p^: 12.4 (SD 0.7), C: 12.7 (SD 0.7)	Cancer		WLC		Postintervention follow-up: intervention 1^p^+intervention 2^p^+C: 32% (64/203), intervention 1^p^: 28% (19/67), intervention 2^p^: 44% (31/70), C: 21% (14/66)	
**Kubo et al [106], 2019; United States**							
	N=31 (I: 17, C: 14) Mean age (years)—I: 57.1 (SD 17.4), C: 58.2 (SD 18.6) Female—I: 53% (9/17), C: 64% (9/14) Spouse—I: 47% (8/17), C: 79% (11/14) Mean baseline mental health score (HADS-D^r^)—I: 5.1 (SD 3.4), C: 5.6 (SD 2.9)	Cancer		WLC		Postintervention follow-up: I+C: 16% (5/31), I: 24% (4/17), C: 7% (1/14)	
**Pensak et al [107], 2021; United States**							
	N=72 (I: 36, C: 36) Mean age (years)—I: 53.3 (SD 14.7), C: 55.1 (SD 10.9) Female: I: 73% (19/26), C: 77% (23/30) Spouse—I: 77% (20/26), C: 83% (25/30) Main baseline mental health score (HADS-A^s^)—I: 11.2 (SD 2.5), C: 11.6 (SD 3.0)	Cancer		TAU		Postintervention follow-up: I+C: 11% (8/72), I: 14% (5/36), C: 8% (3/36)	
**Smith et al [97], 2012; United States**							
	N=37^t^ (I: 18^t^, C: 19) Mean age (years)—I: 55.3 (SD 6.9), C: 54.9 (SD 12.9) Female—I: 100% (18/18), C: 100% (19/19) Spouse—I: 100% (18/18), C: 100% (19/19) Mean baseline mental health score (CES-D 20)—I: 21.7 (SD 13.2), C: 17.7 (SD 11.7)	Stroke		Information		Postintervention follow-up: I+C: 14%^t^ (5/37), I: 17%^t^ (3/18), C: 11% (2/19) 1 month follow-up: I+C: 14%^t^ (5/37), I: 17%^t^ (3/18), C: 11% (2/19)	

^a^I: intervention arm.

^b^C: control arm.

^c^CES-D: Center for Epidemiological Studies–Depression scale.

^d^PHQ-8: Patient Health Questionnaire depression scale 8 items.

^e^WLC: waitlist control.

^f^PSS: Perceived Stress Scale.

^g^TAU: treatment as usual.

^h^Adjusted to exclude a dropped treatment arm.

^i^Negative mood was based on a modified version of the Short Version Profile of Mood States.

^j^Postintervention was defined as the primary end point measurement specified within the study (6 months).

^k^Caregivers were followed bimonthly for up to 24 months; however, only measurements up to 8 months of follow-up (2 months after the intervention) were analyzed.

^l^In Fossey et al [102], intervention 1 represents the standard intervention and intervention 2 represents intervention with telephone support.

^m^GHQ-12: General Health Questionnaire–12 items.

^n^In Hepburn et al [96], control 1 represents attention control and control 2 represents WLC.

^o^In Hepburn et al [96], 1-month follow-up is referred to as a 3-month follow-up from baseline in the original paper; 4-month follow-up is referred to as a 6-month follow-up from baseline in the original paper.

^p^In Köhle et al [105], intervention 1 represents an intervention with personalized support and intervention 2 represents an intervention with automated support.

^q^HADS: Hospital Anxiety and Depression Scale.

^r^HADS-D: Hospital Anxiety and Depression Scale–Depression subscale.

^s^HADS-A: Hospital Anxiety and Depression Scale–Anxiety subscale.

^t^Adjusted to exclude 1 participant found ineligible after randomization.

#### Intervention Characteristics

The characteristics of the included interventions (n=29) are summarized in Multimedia Appendix 4 [65,95-147]. The interventions were investigated in the United States (n=16) [65,96,97,103,104,106-125], Europe (n=10) [95,98,100-102, 105,126-141], Australia (n=2) [142,143], and India (n=1) [99,144,145]. Most interventions were designed for informal caregivers of people with dementia (n=16) [95,96,99-104,112-117,126-134,136,141,143-147] and cancer (n=11) [65,98,105-111,119-125,135,137-140,142], with 2 interventions focused on informal caregivers of stroke survivors [97,118]. Interventions were commonly based on cognitive behavioral therapy (n=10) [98-100,102,104,107,115,124,​^125^,^134^,^135^,^137^,^141^-^144^], stress and coping theory (n=9) [65,95-97,101,103,109,110,113,114,121-123,126-133], mindfulness (n=7) [106,108,111,116-120], or acceptance and commitment therapy (ACT; n=3) [105,112,136,138-140,146]. Most interventions included support (n=22), providing standardized (n=9) [99,105-108,111,116-118,124,125,​^138^-^140^,^142^,^144^], guided (n=7) [96,97,101,102,113,​^114^,^119^,^126^-^132^,^134^], or minimal (n=5) [98,100,105,135-141,146] support. A novel support type identified was tailored standardized support that involved automated messages tailored based on information provided by participants while using the intervention (n=2) [65,109,110,122]. Six interventions were fully self-administered [95,103,104,112,115,120,133].

### Qualitative Comparative Analysis

In total, 14 reports of RCTs were included in the crisp-set QCA. On the basis of the Hedges g and the cutoff set to classify interventions as effective or not effective, 5 RCTs were classified as effective (Multimedia Appendix 4). The conditions (intervention and implementation characteristics) explored in the QCA included the presence of peer support, professional support, and a selection of persuasive design elements [148] (reminders and tunneling, ie, controlled module order). None of the explored combinations of 2 or 3 conditions resulted in a consistency and coverage above 0.75 (Multimedia Appendix 4); therefore, the analysis could not proceed.

### PRECIS-2 Scores

**Figure 2:**
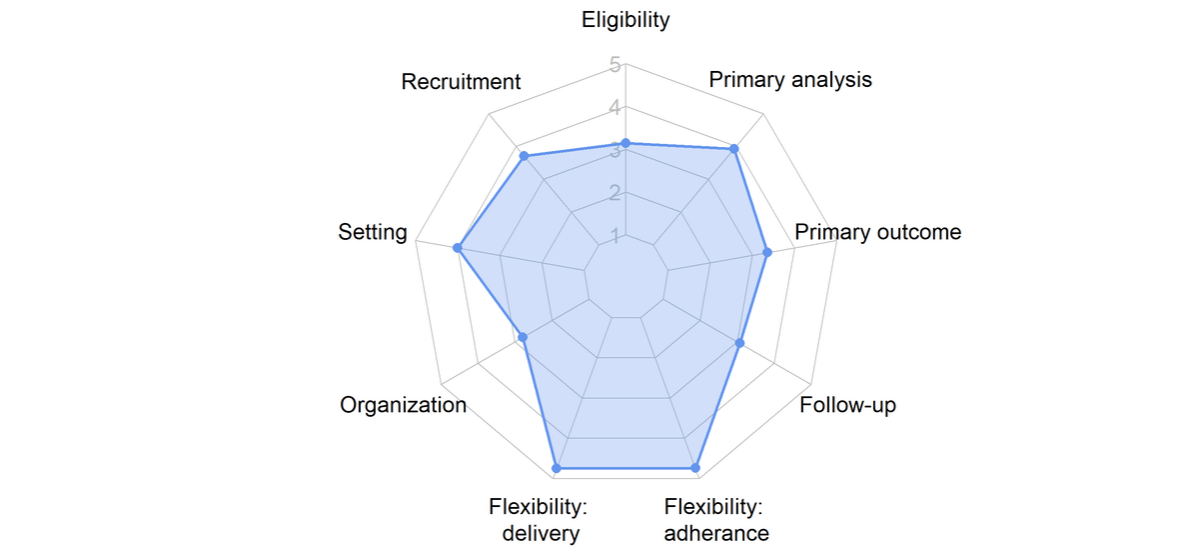
Mean Pragmatic Explanatory Continuum Indicator Summary 2 (PRECIS-2) scores from included randomized controlled trials (n=14). Trials were scored between 1 (very explanatory) and 5 (very pragmatic) for each domain of PRECIS-2.

The 14 RCTs included in the study were scored according to the PRECIS-2 tool to examine how pragmatic each trial was. All RCTs had an average PRECIS-2 score of at least 3, meaning each had at least a mixture of more pragmatic and more explanatory design choices. Domains of the PRECIS-2 tool, which were the most pragmatic across all RCTs, were flexibility of intervention delivery and flexibility of measures taken to monitor and increase adherence (Figure 2). The most explanatory domains were the eligibility of participants, organization of the intervention, and follow-up procedures (Figure 2). PRECIS-2 scoring for individual RCTs can be found in Multimedia Appendix 4.

### Risk of Bias

As assessed using the Cochrane Risk of Bias 2.0 tool [149], 13 RCTs were found to have an overall high risk of bias [65,95-97,99,101-107], with 1 RCT [100] being rated as having some concerns (Multimedia Appendix 4). Domain 4, which assesses bias in the measurement of the outcome, was the domain that largely contributed to the overall high risk of bias in most reports. Bias related to the randomization process (domain 1) and deviation from the intended intervention (domain 2) was most frequently scored as having a low risk of bias.

### Thematic Synthesis

In total, 44 reports (representing 27 interventions) were included in the thematic synthesis (Table 2). The most frequently reported domains were *innovation characteristics* and *individual characteristics*. Barriers to and facilitators of the implementation of e–mental health interventions for informal caregivers are reported in Multimedia Appendix 5 [95,98,104,105,108-147] with relevant example quotations. Within the thematic synthesis, barriers and facilitators were attributed to stakeholders and informal caregivers. The term *stakeholder* is used to describe any professional group, for example, health care professionals and staff, involved in implementation.

**Table 2:** Summary of identified barriers and facilitators.

CFIR^a^ construct				Reports that identified barriers		Reports that identified facilitators
**Domain 1: innovation characteristics**						
	Innovation source		No data		[129,130]	
	Evidence strength and quality		[128-130]		[114,125,128-130]	
	Relative advantage		[95,114,121,123,126,128,140,142,146]		[114,117,120,126,127,130,132,136,139,142]	
	Adaptability		[109,126,127,136,138]		[108,113,120,125-127,131,132,134,136,138-140]	
	Trialability		No data		[130]	
	Complexity		[113,114,117,125-128,132,133]		No data	
	Design quality and packaging		[95,98,108,110,111,114,117-120, 123-140,142-146]		[95,98,104,105,108-111,113-117,119, 120,123-128,130-146]	
	Costs		[128,129,147]		[130,143]	
**Domain 2: outer setting**						
	Needs and resources of those served by the organization		[128,129]		[128,130]	
	Cosmopolitanism		[129]		[128-130,143]	
	Peer pressure		No data		[129]	
	External policy and incentives		No data		[128,130]	
**Domain 3: inner setting**						
	Structural characteristics		No data		No data	
	Networks and communications		[128,130]		[128]	
	Culture		No data		No data	
	**Implementation climate**		No data		[144]	
		Tension for change	No data		No data	
		Compatibility	[122,123,127,128,130]		[122,127-129,143,146]	
		Relative priority	[127,128]		No data	
		Organizational incentives and rewards	[128]		No data	
		Goals and feedback	[128]		[128]	
		Learning climate	No data		[146]	
	**Readiness for implementation**		[127,129]		No data	
		Leadership engagement	[127-129]		No data	
		Available resources	[127-129]		No data	
		Access to knowledge and information	[122,123,127,146]		[146]	
**Domain 4: individual characteristics**						
	Knowledge and beliefs about the innovation		[95,98,113,114,117,120-123,126-130, 133,138-140,142,146]		[95,98,105,108,109,112-120, 123-133,135-140,143,145,146]	
	Self-efficacy		No data		[128,129,146]	
	Individual stage of change		[95,121,124,125,127,138-140]		No data	
	Individual identification with organization		No data		[114,127,140]	
	Other personal attributes		[95,98,108,110,113-115,119,121, 125-129,131,133,135,138-144]		[114,127,129-131,139,140,143,144]	
**Domain 5: process**						
	Planning		[128,129]		[127,130]	
	**Engaging**		[128]		[120,128,129,144]	
		Opinion leaders	No data		No data	
		Formally appointed internal implementation leaders	[128]		No data	
		Champions	[129]		No data	
		External change agents	No data		[128,129]	
		Key stakeholders	[128,129]		[127,129,143]	
		Innovation participants	[128,129,138,144]		[115,129,143,144]	
	Executing		No data		No data	
	Reflecting and evaluative		No data		No data	

^a^CFIR: Consolidated Framework for Implementation Research.

#### Domain 1: Innovation Characteristics

##### Facilitators

Interventions that were developed at a trusted source (eg, academic institutions) [129] and originated as a research project [130] were viewed positively by those involved in implementation. Stakeholders and informal caregivers (hereafter referred to as caregivers) valued interventions based on pragmatic evidence [125,130]. However, caregivers also valued the incorporation of knowledge from those with lived experience (eg, other caregivers) as another form of evidence [114].

Interventions tended to be well-designed and easy to use [98,105,108-110,113,116,117,124-127,131-134,136-138,141,143,146]. There were mixed views on whether caregivers preferred dyadic or individual interventions [109,110,125,135,139,140]; however, there was generally a need for space to be created to allow caregivers to express themselves without the presence of care recipients [119,123]. The internet-based nature of interventions was generally well-accepted and offered some advantages over face-to-face interventions [114,117,120,126,127,130,136,​^139^,^142^], including (1) no need to travel [114,126] and (2) material being available all the time [127]. Interventions using different forms of media to deliver content [120,127,138,144] and supporting the use of interventions across devices (eg, ability to use intervention on a smartphone and computer) [132,144] made it easier for caregivers to use interventions. Tailored intervention content (eg, tailored to the care recipient’s stage of disease) [126,131,138,140,143,144], the provision of professional support [114,119,126,127,131-133,135,136,​^138^-^141^,^143^,^144^], a positive tone for information that was presented [125,127,138,140], and contact with information from other caregivers [114,120,126,127,131,132,136,138-140,​^143^,^144^] were important features to ensure interventions were relevant and met the needs of caregivers.

Interestingly, although caregivers expressed a preference for flexible use options, such as accessing modules on demand based on their needs [108,113,120,125-127,131,132,​^134^,^136^,^138^-^140^], stakeholders suggested a need to control module access to guide caregivers through the intervention and avoid confusion [127]. Stakeholders also valued interventions with features that supported user tracking to facilitate monitoring and evaluation of the implementation of the intervention [128] (also see inner setting: goals and feedback in Multimedia Appendix 5).

##### Barriers

Evidence suggesting interventions remained effective when implemented in real-world settings [128], and information on outcomes more relevant to health care organizations (eg, number of caregivers receiving support) [129,130] was lacking. Although e–mental health interventions have advantages, both caregivers and stakeholders expressed that communication within e–mental health interventions could be challenging [123,126,128,146]. Unfamiliar technologies and multistep intervention and implementation activities (eg, recruitment of intervention staff) added complexity to e–mental health interventions [113,128]. Stakeholders felt that the economic costs related to e–mental health interventions were unclear [129] and that interventions would not be cost-effective [128,147].

Interventions with content not appropriately tailored to the intervention user [95,114,124,126,127,131,132,135,136,140,142,​^144^] or that did not capture the diversity of caregivers’ backgrounds and care situations [114,124] made it challenging for caregivers to identify with the content. There was also a need for interventions to be linguistically and culturally tailored to meet the needs of different user groups within an implementation setting [143,144]. Interventions often did not meet all caregivers’ needs for support and information [95,98,110,114,117,120,123-127,130,133,135,136,138-140,143,144]. In addition, technical difficulties [98,108,114,118,124,​^126^,^129^,^130^,^144^] and limited viewing options [118,123-127,134,137,138,144], in particular, the lack of downloadable material so that the intervention could be used without active internet access, were important barriers to caregivers using the intervention more flexibly.

#### Domain 2: Outer Setting

##### Facilitators

Generally, stakeholders perceived intervention content to fit caregivers’ needs [128,130]. Stakeholders also stressed the need for support from cooperating organizations to facilitate implementation activities, with these activities potentially strengthening the relationship between partner organizations [128-130,143]. Additional facilitators included peer pressure due to digitalization in other sectors [129] and the fit between intervention and external policies (eg, informal care policies) [128,130].

##### Barriers

Stakeholders reported low interest in eHealth technologies among community members within their setting [129] and felt unsure what support services caregivers needed [128,129]. As mentioned, relationships with cooperating organizations were important to facilitate implementation [128-130,143]; however, partner organizations may not have the time available to support implementation [129].

#### Domain 3: Inner Setting

##### Overview

Barriers and facilitators within this domain were primarily derived from a series of reports investigating 2 interventions: (1) Partner in Balance, an intervention for informal caregivers of people with dementia [101,126-130] and (2) the Comprehensive Health Enhancement Support System (CHESS) intervention for caregivers of people with lung cancer, which contains a clinician report feature linking the caregiver-care recipient dyad to the care recipient’s health care provider [65,121-123].

##### Facilitators

Open and accessible communication channels within the implementation team facilitated intervention implementation [128]. Stakeholders viewed the ability to monitor intervention use as important to facilitate reporting on concrete intervention outputs [128]. The lack of support available for caregivers created an environment that was receptive to an intervention for caregivers [144]. Implementation was supported by the provision of training and support to stakeholders involved in intervention delivery [146]. Key facilitators related to the compatibility of the intervention within the implementation setting included (1) the integration of the intervention within existing workflows [127,128,143,146], (2) flexible use options for providers [122,127], and (3) alignment between the intervention and the organizations’ existing goals and priorities [128,129].

##### Barriers

Many barriers were not described in detail; however, a lack of internal support networks (eg, implementation teams) to support implementation [128,130], incentives [128], goal setting [128], leadership engagement [127-129], and resources (eg, staff time) [127-129] have been reported. Uncertainties due to upcoming organizational change and restructuring have made it difficult for some organizations to adopt a new intervention [127,129]. Barriers regarding compatibility were raised including the challenge of providing an intervention to caregivers within a system oriented toward patients [127,130], poor integration of the intervention within existing systems and processes [122,123], perceived low digital literacy among implementers [128], and a mismatch between the organizations’ clientele and the intervention target population [127,128]. In relation to the clinician report within the CHESS intervention adapted for lung cancer caregivers, unclear clinical guidance regarding how to use information provided within the intervention made it challenging for health care professionals to use the intervention [122,123].

#### Domain 4: Individual Characteristics

##### Facilitators

Interventions were identified as benefiting caregivers in many ways (eg, providing information or supporting self-care) [95,98,105,108,109,112,113,115-120,124-127,129-132,136-139,146], with some interventions also facilitating a sense of connection with other caregivers [113,114,120,136,140,143] and reducing feelings of isolation [113,114,124,127,131,133,136,143]. Interventions normalized and validated caregivers’ lived experiences by depicting scenarios that intervention users had personally experienced [114,124,125,127,131,132,135,136,​138,140,145]. Interventions, including support from a trained professional, were perceived as also benefiting the trained professional and had a positive impact on the relationship between the trained professional and caregiver [123,127-129,131,146]. Provision of support was viewed as an important source of motivation for caregivers to use the intervention [98,127,132,138,140]. It was perceived that interventions would be best suited for caregivers who were young [114,127,129,130,139], employed [127], experiencing difficulties (eg, burden) [139,140], or not receiving adequate support from their social network [140]. Stakeholders held positive views of e–mental health [98,129], perceiving interventions as time efficient [128,146] and as a way to improve access to support [131,146], including improving support for caregivers in rural communities [143].

##### Barriers

Both caregivers and stakeholders reported concerns related to e–mental health interventions, including privacy and liability concerns [98,120-123,128], feeling that web-based interventions are impersonal [95,113,114,126,138,139,142], and concerns that the intervention could have a negative impact (eg, increase isolation) [98,120,126,127,133] or be emotionally challenging [95,117,126,127,138-140] for caregivers. Stakeholders had little experience with eHealth technologies, making implementation challenging [129]. In addition, some stakeholders found it challenging to create a therapeutic relationship within the e–mental health intervention [146]. For some caregivers, the intervention had come at the wrong time (eg, the information was no longer relevant or they had already moved past certain challenges) [95,121,124,125,127,138,140], whereas others were not ready to accept help [121,140] or face difficult emotions that an intervention may prompt [95,127,139]. Caregivers also reported individual challenges engaging with e–mental health interventions such as having too many other responsibilities [108,110,113-115,125,127,131,135,138,139,142-144], feelings of shame stopping them from sharing their experiences [119,127,138], and care recipient related challenges (eg, not wanting their care recipient to know they are receiving the intervention) [98,126,127,140]. Some caregivers were not in need of an e–mental health intervention [95,121,135,138-140,142] and had their support needs met in other ways [138-141]. Low digital literacy, and lack of access to internet or computers among caregivers were also challenges for caregivers using e–mental health interventions [95,114,121,126-129,133,140,142-144]. In contrast, 1 study [144] reported that caregivers’ skills and familiarity with using the internet could facilitate implementation.

#### Domain 5: Process

##### Facilitators

Financial planning [127,130] and a sense of ownership toward the intervention [128,129] were perceived as facilitators of implementation. Engaging external organizations to assist with intervention implementation [128,129] and training and retention of key staff members who deliver the intervention [127,129] were important elements of the implementation process. Strategies to reach a diverse group of caregivers from different backgrounds were perceived as valuable by both caregivers and stakeholders [115,129]. Engagement strategies included the engagement of caregivers and stakeholders in early decision-making stages [129,143], seeking feedback from caregivers about the intervention [144], engaging the entire informal care network (eg, rather than focusing on primary caregivers) as potential users of the intervention [143], and speaking with caregivers face-to-face to build a connection with caregivers and integrate intervention referral into any interaction professionals have with caregivers [129,143].

##### Barriers

Inadequate implementation planning [128,129], low engagement of organization leaders [128], intervention champions [129], intervention providers [128,129], and informal caregivers [128,129,144] were barriers to implementation. Both caregivers and stakeholders felt face-to-face strategies to engage caregivers in the intervention (eg, during recruitment or at the start of the intervention) were needed [138]. In 1 study [128], training provided to intervention providers lacked focus on the practical skills needed to implement and deliver the intervention.

### Professional Stakeholder Involvement

#### Overview

Professional stakeholders (n=4) with experience working in the field of eHealth and e–mental health recognized many barriers and facilitators identified in the thematic synthesis. For example, stakeholders had encountered facilitators such as (1) e–mental health and eHealth interventions being easy to use, (2) the ability of interventions to easily facilitate data collection, and (3) national policy changes enabling flexible implementation of e–mental health interventions throughout a country. Stakeholders also recognized several barriers to implementation identified in the thematic synthesis, such as (1) negative views about e–mental health, (2) technical difficulties, and (3) lack of organizational incentives to implement e–mental health interventions.

However, stakeholders also identified additional implementation barriers and facilitators they have experienced and areas they felt that future research should focus on.

#### Facilitators

Intervention characteristics, including (1) the ability to adapt interventions to expand their use among different populations and (2) low complexity for stakeholders to provide interventions, were facilitating factors when implementing e–mental health interventions in practice. Within the inner setting, the provision of training materials was a facilitator. e–Mental health interventions were also perceived to add variety to work routines of staff involved in intervention delivery. Engagement of formally appointed implementation leaders, endorsement of the intervention by influencers or celebrities, and a positive web-based presence (eg, reviews and social media presence) were valuable to facilitate implementation.

#### Barriers

Key factors related to the inner setting were referred to as additional barriers, including (1) changes to work routines that negatively impact how well the intervention fits within the implementation setting; (2) changing the priority of the intervention as it competes with other interventions and initiatives for resources; (3) challenging implementation climate due to staff being overwhelmed by numerous new digital tools; and (4) poor fit between the intervention and internal policies and regulations. Barriers related to negative knowledge and beliefs regarding e–mental health interventions were identified within the thematic synthesis; however, one negative belief not reported in the literature was the perception that e–mental health interventions are a cheaper but not more effective alternative to face-to-face interventions. Linked to this was the importance of recognizing that e–mental health interventions should be offered as a choice to users, rather than the sole treatment option available. The lack of realistic implementation planning and knowledge about the amount of resources needed to implement an e–mental health intervention was also a challenge. Lack of evidence and knowledge about implementation strategies that effectively enhance the implementation of e–mental health interventions was also viewed as a barrier to implementation.

#### Areas for Future Research

Professional stakeholders identified the following areas for future research: (1) how to best combine e–mental health interventions with other interventions (both eHealth and face-to-face interventions); (2) defining core elements needed for e–mental health interventions to maintain effectiveness; (3) methods to maximize the engagement and retention of e–mental health users; (4) how to influence individuals’ views of e–mental health; and (5) further research on the benefits of eHealth for both users and stakeholders (eg, how they impact quality of care).

## Discussion

### Principal Findings

#### Overview

This mixed methods systematic review identified 53 reports that investigated the effectiveness or implementation of e–mental health interventions for informal caregivers of adults with chronic diseases. Interventions were most often tailored for informal caregivers of people with dementia or cancer, with few interventions focused on informal caregivers of people with other chronic conditions included in this review (chronic obstructive pulmonary disease, diabetes, heart disease, and stroke). Interventions were commonly theory based and varied in terms of the type of support provided to intervention users. A unique type of support identified in this review was tailored standardized support, which provides intervention users with standardized support messages, but tailors messages based on information provided by an individual user.

Overall, 14 RCTs were included in the review. RCTs contained a mixture of pragmatic and explanatory design features, as assessed using the PRECIS-2 tool, and most were evaluated as having a high risk of bias (discussed further in the *Limitations* section). The PRECIS-2 scoring showed that the domains of intervention delivery and adherence were very pragmatic, with no measures to ensure adherence to the intervention beyond what would be expected outside a trial environment. The trial setting was often pragmatic because most trials allowed participants to be located in a variety of geographic areas and did not focus on a single recruitment site. Trials commonly used a variety of recruitment methods (eg, via health care and community settings), which was viewed as a pragmatic design choice, given that informal caregivers would ideally be able to find out about available support services through a variety of pathways. Despite PRECIS-2 scores demonstrating that all RCTs contained some pragmatic design features, RCTs were frequently conducted within academic settings without indications as to how the interventions could be integrated into routine practice.

The QCA could not be fully conducted because of low consistency in which conditions were sufficient for intervention effectiveness. In cases where consistency was high enough to proceed with the analysis, solution coverage (ie, coverage of the set of conditions with a consistency of at least 0.75) was low, as the solutions were based on only 1 to 2 RCTs. The challenges encountered in the QCA analysis were mainly due to the low number of RCTs (n=14) included in the analysis. In addition, poor reporting of key intervention features posed a challenge to including implementation-related conditions in the QCA analysis.

Poor reporting of key intervention features and intervention targets presented a challenge in determining whether interventions were designed to target informal caregivers’ mental health. For example, in one case, the intervention target differed across cultural adaptations of the intervention [99,150,151]; however, the rationale as to why intervention targets differed and whether this impacted the intervention content was unclear. Poor intervention reporting is a common problem in the wider literature, despite the development of reporting guidelines such as CONSORT (Consolidated Standards of Reporting Trials) [152] and Template for Intervention Description and Replication (TIDieR) [153]. In 1 review of reviews [154], it was found that almost 88% of the included studies had below-optimal levels of reporting in accordance with CONSORT. Another review of reviews [155] showed variation in reporting quality based on each item of the TIDieR. Incomplete reporting based on the TIDieR was most often observed regarding (1) intervention modifications, (2) planned and actual intervention adherence and fidelity, (3) tailoring, (4) descriptions of the intervention provider, and (5) where the intervention occurred [155]. Poor reporting in some of these areas was observed in this review. For example, a description of the training provided to intervention providers was often vague or absent, and fidelity and adherence to the intervention among intervention providers and users was underreported. A similar finding was reported in a study using the ImpRess checklist to assess the implementation readiness of 12 eHealth interventions for informal dementia caregivers [28]. Poor intervention reporting may pose a challenge to future implementation given that the details needed to implement and deliver interventions are lacking.

#### Implementation Barriers and Facilitators

Most identified barriers and facilitators were related to the intervention or individual characteristic domains within the CFIR. Implementation determinants related to the inner and outer setting and the implementation process were rarely reported. The lack of information on implementation barriers and facilitators related to the implementation setting can be partly related to the nature of the included reports, which commonly focused on intervention acceptability during the development or adaptation of an intervention. Although implementation determinants were reported in development studies, these determinants represent anticipated barriers and facilitators, rather than actual barriers and facilitators encountered during implementation. Intervention implementation outside of a research setting was rarely explored. Other reviews focused on the implementation of eHealth interventions for different groups of informal caregivers [27,46,47] similarly found that there was a lack of reporting of implementation determinants related to the inner and outer setting.

Overall, the literature indicates that intervention development is done well, with the views of informal caregivers and stakeholders being included in the development process in a variety of ways (eg, surveys, focus groups, and usability testing). This aligns with the current Medical Research Council framework for developing and evaluating complex interventions, which places the engagement of all stakeholders (including intervention users) as a core element that should be included in each phase of intervention development and evaluation [48]. Although intervention development did engage stakeholders, data collection rarely explored implementation barriers and facilitators with stakeholders beyond intervention acceptability. As such, many aspects of another core element of the current Medical Research Council framework, that is, context, remain largely unexplored [48].

Recruitment of informal caregivers in intervention research is well-established as challenging [121,156-158], and this challenge can persist after interventions are implemented. Within the CFIR, recruitment of intervention users falls within the construct *engaging* under domain 5: process. Recruitment strategies, such as face-to-face contact, were identified as potential facilitators of the implementation of e–mental health interventions. However, the effectiveness of strategies to recruit and sustain intervention engagement was not explored. Strategies to recruit informal caregivers and improve awareness of available e–mental health interventions require further research, and critical examination of whether e–mental health interventions are being accessed by informal caregivers experiencing mental health difficulties should be explored in future studies.

Several barriers to and facilitators of implementation indicated the importance of tailoring intervention content, visuals, and support for informal caregivers’ individual needs and preferences. Internet-based interventions offer not only opportunities to tailor intervention content but also preferences regarding information delivery format (eg, video, audio, and text) [159]. In addition, tailoring has been shown to have a positive impact on the effectiveness of behavior change interventions [160]. Tailoring should be explored as an approach to enhance effectiveness and user engagement with e–mental health interventions.

#### Reflection on Updates to the CFIR

In 2022, after the thematic synthesis for this review was completed, an addendum to the CFIR was published [161] and an updated version of the CFIR was produced [162]. In the addendum, the authors specified that the CFIR is not appropriate for data from intervention users (ie, informal caregivers within the context of this review) unless intervention users play a role in intervention delivery or implementation [161]. CFIR authors classified data from intervention users as representing innovation determinants rather than implementation determinants [161]. The decision to exclude data from intervention users from the CFIR framework was motivated by the implementation of interventions primarily influenced by professional stakeholders involved more directly in activities related to intervention delivery or implementation [161]. Considering this addendum, it could be argued that the data included in this thematic synthesis, derived from informal caregivers, should have been excluded. However, given that e–mental health interventions rely on informal caregivers using interventions independently in their home environment, the perspective of informal caregivers could influence implementation and sustainability.

The CFIR defines contextual factors that can influence implementation; however, context is a broad concept, and different definitions and frameworks exist to define it [49]. In a scoping review that sought to review multiple implementation frameworks to comprehensively define the dimensions of implementation context [49], context was divided into 3 levels: micro, meso, and macro. Intervention users (eg, their views, needs, and preferences) were considered to fall within the microlevel [49]. Factors related to the implementing organization were classified within the mesolevel, with the wider implementation setting representing the macrolevel [49]. The CFIR framework captures the mesolevel and macrolevel of context; however, it does not include microlevel contextual factors. Researchers may wish to consider whether microlevel contextual factors may be important to consider in their implementation context.

### Limitations

RCTs were retrospectively assessed using the PRECIS-2 tool to evaluate how pragmatic or explanatory each trial design was. Although the PRECIS-2 tool can be used retrospectively [163], poor reporting regarding the intended implementation context of the intervention under investigation within the trial posed a challenge to using the PRECIS-2 tool accurately. PRECIS-2 scores are dependent on understanding the intended implementation context of each intervention to assess how pragmatic design decisions within the trial were given the intended implementation context [39]. The intended implementation context is not often described in RCTs; therefore, assumptions about the intended implementation context were made to facilitate the PRECIS-2 scoring. For example, reviewers assumed that interventions were generally intended to be implemented across the entire country, unless otherwise specified in the trial. As the authors of the PRECIS-2 tool recognize [163], the use of the CONSORT extension for pragmatic trials [164] would facilitate the retrospective assessment of RCTs using PRECIS-2.

Risk of bias was evaluated using the Cochrane Risk of Bias 2.0 tool [81] in line with recommendations from the Cochrane Handbook for Systematic Reviews of Interventions [165]. However, research has shown that the Risk of Bias 2.0 tool can have low interrater reliability [166], which may impact the interpretation of the risk-of-bias assessments included in this review. Domain 4 of the Risk of Bias 2.0 assessment was often rated as high because the outcome assessors (which in the context of self-reported outcomes is the participant) were not blinded. The blinding of outcome assessors and others involved in RCTs is often a challenge, especially for RCTs of mental health interventions [167]. Various approaches to blinding participants and intervention providers in mental health trials have been proposed (eg, recruiting participants with no knowledge of mental health interventions, requiring that intervention providers have limited experience with mental health interventions); however, these approaches can be difficult to implement and have a negative impact on how pragmatic and generalizable the trial is [167,168]. Although the lack of blinding is a source of bias, participants not being blinded is a more pragmatic design choice and more closely reflects the conditions that could be expected if interventions were used in real-world settings.

Although OpenGrey was searched for gray literature, relevant gray literature, such as government reports, may have been missed, as these reports are not included in OpenGrey, and certain gray literature publication types, such as theses and abstracts, were not eligible for inclusion in this review. In addition, as searches were only conducted using English terms, gray literature in languages other than English may have been difficult to capture.

As discussed, given the recent addendum to the CFIR [161], some data included in the thematic synthesis (ie, data from informal caregivers) may not be universally considered relevant for implementation. However, this information provides important insights into the views of informal caregivers within the microlevel of context [49] and provides guidance on important design and implementation characteristics to consider to ensure the acceptability and uptake of e–mental health interventions among informal caregivers.

### Conclusions

Although considerable attention has been given to the usability and acceptability of e–mental health interventions for informal caregivers of adults with chronic diseases, few studies have explored other factors that may influence implementation. In particular, factors related to outer and inner implementation settings and the implementation process have rarely been explored. The views of professional stakeholders who are or will be involved in intervention implementation or delivery should be investigated to fill this gap. Given the challenges faced by e–mental health interventions when implemented in practice, implementation science research exploring not only implementation determinants but also implementation strategies are urgently needed.

## Appendix

Multimedia Appendix 1 PRISMA (Preferred Reporting Items for Systematic Reviews and Meta-Analyses) checklists.

Multimedia Appendix 2 Outcomes, Consolidated Framework for Implementation Research, search, support taxonomy.

Multimedia Appendix 3 Reasons for exclusion.

Multimedia Appendix 4 Intervention characteristics, qualitative comparative analysis, Pragmatic Explanatory Continuum Indicator Summary 2, and risk of bias.

Multimedia Appendix 5 Barriers and facilitators.

## Abbreviations

CFIR: Consolidated Framework for Implementation Research
CHESS: Comprehensive Health Enhancement Support System
CONSORT: Consolidated Standards of Reporting Trials
PICOS: population, intervention, comparators, outcomes, study designs
PRECIS-2: Pragmatic Explanatory Continuum Indicator Summary 2
PRISMA: Preferred Reporting Items for Systematic Reviews and Meta-Analyses
PRISMA: Preferred Reporting Items for Systematic Reviews and Meta-Analyses-literature search extension
PROSPERO: International Prospective Register of Systematic Reviews
QCA: qualitative comparative analysis
RCT: randomized controlled trial
TIDieR: Template for Intervention Description and Replication
