# Supplementary material for: Implementation of e–Mental Health Interventions for Informal Caregivers of Adults With Chronic Diseases: Mixed Methods Systematic Review With a Qualitative Comparative Analysis and Thematic Synthesis
Source: JMIR Ment Health. 2022 Nov 30;9(11):e41891. doi: 10.2196/41891 (PMC9752475; doi:10.2196/41891)
Supplement: Multimedia Appendix 2 [file mental_v9i11e41891_app2.pdf]

Multimedia Appendix 2

- Table 2.1: Quality of included primary outcome measurements
- Figure 2.1: Consolidated Framework for Implementation Research
- Table 2.2: PubMed search strategy
- Table 2.3: Taxonomy of support

Table 2.1: Quality of included primary outcome measurements

**Outcome measurement**	**Internal consistency** (Cronbach's alpha)
CES-D	0.85<br>(Radloff, 1977)
GHQ	0.75-0.90<br>(Goldberg & Williams, 1988 & Hankins, 2008)
HADS	> 0.80 for each subscale<br>(Zigmond and Snaith, 1983 & Bjelland et al, 2002)
PHQ	0.89<br>(Kroenke et al, 2001)
PSS	0.85<br>(Cohen et al, 1983)
Negative mood[a]	0.95<br>(DuBenske et al, 2014)

Abbreviations: CES-D: Center for Epidemiological Studies – Depression scale; GHQ: General Health Questionnaire; HADS: Hospital Anxiety and Depression Scale; HADS-D: Hospital Anxiety and Depression Scale – Depression subscale; PHQ: Patient Health Questionnaire depression scale; PSS: Perceived Stress Scale

[a]Negative mood was based on a modified version of the Short Version – Profile of Mood States

References

1. Bjelland, I., Dahl, A. A., Haug, T. T., & Neckelmann, D. (2002). The validity of the Hospital Anxiety and Depression Scale. An updated literature review. Journal of psychosomatic research, 52(2), 69–77. https://doi.org/10.1016/s0022-3999(01)00296-3
2. Cohen, S., Kamarck, T., & Mermelstein, R. (1983). A global measure of perceived stress. Journal of health and social behavior, 24(4), 385–396.
3. DuBenske, L. L., Gustafson, D. H., Namkoong, K., Hawkins, R. P., Atwood, A. K., Brown, R. L., Chih, M. Y., McTavish, F., Carmack, C. L., Buss, M. K., Govindan, R., & Cleary, J. F. (2014). CHESS improves cancer caregivers' burden and mood: results of an eHealth RCT. Health psychology: official journal of the Division of Health Psychology, American Psychological Association, 33(10), 1261–1272. https://doi.org/10.1037/a0034216
4. Goldberg DP, Williams P: A user's guide to the General Health Questionnaire. 1988, Basingstoke NFER-Nelson
5. Hankins M. (2008). The reliability of the twelve-item general health questionnaire (GHQ-12) under realistic assumptions. BMC public health, 8, 355. https://doi.org/10.1186/1471-2458-8-355

6. Kroenke, K., Spitzer, R. L., & Williams, J. B. (2001). The PHQ-9: validity of a brief depression severity measure. Journal of general internal medicine, 16(9), 606–613. https://doi.org/10.1046/j.1525-1497.2001.016009606.x
7. Radloff, L. S. (1977). The CES-D Scale: A Self-Report Depression Scale for Research in the General Population. Applied Psychological Measurement, 1(3), 385–401. https://doi.org/10.1177/014662167700100306
8. Zigmond, A. S., & Snaith, R. P. (1983). The hospital anxiety and depression scale. Acta psychiatrica Scandinavica, 67(6), 361–370. https://doi.org/10.1111/j.1600-0447.1983.tb09716.x

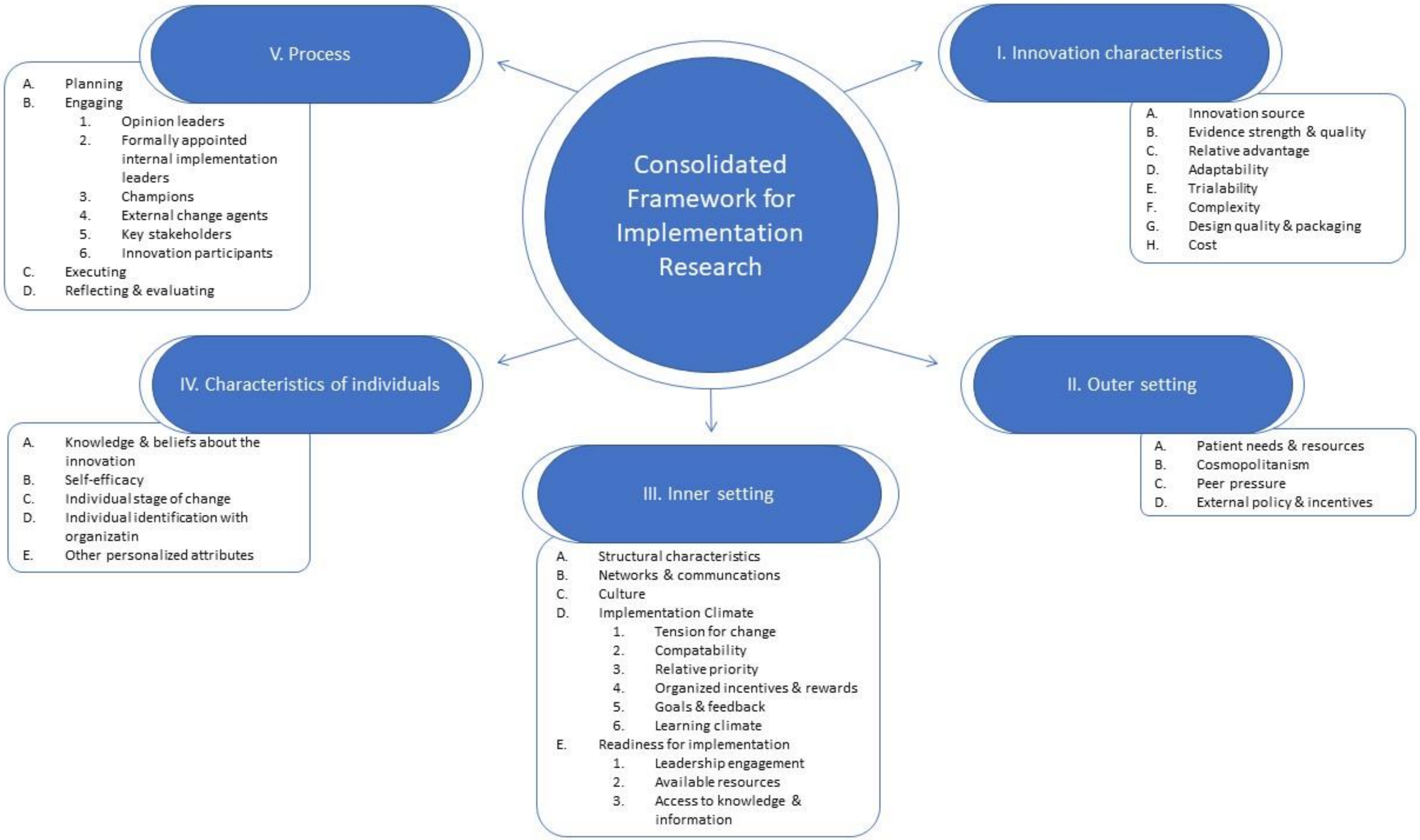

Figure 2.1: Consolidated Framework for Implementation Research. Adapted from Damschroder, L. J., Aron, D. C., Keith, R. E., Kirsh, S. R., Alexander, J. A., & Lowery, J. C. (2009). Fostering implementation of health services research findings into practice: a consolidated framework for advancing implementation science. Implementation science, 4, 50. https://doi.org/10.1186/1748-5908-4-50

Table 2.2: PubMed search strategy

#	
**1**	caregiver*[Title/Abstract] OR<br>care-giver*[Title/Abstract] OR<br>carer*[Title/Abstract] OR<br>family[Title/Abstract] OR<br>families[Title/Abstract] OR<br>spous*[Title/Abstract] OR<br>sibling*[Title/Abstract] OR<br>husband*[Title/Abstract] OR<br>wife[Title/Abstract] OR<br>wives[Title/Abstract] OR<br>partner[Title/Abstract] OR<br>partners[Title/Abstract] OR<br>parents[Title/Abstract] OR<br>parent[Title/Abstract] OR<br>friend[Title/Abstract] OR<br>friends[Title/Abstract] OR<br>relative[Title/Abstract] OR<br>relatives[Title/Abstract] OR<br>couple[Title/Abstract] OR<br>couples[Title/Abstract] OR<br>mother*[Title/Abstract] OR<br>father*[Title/Abstract] OR<br>support person*[Title/Abstract] OR<br>next of kin[Title/Abstract] OR<br>significant other*[Title/Abstract] OR<br>caregivers[MeSH terms] OR<br>family[MeSH terms] OR<br>spouses[MeSH terms] OR<br>siblings[MeSH terms] OR<br>parents[MeSH terms] OR<br>friends[MeSH terms] OR<br>mothers[MeSH terms] OR<br>fathers[MeSH terms]
**2**	cancer[Title/Abstract] OR<br>tumor[Title/Abstract] OR<br>tumors[Title/Abstract] OR<br>tumours[Title/Abstract] OR<br>tumour[Title/Abstract] OR<br>neoplasm*[Title/Abstract] OR<br>oncology[Title/Abstract] OR<br>carcinoma[Title/Abstract] OR<br>malignan*[Title/Abstract] OR<br>melanoma[Title/Abstract] OR<br>metastasis[Title/Abstract] OR<br>lymphoma[Title/Abstract] OR<br>leukemia[Title/Abstract] OR<br>diabetes[Title/Abstract] OR<br>COPD[Title/Abstract] OR<br>chronic obstructive pulmonary disease[Title/Abstract] OR<br>COAD[Title/Abstract] OR<br>chronic obstructive airway disease[Title/Abstract] OR<br>chronic obstructive lung disease[Title/Abstract] OR

	chronic airflow obstruction[Title/Abstract] OR<br>stroke[Title/Abstract] OR<br>cerebrovascular accident[Title/Abstract] OR<br>CVA[Title/Abstract] OR<br>acute cerebrovascular accident[Title/Abstract] OR<br>brain vascular accident[Title/Abstract] OR<br>apoplexy[Title/Abstract] OR<br>heart disease[Title/Abstract] OR<br>cardiovascular disease[Title/Abstract] OR<br>CVD[Title/Abstract] OR<br>ischemic heart disease[Title/Abstract] OR<br>IHD[Title/Abstract] OR<br>coronary artery disease[Title/Abstract] OR<br>CAD[Title/Abstract] OR<br>angina[Title/Abstract] OR<br>myocardial infarction[Title/Abstract] OR<br>MI[Title/Abstract] OR<br>heart attack[Title/Abstract] OR<br>cardiac event[Title/Abstract] OR<br>cardiac disease[Title/Abstract] OR<br>dementia[Title/Abstract] OR<br>alzheimer*[Title/Abstract] OR<br>FTD[Title/Abstract] OR<br>Lewy body[Title/Abstract] OR<br>Lewy bodies[Title/Abstract] OR<br>neurocognitive disorder*[Title/Abstract] OR<br>MCI[Title/Abstract] OR<br>mild cognitive impairment[Title/Abstract] OR<br>memory impair*[Title/Abstract] OR<br>cognitive decline[Title/Abstract] OR<br>chronic illness*[Title/Abstract] OR<br>chronic disease*[Title/Abstract] OR<br>chronic condition*[Title/Abstract] OR<br>multi-morbidit*[Title/Abstract] OR<br>multimorbidit*[Title/Abstract] OR<br>long-term health condition*[Title/Abstract] OR<br>neoplasms[MeSH terms] OR<br>diabetes mellitus[MeSH terms] OR<br>pulmonary disease, chronic obstructive[MeSH terms] OR<br>stroke[MeSH terms] OR<br>cardiovascular diseases[MeSH terms] OR<br>dementia[MeSH terms] OR<br>cognition disorders[MeSH terms] OR<br>chronic disease[MeSH terms] OR<br>multimorbidity[MeSH terms]
**3**	eHealth[Title/Abstract] OR<br>e-health[Title/Abstract] OR<br>e-mental health[Title/Abstract] OR<br>emental health[Title/Abstract] OR<br>mhealth[Title/Abstract] OR<br>m-health[Title/Abstract] OR<br>smartphone[Title/Abstract] OR<br>cell phone[Title/Abstract] OR<br>cellular phone[Title/Abstract] OR<br>mobile phone[Title/Abstract] OR

	app[Title/Abstract] OR<br>apps[Title/Abstract] OR<br>application[Title/Abstract] OR<br>applications[Title/Abstract] OR<br>iPad[Title/Abstract] OR<br>computer[Title/Abstract] OR<br>tablet[Title/Abstract] OR<br>technology[Title/Abstract] OR<br>technologies[Title/Abstract] OR<br>electronic communication[Title/Abstract] OR<br>email[Title/Abstract] OR<br>e-mail[Title/Abstract] OR<br>text messag*[Title/Abstract] OR<br>internet[Title/Abstract] OR<br>wireless[Title/Abstract] OR<br>online[Title/Abstract] OR<br>digital[Title/Abstract] OR<br>on-line[Title/Abstract] OR<br>virtual[Title/Abstract] OR<br>ICT[Title/Abstract] OR<br>web[Title/Abstract] OR<br>website[Title/Abstract] OR<br>multimedia[Title/Abstract] OR<br>e-learning[Title/Abstract] OR<br>online social network[Title/Abstract] OR<br>iCBT[Title/Abstract] OR<br>cCBT[Title/Abstract] OR<br>e-therap*[Title/Abstract] OR<br>etherap*[Title/Abstract] OR<br>cell phone[MeSH terms] OR<br>mobile application[MeSH terms] OR<br>computers[MeSH terms] OR<br>therapy, computer assisted[MeSH terms] OR<br>wireless technology[MeSH terms] OR<br>information technology[MeSH terms] OR<br>technology[MeSH terms] OR<br>electronic mail[MeSH terms] OR<br>internet[MeSH terms] OR<br>online social networking[MeSH terms] OR<br>virtual reality[MeSH terms] OR<br>web browser[MeSH terms] OR<br>multimedia[MeSH terms]
**4**	mental health[Title/Abstract] OR<br>mood[Title/Abstract] OR<br>depress*[Title/Abstract] OR<br>affective disorder[Title/Abstract] OR<br>negative affect[Title/Abstract] OR<br>dysthymia[Title/Abstract] OR<br>dysphoria[Title/Abstract] OR<br>melancholic[Title/Abstract] OR<br>anxiety[Title/Abstract] OR<br>burden[Title/Abstract] OR<br>distress[Title/Abstract] OR<br>stress[Title/Abstract] OR<br>well being[Title/Abstract] OR

	emotion[Title/Abstract] OR<br>emotional[Title/Abstract] OR<br>mood disorders[MeSH terms] OR<br>depression[MeSH terms] OR<br>anxiety[MeSH terms] OR<br>anxiety disorders[MeSH terms] OR<br>stress, psychological[MeSH terms] OR<br>emotions[MeSH terms] OR<br>mental health[MeSH terms]
**5**	mental health service*[Title/Abstract] OR<br>mental healthcare[Title/Abstract] OR<br>therapy[Title/Abstract] OR<br>therapies[Title/Abstract] OR<br>treatment*[Title/Abstract] OR<br>intervention*[Title/Abstract] OR<br>program*[Title/Abstract] OR<br>psychological[Title/Abstract] OR<br>psychologist[Title/Abstract] OR<br>psychoeducation[Title/Abstract] OR<br>psycho-education[Title/Abstract] OR<br>CBT[Title/Abstract] OR<br>health education[Title/Abstract] OR<br>problem solving[Title/Abstract] OR<br>PST[Title/Abstract] OR<br>ACT[Title/Abstract] OR<br>behaviour*[Title/Abstract] OR<br>therapist[Title/Abstract] OR<br>support[Title/Abstract] OR<br>mindful*[Title/Abstract] OR<br>psychotherapy[Title/Abstract] OR<br>relaxation[Title/Abstract] OR<br>meditation[Title/Abstract] OR<br>behavior*[Title/Abstract] OR<br>counseling[Title/Abstract] OR<br>counselling[Title/Abstract] OR<br>cognitive reframing[Title/Abstract] OR<br>cognitive restructuring[Title/Abstract] OR<br>self-help[Title/Abstract] OR<br>self-management[Title/Abstract] OR<br>blended[Title/Abstract] OR<br>mental health services[MeSH terms] OR<br>health education[MeSH terms] OR<br>problem solving[MeSH terms] OR<br>psychotherapy[MeSH terms] OR<br>relaxation[MeSH terms] OR<br>self-management[MeSH terms] OR<br>self-help groups[MeSH terms]
**6**	"2007/01/01"[Date - Publication] : "3000"[Date - Publication]
**7**	#1 AND #2 AND #3 AND #4 AND #5 AND #6

Table 2.3: Taxonomy of support (adapted from Glasgow & Rosen, 1978, Farrand & Woodford, 2013).

Category	Definition
Self-Administered	Following initial session introducing self-help intervention, patient uses intervention exclusively on their own without support, with the exception of potential on-going contact for data collection purposes only.
Guided	Regular scheduled support sessions with a practitioner that has knowledge of specific factors to identify patients understanding regarding theoretical rationale for intervention, reflection on techniques adopted, aid problem solving where difficulty using techniques, using common factors to encourage progress through intervention.
Minimal Contact	Patient relies exclusively upon self-help intervention with regular brief practitioner supported sessions to monitor use, encourage engagement and direct progress but does not use specific factors to engage patient reflection on techniques adopted. In the event of difficulties engaging with techniques, practitioner uses common factor skills to direct patient back to the relevant part of the self-help intervention.
Standardized	Standardized prompts and reminders sent directly by online or app-based self-help intervention or through standardized emails sent by practitioner to prompt use and offer additional support upon request. Prompts and reminders may adopt common factor skills to provide encouragement or empathy where patient is struggling to engage with the intervention or outcome measures indicate no or little improvement.
Tailored standardized	The same as standardized, however, prompt and reminders are automatically tailored based on patient’s responses or characteristics.

References

Glasgow, R. E., & Rosen, G. M. (1978). Behavioral bibliotherapy: a review of self-help behavior therapy manuals. Psychological bulletin, 85(1), 1–23.

Farrand, P., & Woodford, J. (2013). Impact of support on the effectiveness of written cognitive behavioural self-help: a systematic review and meta-analysis of randomised controlled trials. Clinical psychology review, 33(1), 182–195. https://doi.org/10.1016/j.cpr.2012.11.001
