# Supplementary material for: Implementation of e–Mental Health Interventions for Informal Caregivers of Adults With Chronic Diseases: Mixed Methods Systematic Review With a Qualitative Comparative Analysis and Thematic Synthesis
Source: JMIR Ment Health. 2022 Nov 30;9(11):e41891. doi: 10.2196/41891 (PMC9752475; doi:10.2196/41891)
Supplement: Multimedia Appendix 3 [file mental_v9i11e41891_app3.pdf]

Multimedia Appendix 3

Table 3.1: Reasons for exlusion of reports assessed for eligibility

Primary reason for exclusion	Excluded full text
Wrong population	**Care recipient at end of life**<br>Leow & Chan, 2016<br>**Includes formal caregivers**<br>Alkhushayni & McRoy, 2016; Ferré-Bergadà et al., 2021<br>**Other chronic conditions**<br>Ferré-Grau et al., 2021
Wrong intervention	**Intervention did not target the caregiver's mental health**<br>Aguado Loi et al., 2017; Applebaum et al., 2018; Bartels et al., 2019; Bateman et al., 2017; Becker & Webbe, 2008; Blanton et al., 2018; Bowman et al., 2015; Burstein et al., 2015; Callan et al., 2016; Caunca et al., 2018; Chiu et al., 2010; Chiu & et al, 2010; Czaja et al., 2013; Czaja et al., 2014; Dam et al., 2019; Davis et al., 2015; Droes et al., 2019; Duggleby et al., 2017; Duggleby et al., 2019; Fergus et al., 2014; Fergus et al., 2022a, 2022b; Finkel et al., 2007; Fowler et al., 2014; Fowler et al., 2016; Gaugler, et al., 2015; Halbach et al., 2018; Han et al., 2022; Hattink et al., 2016; Heynsbergh et al., 2019; Holroyd-Leduc et al., 2017; Ho et al., 2015; Kales et al., 2017b, 2018; Lambert et al., 2020; Li et al., 2021; Loh et al., 2018;; Metcalfe et al., 2019; Nunez-Naveira et al., 2016; Pagan-Ortiz et al., 2014; Papadakos et al., 2017; Perales-Puchalt et al., 2022a, 2022b; Piette et al., 2015; Piil et al., 2015; Ploeg et al., 2018; Rathnayake et al., 2019; Reeves et al., 2019; Ruggiano et al., 2019; Rutz et al., 2019; Santin et al., 2018; Sarkar et al., 2016a; Schaller et al., 2016; Steele Gray et al., 2014; Stevenson et al., 2020; Sun et al., 2016; Taylor & Pagliari, 2019; Tixier et al., 2009; Toot et al., 2012; Torp et al., 2008; Tyack et al., 2017; Weiss et al., 2013; Wittenberg et al., 2019; Yuce & Gulkesen, 2013; Zafeiridi et al., 2018; Zhu et al., 2018<br><br>**Intervention did not meet our definition of e-mental health**<br>***Online forum***

	Male et al., 2017; McKechnie et al., 2014; Oliffe et al., 2015; Pierce et al., 2009; Scharett et al., 2017; Stephen et al., 2014; Stephen, et al., 2013<br>***Email based***<br>Meichsner et al., 2019; Meier et al., 2007<br>***Video-call based***<br>Han et al., 2021; Kubo et al., 2020; Loi et al., 2022; McEvoy et al., 2019; Moskowitz et al., 2019; Yousefi et al., 2022<br>***Telephone based***<br>LeLaurin et al., 2021<br>***Virtual chat group***<br>O'Connor et al., 2014<br>***Not internet-based***<br>Agren et al., 2012; Paterson et al., 2019; Scott et al., 2015; Steffen & Gant, 2016; Zauszniewski et al., 2016<br><br>**Not e-mental health specific**<br>Murray et al., 2007; Shreve et al., 2016<br><br>**Intervention is not a mental health treatment**<br>Boele et al., 2022; Gaugler et al., 2016; Golden et al., 2017; McCarron et al., 2019; Wilkerson et al., 2018<br><br>**No intervention**<br>Ploeg et al., 2019; Reblin et al., 2018a, 2018b; Robillard et al., 2018; Shapiro et al., 2016
Not an RCT and no report on implementation	Chien et al., 2020; Griffiths et al., 2018; Kishita et al., 2021; Kwok et al., 2014; Lorig et al., 2012
Wrong outcome	Bricoli, 2015; Fitzgerald et al., 2011; Kovaleva et al., 2022; Pot et al., 2019; Steiner et al., 2008; Telke et al., 2022
Wrong study type	**Conference abstract**<br>Allemann et al., 2018; Applebaum et al., 2016; Applebaum et al., 2017; Atherton et al., 2013; Blom & Pot, 2013; Boele et al., 2021a, 2021b; Bohnak & Barron, 2017; Bonneux et al., 2019; Breen et al., 2016; Brinkert, 2021; Brungardt et al., 2019; Bryant et al., 2013; Buzaglo et al., 2015; Caunca et al., 2017; Chan et al., 2016; Czaja et al., 2011; Dam et al., 2015; Donovan et al., 2021; DuBenske et al., 2010; Farooqi et al., 2021; Fergus et al., 2013; Fossey et al., 2017; Freddolino et al., 2018; Gies, 2011; Grapp et al., 2020; Gunn et al., 2012, 2013; Hammer & Klein,

	2012; Jacobs, 2020; Kajiyama et al., 2018; Kales, 2017a; Kales et al., 2016; Koehle et al., 2015; Köhle et al., 2018; Kubo et al., 2017; Langbecker & Yates, 2016; Lengacher et al., 2020; Leow & Chan, 2015a, 2015b; Liljeroos et al., 2014, 2015; Marx et al., 2016; McDonnell et al., 2019; Mehring, 2011; Mitchell, 2013; Mitchell et al., 2014a, 2014b; Northouse et al., 2013; O'Donnell et al., 2014; Parvin et al., 2015; Petzel et al., 2013; Piemonte et al., 2018; Pierce & Steiner, 2015; Pot et al., 2014; Pot, 2016; Poulin et al., 2018; Ramirez-Gomez, et al., 2021; Reeves, 2018; Riegel et al., 2013; Roberge et al., 2016; Robertson & Plueckhahn, 2017; Sanson & Hobbs, 2016; Sarkar et al., 2016b; Schall et al., 2014; Slaboda et al., 2015, 2018; Slosser et al., 2018; Smith et al., 2014; Staley et al., 2018; Steel et al., 2014; Stephen et al., 2012; Stephen, et al., 2010; Stephen, et al., 2010; Stevens et al., 2018; Toledo & Akinyemi, 2017; Velayudhan & Cyster, 2017; Verdonck-De Leeuw et al., 2009; Weber et al., 2010; Werner et al., 2017; Wootten, 2015a, 2015b; Wootten et al., 2014, 2015; Yoon, 2013; Young et al., 2018; Zelinski et al., 2017<br>**Thesis**<br>Chiu, 2008; Ko, 2011; Patterson, 2015; Rinfrette, 2010<br>**Technical note**<br>Zhang et al., 2016<br>**Protocol**<br>Nguyen et al., 2018; Perakis et al., 2009<br>**Commentary**<br>Stephen, et al., 2013<br>**Review**<br>Lobo et al., 2021; Longacre et al., 2018<br>**Trial registry**<br>Blackberry, 2018; Bodschwinna, 2019; Girones, 2017; Ford, 2016; Kursch, 2016; Laver, 2018; Low, 2015; Pot, 2009; Sanson-Fisher, 2016; Singh, 2017<br>**Magazine article**<br>Harvard Health Letter, 2019; Duggleby et al., 2019
Wrong language	Osvath et al., 2017
Duplicate	Kales et al., 2017b; Reeves et al., 2019

References

Agren, S., Evangelista, L. S., Hjelm, C., & Stromberg, A. (2012). Dyads affected by chronic heart failure: a randomized study evaluating effects of education and psychosocial support to patients with heart failure and their partners. *Journal of Cardiac Failure, 18*(5), 359–366. https://doi.org/10.1016/j.cardfail.2012.01.014

Aguado Loi, C. X., Alfonso, M. L., Chan, I., Anderson, K., Tyson, D. D. M., Gonzales, J., & Corvin, J. (2017). Application of mixed-methods design in community-engaged research: lessons learned from an evidence-based intervention for Latinos with chronic illness and minor depression. *Evaluation and Program Planning*, *63*, 29–38. https://doi.org/10.1016/j.evalprogplan.2016.12.010

Alkhushayni, S., & McRoy, S. (2016). mHealth technology: towards a new mobile application for caregivers of the elderly living with multiple chronic conditions (ELMCC). In *DH'16: proceedings of the 6th International Conference on Digital Health Conference,* 11–15. https://doi.org/10.1145/2896338.2896350

Allemann, H., Liljeroos, M., Thylen, I., & Stromberg, A. (2018). Information and Communication Technology (ICT) as a supportive aid; perceptions amongst family caregivers to persons with heart failure. *European Journal of Cardiovascular Nursing*, *17*(1), *S*332. https://doi.org/10.1177/1474515118787764

Applebaum, A., Buda, K., Farberov, M., Teitelbaum, N., Evans, K., Cowens-Alvarado, R., & Cannady, R. (2017). Care for the cancer caregiver: a web-based intervention to address caregiver burden. *Psycho-Oncology*, *26*(1), 54. https://doi.org/10.1002/pon.4354

Applebaum, A., Farberov, M., Teitelbaum, N., Evans, K., Cowens-Alvarado, R., Buda, K., & Cannady, R. (2016). Adaptation of meaning-centered psychotherapy for cancer caregivers (MCP-C) for web-based delivery. *Psycho-Oncology*, *25*(2), 102. https://doi.org/10.1002/pon.4082

Applebaum, A. J., Buda, K. L., Schofield, E., Farberov, M., Teitelbaum, N. D., Evans, K., Cowens-Alvarado, R., & Cannady, R. S. (2018). Exploring the cancer caregiver's journey through web-based meaning-centered psychotherapy. *Psycho-Oncology*, *27*(3), 847–856. https://doi.org/10.1002/pon.4583

Atherton, P. J., Lapid, M. I., Rummans, T. A., Clark, M. M., & Sloan, J. A. (2013). Addressing the needs of cancer patients and caregivers through multi-disciplinary psychosocial interventions. *Supportive Care in Cancer*, *21*(1), S285. https://doi.org/10.1007/s00520-013-1798-3

Bartels, S. L., van Knippenberg, R. J. M., Kohler, S., Ponds, R. W., Myin-Germeys, I., Verhey, F. R. J., & de Vugt, M. E. (2019). The necessity for sustainable intervention effects: lessons-learned from an experience sampling intervention for spousal carers of people with dementia. *Aging & Mental Health*, *24*(12), 2082 - 2093. https://doi.org/10.1080/13607863.2019.1647130

Bateman, D. R., Brady, E., Wilkerson, D., Yi, E. H., Karanam, Y., & Callahan, C. M. (2017). Comparing crowdsourcing and friendsourcing: social media-based feasibility study to support Alzheimer disease caregivers. *JMIR Research Protocols*, *6*(4), e56. https://doi.org/10.2196/resprot.6904

Becker, S. A., & Webbe, F. M. (2008). The Potential of Hand-held assistive technology to improve safety for elder adults aging in place. In: Henriksen K, Battles J.B., Keyes, M.A., et al., editors. Advances in Patient Safety: New Directions and Alternative Approaches (Volume 4: Technology & Medication Safety) (*pp 1 – 16).* Rockville (MD).

Blackberry, I. (2018). VERILY - Virtual Dementia Friendly Rural Communities: using technology to improve support for carers of people living with dementia in rural communities. *Australian New Zealand Trial Registry*, ACTRN12618001213235. https://www.anzctr.org.au/Trial/Registration/TrialReview.aspx?id=374819

Blanton, S., Dunbar, S., & Clark, P. C. (2018). Content validity and satisfaction with a caregiver-integrated web-based rehabilitation intervention for persons with stroke. *Topics in Stroke Rehabilitation*, *25*(3), 168–173. https://doi.org/10.1080/10749357.2017.1419618

Blom, M., & Pot, A. M. (2013). Effectiveness of an internet intervention for family caregivers of people with dementia. *Alzheimer's and Dementia*, *9*(4S Part 2), P882. https://doi.org/10.1016/j.jalz.2013.08.234

Bodschwinna, D. (2019). PartnerCARE - A randomized controlled pilot study to test the feasibility and efficacy of a psycho-oncological online training for partners of patients with cancer. *Deutsche Register Klinischer Studien,* DRKS00017019. https://www.drks.de/drks_web/navigate.do?navigationId=trial.HTML&TRIAL_ID=DRKS00017019

Boele, F.W., Weimer, J.M., Proudfoot, J., Marsland, A.L., Armstrong, T.S., Given, C.W., Drappatz, J.,Donovan, H.S., & Sherwood, P.R. (2021). The effects of SmartCare on neuro-oncology family caregivers' distress: a randomized controlled trial. *Neuro-oncology, 23*(Suppl 2), ii13. https://doi.org/10.1093/neuonc/noab180.042

Boele, F., Sherwood, P., Weimer, J., Proudfoot, J., Marsland, A., Armstrong, T., Given, C., Drappatz, J., & Donovan, H. (2021b) The effects of an online, nurse-led needs-based support program on neuro-oncology family caregivers' distress; a randomised controlled trial. *Neuro-Oncology*, 23(Suppl 6), vi189. https://doi.org/10.1093/neuonc/noab196.749

Boele, F. W., Weimer, J. M., Marsland, A. L., Armstrong, T. S., Given, C. W., Drappatz, J., Donovan, H. S., & Sherwood, P. R. (2022). The effects of SmartCare© on neuro-oncology family caregivers' distress: a randomized controlled trial. *Supportive care in cancer*, 30(3), 2059–2068. https://doi.org/10.1007/s00520-021-06555-5

Bohnak, C. E., & Barron, J. (2017). Workplace help for cancer patients and caregivers. *Journal of the American Geriatrics Society*, *65*(S1), S141–S142. https://doi.org/10.1111/jgs.14915

Bonneux, C., Sankaran, S., Dendale, P., & Coninx, K. (2019). Impact of a tailored e-learning approach during cardiac rehabilitation. *European Journal of Preventive Cardiology*, *26*(1), S9. https://doi.org/10.1177/2047487319860046

Bowman, C., Luck, J., Gale, R. C., Smith, N., York, L. S., & Asch, S. (2015). A qualitative evaluation of web-based cancer care quality improvement toolkit use in the veterans' health administration. *Quality Management in Health Care*, *24*(3), 147–161. https://doi.org/10.1097/QMH.0000000000000063

Breen, A., Lewan, T., Heissenbuttel, A., Hamilton, M., Roleru, P., & Kastle, K. A. (2016). Caregiver efficacy: the cornerstone of outpatient care. *Biology of Blood and Marrow Transplantation*, *22*(*S3*), S449–S450. https://doi.org/10.1016/j.bbmt.2015.11.1027

Bricoli, B. (2015). NICHE develops patient + family app. *Geriatric Nursing*, *36*(3), 249. https://doi.org/10.1016/j.gerinurse.2015.04.010

Brinkert, J.P. (2021) Virtual community-based support proves effective for those impacted by cancer. *Current oncology, 28*(4), 2585. https://doi.org/10.3390/curroncol28040234

Brungardt, A., Marcus, A., Hartley, K., Pearson, S., Fixen, D., Linnebur, S., Parnes, B., & Hildreth, K. (2019). Adaptation and implementation of a dementia care program in an academic geriatric primary care clinic. *Journal of the American Geriatrics Society*, *67*(S1), S283. https://doi.org/10.1111/jgs.15898

Bryant, J., Sanson-Fisher, R., Stevenson, W., Henskens, F., & Smits, R. (2013). Build it, but will they come? Development and patient use of an online information tool designed to reduce psychosocial distress. *Psycho-Oncology*, *22(3)*, 157. https://doi.org/10.1111/j.1099-1611.2013.3394

Burstein, A. A., DaDalt, O., Kramer, B., D'Ambrosio, L. A., & Coughlin, J. F. (2015). Dementia caregivers and technology acceptance: interest outstrips awareness. *Gerontechnology*, *14*(1), 45–56. https://doi.org/10.4017/gt.2015.14.1.005.00

Buzaglo, J., Kennedy, V., Longacre, M., Miller, M., Taylor, J., & Golant, M. (2015). Innovation in developing evidence-based programs that identify and address the social and emotional needs of cancer caregivers: examples of community-initiated research. *Psycho-Oncology*, *24(2)*, S227. https://doi.org/10.1002/pon.3874

Callan, J. A., Siegle, G. J., Abebe, K., Black, B., Martire, L., Schulz, R., Reynolds C., 3rd, & Hall, M. H. (2016). Feasibility of a pocket-PC based cognitive control intervention in dementia spousal caregivers. *Aging & Mental Health*, *20*(6), 575–582. https://doi.org/10.1080/13607863.2015.1031635

Caunca, M. R., Diaz, M. V, De Leon-Benedetti, A., Hartley, G., Czaja, S. J., & Wright, C. B. (2017). Design of a mobile-friendly website to reduce stroke caregiver burden: a focus group study. *Stroke. 48*(1). https://doi.org/10.1161/str.48.suppl_1.tp365

Caunca, M. R., Simonetto, M., Hartley, G., Wright, C. B., & Czaja, S. J. (2018). Design and usability testing of the stroke caregiver support system: a mobile-friendly website to reduce stroke caregiver burden. *Rehabilitation Nursing*, *45*(3), 166 – 177. https://doi.org/10.1097/RNJ.0000000000000196

Chan, J. M., Newton, R. U., Culos-Reed, S. N., Faithfull, S., Lambert, S., Kenfield, S. A., Van Blarigan, E., Lyons, K. S., Ramsdill, J., Zahavich, A., Duncan, L., Hart, N., Dew, M., Moe, E. L., Dixon, C., & Winters-Stone, K. M. (2016). An international, population-level initiative to promote healthy lifestyle practices among prostate cancer survivors. *Journal of Clinical Oncology. Conference*, *34*(3), e287. https://doi.org/10.1200/jco.2016.34.3_suppl.e287

Chien, C. H., Chung, H. J., Liu, K. L., Pang, S. T., Wu, C. T., Chang, Y. H., Huang, X. Y., Chang, Y. H., Lin, T. P., Lin, W. Y., & Chuang, C. K. (2020). Effectiveness of a couple-based psychosocial intervention on patients with prostate cancer and their partners: A quasi-experimental study. *Journal of advanced nursing*, 76(10), 2572–2585. https://doi.org/10.1111/jan.14471

Chiu, T. M., & Eysenbach, G. (2010). Stages of use: consideration, initiation, utilization, and outcomes of an internet-mediated intervention. *BMC Medical Informatics and Decision Making*, *10*(73). https://doi.org/10.1186/1472-6947-10-73

Chiu, T. M. L. (2008). *Usage and non-usage behaviour of E-health services among Chinese Canadians caring for a family member with dementia.* [Doctoral dissertation, University of Toronto] Dissertation Abstracts International: Section B: The Sciences and Engineering, *71*(4), 2286.

Chiu, T., Marziali, E., Colantonio, A., Carswell, A., Gruneir, M., Tang, M., & Eysenbach, G. (2009). Internet-based caregiver support for Chinese Canadians taking care of a family member with Alzheimer disease and related dementia. *Canadian Journal on Aging / La Revue Canadienne Du Viellissement*, *28*(4), 323–336. https://doi.org/10.1017/S0714980809990158

Czaja, S. J., Schulz, R., Perdomo, D., & Nair, S. N. (2014). The feasibility and efficacy of technology-based support groups among family caregivers of persons with dementia. *International Conference on Computers for Handicapped Persons*, *8547*, 455–458. https://doi.org/10.1007/978-3-319-08596-8_71

Czaja, S., Perdomo, D., Nair, S., & Schulz, R. (2011). A technology-based psychoeducational intervention for minority Alzheimer's disease caregivers. *Alzheimer's and Dementia*, *7* (4S), S430–S431. https://doi.org/10.1016/j.jalz.2011.05.1242

Czaja, S., Loewenstein, D., Schulz, R., Sankaran, N.N., & Perdomo, D. (2013). A videophone psychosocial intervention for dementia caregivers. *The American Journal of Geriatric Psychiatry, 21*(11). https://doi.org/10.1016/j.jagp.2013.02.019

Dam, A., Boots, L. M. M., de Vugt, M. E., van Boxtel, M. P. J., & Verhey, F. R. J. (2015). Development of an online social support intervention for people with dementia and their caregivers. *International Psychogeriatrics*, *27*(S1), S160–S161. https://doi.org/10.1017/S1041610215002161

Dam, A. E. H., Christie, H. L., Smeets, C. M. J., van Boxtel, M. P. J., Verhey, F. R. J., & de Vugt, M. E. (2019). Process evaluation of a social support platform 'Inlife' for caregivers of people with dementia. *Internet Interventions*, *15*, 18-27. https://doi.org/10.1016/j.invent.2018.09.002

Davis, B. H., Shehab, M., Shenk, D., & Nies, M. (2015). E-mobile pilot for community-based dementia caregivers identifies desire for security. *Gerontechnology*, *13*(3), 332–336. https://doi.org/10.4017/gt.2015.13.3.003.00

Donovan, H., Boele, F., Weimer, J., Proudfoot, J., Marsland, A., Armstrong, T., Given, C. Drappatz, J., & Sherwood, P. (2021). The effects of a web- and telephone-based intervention (SmartCare) on neuro-oncology family caregivers' distress: a randomized controlled trial. *Supportive care in cancer, 29*(Suppl 1), S212. https://doi.org/10.1007/s00520-021-06285-8

Droes, R. M., van Rijn, A., Rus, E., Dacier, S., Meil, & F. (2019). Utilization, effect, and benefit of the individualized meeting centers support program for people with dementia and caregivers. *Clinical Interventions in Aging*, 2019(14), 1527–1553. https://doi.org/10.2147/CIA.S212852

DuBenske, L. L., Gustafson, D. H., Namkoong, K., Hawkins, R. P., Brown, R. L., McTavish, F., Carmack, C. L., Buss, M. K., Govindan, R., & Cleary, J. F. (2010). Effects of an interactive cancer communication system on lung cancer caregivers' quality of life and negative mood: a randomized clinical trial. *Psycho-Oncology*, 19(2), S100. https://doi.org/10.1002/pon/1776

Duggleby, W., Ghosh, S., Struthers-Montford K., Nekolaichuk, C., Cumming, C., Thomas, R., Tonkin, K., Swindle, J. (2017). Feasibility study of an online intervention to support male spouses of women with breast cancer. *Oncology Nursing Forum*, *44*(6), 765–775. https://doi.org/10.1188/17.ONF.765-775

Duggleby, W., Jovel, K.J., Swindle, J. (2019). Web-based tool strives to reduce dementia caregiver burnout. *Alberta RN*, *74*(4), 30–31.

Duggleby, W., Ploeg, J., McAiney, C., Peacock, S., Fisher, K., Ghosh, S., Markle-Reid, M., Swindle, J., Williams, A., Triscott, J. A. C., Forbes, D., & Ruiz, K. J. (2019). Web-based intervention for family carers of persons with dementia and multiple chronic conditions (My Tools 4 Care): pragmatic randomized controlled trial. *Journal of Medical Internet Research*, *20*(6). https://doi.org/10.2196/10484

Farooqi, A., Danstrom, I., Patel, S., Samaras, A., Flaherty, J., Johnson, K., & Okhravi, H. (2021). Caregiver burden, a virtual group psychoeducation intervention to reduce depression and anxiety in caregivers of those with dementia. *Journal of the American Geriatrics Society, 69*(S1), S307. https://doi.org/10.1111/jgs.17115

Fergus, K. D., McLeod, D., Carter, W., Warner, E., Gardner, S. L., Granek, L., & Cullen, K. I. (2014). Development and pilot testing of an online intervention to support young couples' coping and adjustment to breast cancer. *European Journal of Cancer Care*, *23*(4), 481–492. https://doi.org/10.1111/ecc.12162

Fergus, K., McLeod, D., Carter, W., Gardner, S., Warner, E., Granek, L., & Cullen, K. (2013). An online relationship focused intervention for young couples affected by breast cancer: findings of a feasibility study. *Psycho-Oncology*, *22*(3), 112. https://doi.org/10.1111/j.1099-1611.2013.3393

Fergus, K., Ahmad, S., Gardner, S., Ianakieva, I., McLeod, D., Stephen, J., Carter, W., Periera, A., Warner, E., & Panchaud, J. (2022a). Couplelinks online intervention for young couples facing breast cancer: A randomised controlled trial. *Psycho-oncology*, 31(3), 512–520. https://doi.org/10.1002/pon.5836

Fergus, K., Tanen, A., Ahmad, S., Gardner, S., Warner, E., McLeod, D., Stephen, J., Carter, W., & Periera, A. (2022b). Treatment satisfaction with Couplelinks online intervention to promote dyadic coping in young couples affected by breast cancer. *Fron Psychol*, 13,862555. https://doi.org/10.3389/fpsyg.2022.862555

Ferré-Bergadà, M., Valls, A., Raigal-Aran, L., Lorca-Cabrera, J., Albacar-Riobóo, N., Lluch-Canut, T., & Ferré-Grau, C. (2021). A method to determine a personalized set of online exercises for improving the positive mental health of a caregiver of a chronically ill patient. *BMC medical informatics and decision making*, 21(1), 74. https://doi.org/10.1186/s12911-021-01445-6

Ferré-Grau, C., Raigal-Aran, L., Lorca-Cabrera, J., Lluch-Canut, T., Ferré-Bergadà, M., Lleixá-Fortuño, M., Puig-Llobet, M., Miguel-Ruiz, M. D., & Albacar-Riobóo, N. (2021). A Mobile App-Based Intervention Program for Nonprofessional Caregivers to Promote Positive Mental Health: Randomized Controlled Trial. *JMIR mHealth and uHealth*, 9(1), e21708. https://doi.org/10.2196/21708

Finkel, S., Czaja, S. J., Schulz, R., Martinovich, Z., Harris, C., & Pezzuto, D. (2007). E-Care: a telecommunications technology intervention for family caregivers of dementia patients. The *American Journal of Geriatric Psychiatry*, *15*(5), 443–448. https://doi.org/10.1097/JGP.0b013e3180437d87

Fitzgerald, S. A., Macan Yadrich, D., Werkowitch, M., Piamjariyakul, U., & Smith, C. E. (2011). Creating patient and family education web sites: design and content of the home parenteral nutrition family caregivers web site. *Computers, Informatics, Nursing*, *20*(11), 637–645. https://doi.org/10.1097/NCN.0b013e31822bef7a

Ford, A. (2016). Randomised trial aiming to improve the quality of life of people with dementia (Alzheimer's disease) plus their carers (RAPID-Plus). *Australian New Zealand Clinical Trials Registry,* ACTRN12616000778482. https://anzctr.org.au/Trial/Registration/TrialReview.aspx?ACTRN=12616000778482

Fossey, J., Hales, S., & Clare, W. (2017). Caring for me and you: co-production of an online cognitive behaviour therapy package for carers of people with dementia. *Neurodegenerative Diseases*, *17*(1), 1829.

Fowler, C., Haney, T., & Rutledge, C. M. (2014). An interprofessional virtual healthcare neighborhood for caregivers of elderly with Dementia. *The Journal for Nurse Practitioners*, *10*(10), 829–834. https://doi.org/10.1016/j.nurpra.2014.08.012

Fowler, C. N., Haney, T., & Lemaster, M. (2016). Helping dementia caregivers through technology. *Home Healthcare Now*, *34*(4), 203–209. https://doi.org/10.1097/NHH.0000000000000372

Freddolino, P., Swierenga, S., Woodward, A., Lounds, N., Fitzgerald, J., Coursaris, C., Hughes, A., Fritz, M., & Reeves, M. (2018). Defining the limits of technology-based tools for stroke patients and caregivers. *Stroke, 49*(1), WP303. https://doi.org/10.1161/str.49.suppl_1.wp303

Gaugler, J. E., Reese, M., & Tanler, R. (2016). Care to plan: an online tool that offers tailored support to dementia caregivers. The *Gerontologist*, *56*(6), 1161–1174. https://doi.org/10.1093/geront/gnv150

Gaugler, J. E., Hobday, J.V., Robbins, J.C., & Barclay, M.P. (2015). CARES® ™ Care to plan: an online tool that offers tailored support to dementia caregivers. *Journal of Gerontological Nursing 41*(10), 18–24. https://doi.org/10.3928/00989134-20150804-61

Gies, C. (2011). Developing gender-specific web-based educational modules for caregivers of persons with Alzheimer's disease. *Western Journal of Nursing Research*, *33*(8), 1110–1111. https://doi.org/10.1177/0193945911413675

Girones, X (2017). Evaluation of benefits of the CAREGIVERSPRO-MMD platform giving support and assistance to people living with dementia and their primary caregiver. *ISRCTN Registry*, ISRCTN15654731. https://www.isrctn.com/ISRCTN15654731

Golden, A., Gammonley, D., Powell, G.H., & Wan, T. T. (2017). The Challenges of developing a participatory arts intervention for caregivers of persons with dementia. *Cureus*, *9*(4), e1154. https://doi.org/10.7759/cureus.1154

Grapp, M., Ell, J., Friederich, H., & Maatouk, I. (2020). Development and evaluation of an unguided psychosocial online-intervention for caregivers of cancer patients (OAse): results of a feasibility study. *Oncology research and treatment, 43*(Suppl 1), 149. https://doi.org/10.1159/000506491

Griffiths, P. C., Kovaleva, M., Higgins, M., Langston, A. H., & Hepburn, K. (2018). Tele-savvy: an online program for dementia caregivers. *American Journal of Alzheimer's Disease & Other Dementias*, *33*(5), 269–276. https://doi.org/10.1177/1533317518755331

Gunn, K. M., Turnbull, D., McWha, L., Davies, M., Bidargaddi, N., & Olver, I. (2013). How to successfully connect rural cancer patients and their supporters with supportive care: a culturally acceptable, online South Australian example. *Supportive Care in Cancer*, *21*(1), S297–S298. https://doi.org/10.1007/s00520-013-1798-3

Gunn, K. M., Turnbull, D., McWha, L., Davies, M., & Olver, I. (2012). Connecting rural cancer patients and their families and carers with psychosocial support: the development of a website using a participatory action research framework and behavioural change theory. *Asia-Pacific Journal of Clinical Oncology*, *8*(3), 143. https://doi.org/10.1111/ajco.12029

Halbach, T., Solheim, I., Ytrehus, S., Schulz, T. (2018). A mobile application for supporting dementia relatives: a case study. *Transforming our World Through Design, Diversity and Education*, *256*, 839-846. https://doi.org/10.3233/978-1-61499-923-2-839

Hammer, S., & Klein, L. (2012). Hope network peer support program: developing a psychosocial model of peer support with breast cancer patients and caregivers to decrease emotional distress and social isolation. *Psycho-Oncology*, *21*(1), 16. https://doi.org/10.1111/j.1099-1611.2011.03029_1.x

Han, A., Yuen, H. K., Jenkins, J., & Yun Lee, H. (2021). Acceptance and Commitment Therapy (ACT) Guided Online for Distressed Caregivers of Persons Living with Dementia. *Clinical gerontologist*, 1–12. Advance online publication. https://doi.org/10.1080/07317115.2021.1908475

Han, J., Guo, G., & Hong, L. (2022). Impact of professionally facilitated peer support for family carers of people with dementia in a WeChat virtual community. *Journal of telemedicine and telecare, 28*(1), 68–76. https://doi.org/10.1177/1357633X20910830

Harvard Health Letter (2019). Caregiver nation: new tools to manage a family member's health as well as your own. *44*(10), 1–7.

Hattink, B., Droes, R. M., Sikkes, S., Oostra, E., & Lemstra, A. W. (2016). Evaluation of the digital Alzheimer Center: testing usability and usefulness of an online portal for patients with dementia and their carers. *JMIR Research Protocols*, *5*(3), 193–206. https://doi.org/10.2196/resprot.5040

Heynsbergh, N., Heckel, L., Botti, M., & Livingston, P. M. (2019). A smartphone app to support carers of people living with cancer: a feasibility and usability study. *JMIR Cancer*, *5*(1). https://doi.org/10.2196/11779

Ho, D. W. H., Mak, V., Kwok, T. C. Y., Au, A., & Ho, F. K. Y. (2015). Development of a web-based training program for dementia caregivers in Hong Kong. *Clinical Gerontologist*, *38*(3), 211–223. https://doi.org/10.1080/07317115.2015.1008115

Holroyd-Leduc, J.M., McMillan, J., Jette, N., Brémault-Phillips, S.C., Duggleby, W., Hanson, H.M., & Parmar, J. (2017). Stakeholder meeting: integrated knowledge translation approach to address the caregiver support gap. *Canadian Journal on Aging*, *36*(1), 108–119. https://doi.org/10.1017/S0714980816000660

Jacobs, J. (2020) Managing anxiety in informal caregivers of patients with malignant gliomas: development and refinement of a virtual caregiver-centric intervention. *Psycho-oncology, 29*(S1), 51. https://doi.org/10.1002/pon.5327

Kajiyama, B., Thompson, L. W., Fern, Ez, G., Carter, E. A., Humber, M. B., Day, C. E., & Gallagher-Thompson, D. (2018). Enhancing psychoeducation to cope with dementia caregiving, reduce depressive symptoms and alleviate stress with an online video program: a randomized trial. *Alzheimer's & Dementia*, *14*(7), P1323. https://doi.org/10.1016/j.jalz.2018.06.1889

Kales, H. C. (2017a). WeCareAdvisor: a randomized controlled trial to test an innovative and caregiver-focused tool for the assessment and management of BPSD. *Alzheimer's & Dementia*, *13*(7), 180. https://doi.org/10.1016/j.jalz.2017.07.030

Kales, H. C., Gitlin, L. N., & Lyketsos, C. (2016). “Wecare advisor”: a clinical trial of a caregiver focused, iPad administered algorithm to manage behavioral symptoms. *Alzheimer’s & Dementia*, *12*(7), P217. https://doi.org/10.1016/j.jalz.2016.06.386

Kales, H. C., Gitlin, L. N., Stanislawski, B., Marx, K., Turnwald, M., Watkins, D. C., & Lyketsos, C. G. (2017b). The development of a caregiver-focused, web-based program to assess and manage behavioral and psychological symptoms of dementia. *Alzheimer Disease & Associated Disorders*, *31*(3), 263-270. https://doi.org/10.1097/WAD.0000000000000177

Kales, H. C., Gitlin, L. N., Stanislawski, B., Myra Kim, H., Marx, K., Turnwald, M., Chiang, C., & Lyketsos, C. G. (2018). Effect of the WeCareAdvisor on family caregiver outcomes in dementia: a pilot randomized controlled trial. *BMC Geriatrics*, *18*(113). https://doi.org/10.1186/s12877-018-0801-8

Kishita, N., Gould, R. L., Farquhar, M., Contreras, M., Van Hout, E., Losada, A., Cabrera, I., Hornberger, M., Richmond, E., & McCracken, L. M. (2021). Internet-delivered guided self-help acceptance and commitment therapy for family carers of people with dementia (iACT4CARERS): a feasibility study. *Aging & mental health*, 1–9. Advance online publication. https://doi.org/10.1080/13607863.2021.1985966

Ko, J. W. (2011). *Alzheimer’s disease and related disorders caregiver’s acceptance of a web-based structured written emotional expression intervention.* [Doctoral dissertation, The University of Iowa]. https://doi.org/10.17077/etd.2fqi1los

Koehle, N., Drossaert, C., Schreurs, K., Hagedoorn, M., van Uden-Kraan, C., Verdonck-de Leeuw, I., & Bohlmeijer, E. (2015). User-centered design: development of a web-based self-help intervention for partners of cancer patients. *Psycho-Oncology*, *24*(2), 122-123. https://doi.org/10.1002/pon.3874

Köhle, N., Drossaert, C., Van Uden-Kraan, C., Verdonck-De Leeuw, I., & Bohlmeijer, E. (2018). An online psychological intervention for partners of cancer patients: interest, influencing factors and preferences. *Psycho-Oncology*, *22*(3), 60–61. https://doi.org/10.1111/j.1099-1611.2013.3393

Kovaleva, M., Nocera, J. R., Hepburn, K., Higgins, M., Nash, R., Epps, F., Brewster, G., Bilsborough, E., Blumling, A. A., & Griffiths, P. C. (2022). Attention control in a trial of an online psychoeducational intervention for caregivers. *Research in nursing & health*, 45(3), 337–349. https://doi.org/10.1002/nur.22208

Kubo, A., Hendlish, S., Altschuler, A., Connolly, N., & Avins, A. (2017). Mobile app-based mindfulness intervention for cancer patients and their caregivers-a feasibility study within an integrated health care delivery system. *BMC Complementary and Alternative Medicine*, *17*(1), 317. https://doi.org/10.1186/s12906-017-1783-3

Kubo, A., Kurtovich, E., McGinnis, M., Aghaee, S., Altschuler, A., Quesenberry, C., Jr, Kolevska, T., Liu, R., Greyz-Yusupov, N., & Avins, A. (2020). Pilot pragmatic randomized trial of mHealth mindfulness-based intervention for advanced cancer patients and their informal caregivers. *Psycho-oncology*, 10.1002/pon.5557. Advance online publication. https://doi.org/10.1002/pon.5557

Kursch, A. (2016). RHAPSODY: a pilot study evaluating the usability and benefits of an online learning programme for carers of people with young onset dementia. *Deutsche Register Klinischer Studien,* DRKS00009891. https://www.drks.de/drks_web/navigate.do?navigationId=trial.HTML&TRIAL_ID=DRKS00009891

Kwok, T., Au, A., Wong, B., Ip, I., Mak, V., & Ho, F. (2014). Effectiveness of online cognitive behavioral therapy on family caregivers of people with dementia. *Clinical Interventions in Aging*, *2014*(9), 631–636. https://doi.org/10.2147/CIA.S56337

Lambert, S. D., Duncan, L. R., Ellis, J., Schaffler, J. L., Loban, E., Robinson, J. W., Culos-Reed, N., Matthew, A., Clayberg, K., Santa Mina, D., Goldberg, L., Pollock, P., Tanguay, S., Kassouf, W., Saha-Chaudhuri, P., Peacock, S., & Katz, A. (2020). Acceptability and Usefulness of a Dyadic, Tailored, Web-Based, Psychosocial and Physical Activity Self-Management Program (TEMPO): A Qualitative Study. *Journal of clinical medicine, 9*(10), 3284. https://doi.org/10.3390/jcm9103284

Langbecker, D., & Yates, P. (2016). Development of an online psychoeducational intervention for family caregivers of high-grade primary brain tumour patients. *Psycho-Oncology*, *25*(3), 92. https://doi.org/10.1002/pon.4272

Laver, K. (2018). 'Agents of Change': improving post diagnosis care for people with dementia and their carers through the establishment of a National Quality Collaborative to implement guideline recommendations. *Australian New Zealand Clinical Trials Registry*, ANZCTR12618000268246. https://anzctr.org.au/Trial/Registration/TrialReview.aspx?ACTRN=12618000268246

LeLaurin, J. H., Freytes, I. M., Findley, K. E., Schmitzberger, M. K., Eliazar-Macke, N. D., Orozco, T., & Uphold, C. R. (2021). Feasibility and acceptability of a telephone and web-based stroke caregiver intervention: a pilot randomized controlled trial of the RESCUE intervention. *Clinical rehabilitation*, 35(2), 253–265. https://doi.org/10.1177/0269215520957004

Lengacher, C. Joshi, A., Wittenberg, T., Syed, J., Nieves Bravo, C., Chauca, K., Hamilton, L., Sepehri, F., Reich, R., Dutta, K., Lucas, J., Fonsecca, T., Bornstein, E., & Park, J. (2020). Development of a virtual mindfulness based stress reduction program (vMBSR) for caregivers of advanced stage cancer survivors. *Psycho-oncology, 29*(S1), 67. https://doi.org/10.1002/pon.5328

Leow, M. Q., & Chan, S. W. (2016). Evaluation of a video, telephone follow-ups, and an online forum as components of a psychoeducational intervention for caregivers of persons with advanced cancer. *Palliative Supportive Care*, *14*(5), 474–478. https://doi.org/10.1017/S1478951516000225

Leow, M. Q. H., & Chan, S. W. C. (2015a). A qualitative process evaluation of a psychoeducation intervention for caregivers of a person with advanced cancer at home. *Annals of the Academy of Medicine Singapore*, *44*(10), S235.

Leow, M. Q. H., & Chan, S. W. C. (2015b). Methods of delivering psychoeducation intervention for caregivers of a person with advanced cancer. *Annals of the Academy of Medicine Singapore*, *44*(10), S234.

Li, J., Luo, X., & Li, Q. (2021). An Implementation Process Evaluation Based on an Integrated Psychosocial Support Program of Colorectal Cancer Couples in China: A Pilot Study. *Healthcare (Basel, Switzerland), 9*(2), 110. https://doi.org/10.3390/healthcare9020110

Liljeroos, M., Agren, S., Jaarsma, T., Arestedt, K., & Stromberg, A. (2014). Long term effects of an integrated educational and psychological intervention in prtners to patients affected by heart failure. *European Journal of Cardiovasculr Nursing, 13*(1), S56. https://doi.org/10.1177/1474515114521363

Liljeroos, M., Agren, S., Jaarsma, T., Arestedt, K., & Stromberg, A. (2015). Long term effects of an integrated educational and psychosocial intervention in patient-partner dyads affected by heart failure. *European Heart Journal*, *10*(9). https://doi.org/10.1371/journal.pone.0138058

Lobo, E. H., Frølich, A., Rasmussen, L. J., Livingston, P. M., Grundy, J., Abdelrazek, M., & Kensing, F. (2021). Understanding the Methodological Issues and Solutions in the Research Design of Stroke Caregiving Technology. *Frontiers in public health*, 9, 647249. https://doi.org/10.3389/fpubh.2021.647249

Loh, K. P., Ramsdale, E., Culakova, E., Mendler, J. H., Liesveld, J. L., O'Dwyer, K. M., McHugh, C., Gilles, M., Lloyd, T., Goodman, M., Klepin, H. D., Mustian, K. M., Schnall, R., & Mohile, S. G. (2018). Novel mHealth app to deliver geriatric assessment-driven interventions for older adults with cancer: pilot feasibility and usability study. *JMIR Cancer*, *4*(2). https://doi.org/10.2196/10296

Loi, S.M., Tropea, J., Gaffy, E. et al. (2022) START-online: acceptability and feasibility of an online intervention for carers of people living with dementia. *Pilot Feasibility Stud* 8, 41. https://doi.org/10.1186/s40814-022-00999-0

Longacre, M. L., Applebaum, A. J., Buzaglo, J. S., Miller, M. F., Golant, M., Rowl, H., J., Given, B., Dockham, B., & Northouse, L. (2018). Reducing informal caregiver burden in cancer: evidence-based programs in practice. *Translational Behavioral Medicine*, *8*(2), 145–155. https://doi.org/10.1093/tbm/ibx028

Lorig, K., Thompson-Gallagher, D., Traylor, L., Ritter, P. L., Laurent, D. D., Plant, K., Thompson, L. W., & Hahn, T. J. (2012). Building better caregivers: a pilot online support workshop for family caregivers of cognitively impaired adults. *Journal of Applied Gerontology*, *31*(3), 423–437. https://doi.org/10.1177/0733464810389806

Low, L. (2015). In carers of people with dementia, what is the effect on an online program on managing behaviors common in dementia compared with an education website on levels of carers stress related to behavioural and psychological symptoms? *Australian New Zealand Clinical Trials Registry*, ACTRN126150005509561. http://www.anzctr.org.au/Trial/Registration/TrialReview.aspx?ACTRN=12615000509561

Male, D. A., Fergus, K. D., & Stephen, J. E. (2017). Professional online support group facilitators: guarantors of maximal group utility. *International Journal of Group Psychotherapy*, *67*(3), 314–336. https://doi.org/10.1080/00207284.2016.1240587

Marx, K. A., Gitlin, L. N., Lyketsos, C., Kales, H. C., & Stanislawski, B. (2016). Testing a web-based application to help informal caregivers manage behaviors in persons with dementia: Wecareadvisor™. *Alzheimer's & Dementia*, *12*(7), P300. https://doi.org/10.1016/j.jalz.2016.06.541

McCarron, H. R., Finlay, J. M., Sims, T., Nikzad-Terhune, K., & Gaugler, J. E. (2019). Stakeholder engagement to enhance interventions for family caregivers of people with dementia: a case study of care to plan. *Journal of Gerontological Social Work*, *62*(1), 29–47. https://doi.org/10.1080/01634372.2018.1505797

McDonnell, K. K., Owens, O. L., Beer, J. M., Smith, K., Kennedy, T., Acena, D., & Gallerani, D. (2019). Empowering lung cancer survivors and family members to 'breathe easier': adaptation and evaluation of a m-health intervention. *Journal of Clinical Oncology. Conference*, *37*(15), e23046. https://doi.org/10.1200/JCO.2019.37.15_suppl.e23046

McEvoy, P., Morris, L., Yates-Bolton, N., & Charlesworth, G. (2019). Living with dementia: using mentalization-based understandings to support family carers. *Psychoanalytic Psychotherapy, 33*(4), 233-247. https://doi.org/10.1080/02668734.2019.1709536

McKechnie, V., Barker, C., & Stott, J. (2014). The effectiveness of an internet support forum for carers of people with dementia: a pre-post cohort study. *Journal Medical Internet Research*, *16*(2), e68. https://doi.org/10.2196/jmir.3166

Mehring, S. (2011). Connecting on social networks improves patient and caregiver quality of life. *Pediatric Blood and Cancer*, *57(5)*, 851. https://doi.org/10.1002/pbc.23299

Meichsner, F., Theurer, C., & Wilz, G. (2019). Acceptance and treatment effects of an internet-delivered cognitive-behavioral intervention for family caregivers of people with dementia: a randomized-controlled trial. *Journal of Clinical Psychology*, *75*(4), 594–613. https://doi.org/10.1002/jclp.22739

Meier, A., Lyons, E. J., Frydman, G., Forlenza, M., & Rimer, B. K. (2007). How cancer survivors provide support on cancer-related internet mailing lists. *Journal of Medical Internet Research*, *9*(2), e12. https://doi.org/10.2196/jmir.9.2.e12

Metcalfe, A., Jones, B., Mayer, J., Gage, H., Oyebode, J., Boucault, S., Aloui, S., Schwertel, U., Bohm, M., du Montcel, S., Lebbah, S., De Mendonca, A., De Vugt, M., Graff, C., Jansen, S., Hergueta, T., Dubois, B., & Kurz, A. (2019). Online information and support for carers of people with young-onset dementia: a multi-site randomised controlled pilot study. *International Journal of Geriatric Psychiatry*, *34*(10), 1455–1464. https://doi.org/10.1002/gps.5154

Mitchell, A. J. (2013). Introducing 'Cancer Stories' an innovative video diary programme providing peer support online: development and pilot evaluation. *Psycho-Oncology*, *22*(3), 61–62.

Mitchell, A. J., Shelton, P., Sansone, H., Charlish, J., & Coleman, J. (2014a). Cancerstories© an innovative peer support video diary programme: initial evaluation data from cancer clinicians. *Psycho-Oncology*, *23*(3), 260–261. https://doi.org/10.1111/j.1099-1611.2014.3696

Mitchell, A. J., Shelton, P., Sansone, H., Charlish, J., & Coleman, J. (2014). Cancerstories© an innovative peer support video diary programme: initial evaluation data from 50 patients and families. *Psycho-Oncology*, *23*(3), 107–108. https://doi.org/10.1111/j.1099-1611.2014.3694

Moskowitz, J. T., Cheung, E. O., Snowberg, K. E., Verstaen, A., Merrilees, J., Salsman, J. M., & Dowling, G. A. (2019). Randomized controlled trial of a facilitated online positive emotion regulation intervention for dementia caregivers. *Health Psychology*, *38*(5), 391–402. https://doi.org/10.1037/hea0000680

Murray, E., Kerr, C., Stevenson, F., Gore, C., & Nazareth, I. (2007). Internet interventions can meet the emotional needs of patients and carers managing long-term conditions. *Journal of Telemedicine and Telecare*, *13*(1), S42–S44. https://doi.org/10.1258/135763307781644960

Nguyen, T. A., Nguyen, H., Pham, T., Nguyen, T. H., & Hinton, L. (2018). A cluster randomized controlled trial to test the feasibility and preliminary effectiveness of a family dementia caregiver intervention in Vietnam. *Medicine* 97(42). https://doi.org/10.1097/MD.0000000000012553

Northouse, L., Schafenacher, A., Barr, K., Saunders, E., Yoon, H., Brittain, K., Katapodi, M., & An, L. (2013). Tailored web-based intervention for cancer patients and family caregivers. *Psycho-Oncology*, *22*(2), 7–8. https://doi.org/10.1097/NCC.0000000000000159

Nunez-Naveira, L., Alonso-Bua, B., de Labra, C., Gregersen, R., Maibom, K., Mojs, E., Krawczyk-Wasielewska, A., & Millan-Calenti, J. C. (2016). UnderstAID, an ICT platform to help informal caregivers of people with dementia: a pilot randomized controlled study. *BioMed Research International*, *2016*. https://doi.org/10.1155/2016/5726465

O'Connor, M. F., Arizmendi, B. J., & Kaszniak, A. W. (2014). Virtually supportive: a feasibility pilot study of an online support group for dementia caregivers in a 3D virtual environment. *Journal Aging Studies*, *30*, 87–93. http://doi.org/10.1016/j.jaging.2014.03.001

O'Donnell, J. D., Abernethy, A. P., Samsa, G., Staley, T., MacDermott, K., & Smith, S. (2014). Evaluation of an online, skill-building, group intervention for cancer patients and caregivers: Pillars4Life. *Journal of Clinical Oncology. Conference*, *32*(15), 9573-9573. https://doi.org/10.1200/jco.2014.32.15_suppl.9573

Oliffe, J. L., Han, C. S., Lohan, M., & Bottorff, J. L. (2015). Repackaging prostate cancer support group research findings: an e-KT case study. *American Journal of Men's Health*, *9*(1), 53–63. https://doi.org/10.1177/1557988314528238

Osvath, P., Voros, V., Kovacs, A., Boda-Jorg, A., Fekete, S., Jankovics, R., Tenyi, T., & Fekete, S. (2017). Design and development of a new information technology platform for patients with dementia. *Psychiatria Hungaria*, *32*(4), 437–443.

Pagan-Ortiz, M. E., Cortes, D. E., Rudloff, N., Weitzman, P., & Levkoff, S. (2014). Use of an online community to provide support to caregivers of people with dementia. *Journal Gerontological Social Work*, *57*(6), 694–709. https://doi.org/10.1080/01634372.2014.901998

Papadakos, J., Trang, A., Cyr, A. B., Abdelmutti, N., Giuliani, M. E., Snow, M., McCurdie, T., Pul, Iran, M., Urowitz, S., & Wiljer, D. (2017). Deconstructing cancer patient information seeking in a consumer health library toward developing a virtual information consult for cancer patients and their caregivers: a qualitative, instrumental case study. *JMIR Cancer*, *3*(1), e6. https://doi.org/10.2196/cancer.6933

Parvin, L., Soheilipour, S., Maharaj, N., Miller, A., & Kazanjian, A. (2015). Addressing psychosocial needs of canadian men with prostate cancer and their partners: development of a peer navigation support program. *Psycho-Oncology*, *24*(2), 182–183. https://doi.org/10.1002/pon.3874

Paterson, C., Primeau, C., Pullar, I., Nabi, G. (2019). Development of a prehabilitation multimodal supportive care interventions for men and their partners before radical prostatectomy for localized crostate Cancer. *Cancer Nursing*, *42*(4), E47–E53. https://doi.org/10.1097/NCC.0000000000000618

Patterson, T. R. (2015). *African Americans and the Alzheimer's caregiving experience: differential intervention efficacy within the stress process model of caregiving*. [Doctoral dissertation, North Carolina State University] Dissertation Abstracts International: Section B: The Sciences and Engineering, 77(10).

Perakis, K., Haritou, M., & Koutsouris, D. (2009). ALADDIN, a technology platform for the assisted living of dementia elderly individuals and their carers. In: Omatua S. et al (eds) Distributed Computing, Artificial Intelligence, Bioinformatics, Soft Computing, and Ambient Assisted Living. IWANN 2009 *5518*, 878–881. Springer, Berlin, Heidelberg. https://doi.org/10.1007/978-3-642-02481-8_133

Perales-Puchalt, J., Acosta-Rullán, M., Ramírez-Mantilla, M., Espinoza-Kissell, P., Vidoni, E., Niedens, M., Ellerbeck, E., Hinton, L., Loera, L., Ramírez, A. S., Lara, E., Watts, A., Williams, K., Resendez, J., & Burns, J. (2022a). A Text Messaging Intervention to Support Latinx Family Caregivers of Individuals With Dementia (CuidaTEXT): Development and Usability Study. *JMIR aging*, 5(2), e35625. https://doi.org/10.2196/35625

Perales-Puchalt, J., Ramírez-Mantilla, M., Fracachàn-Cabera, M., Vidoni, E., Ellerbeck, E., Ramírez, A.S., Watts, A., Williams, K., & Burns, J. (2022b). A text message intervention to support Latino dementia family caregivers (CuidaTEXT): Feasibility study. PREPRINT *medRxiv* 2022.04.12.22273809. https://doi.org/10.1101/2022.04.12.22273809

Petzel, S., Vogel, R. I., Chan, D., McClellan, M., Gerber, M., Cragg, J., Jacko, J., & Sainfort, F. (2013). Patient-centered ovarian cancer care: an interactive website to promote emotional quality of life for women and their caregivers. *Psycho-Oncology*, *22*(2), 131. DOI: 10.1111./j.1099-1611.2012.03245

Piemonte, M. E., Dias, C., D'Alencar, M., Ribas, C., Helene, A., & Galves, J. (2018). AMPARO network: A model for education of people living with Parkinson's disease, their care partners and health professionals. *Movement Disorders*, *33*(1), S33–S34.

Pierce, L. L., & Steiner, V. (2015). Qualitative analysis of a nurse's response to stroke caregivers on a web-based supportive intervention. *Stroke Rehabilitation*, *22*(2), 152–159. https://doi.org/10.1179/1074935714Z.0000000011

Pierce, L. L., Steiner, V. L., Khuder, S. A., Govoni, A. L., & Horn, L. J. (2009). The effect of a web-based stroke intervention on carers' well-being and survivors' use of healthcare services. *Disability and Rehabilitation*, *31*(20), 1676–1684. https://doi.org/10.1080/09638280902751972

Piette, J. D., Striplin, D., Marinec, N., Chen, J., & Aikens, J. E. (2015). A randomized trial of mobile health support for heart failure patients and their informal caregivers. *Medical Care*, *53*(8), 692-699. https://doi.org/10.1097/MLR.0000000000000378

Piil, K., Jakobsen, J., Juhler, M., & Jarden, M. (2015). The feasibility of a brain tumour website. *European Journal of Oncology Nursing*, *19*(6), 686–693. https://doi.org/10.1016/j.ejon.2015.05.001

Ploeg, J., McAiney, C., Duggleby, W., Chambers, T., Lam, A., Peacock, S., Fisher, K., Forbes, D. A., Ghosh, S., Markle-Reid, M., Triscott, J., & Williams, A. (2018). A web-based intervention to help caregivers of older adults with dementia and multiple chronic conditions: qualitative study. *JMIR Aging*, *1*(1), e2. https://doi.org/10.2196/aging.8475

Ploeg, J., Northwood, M., Duggleby, W., McAiney, C. A., Chambers, T., Peacock, S., Fisher, K., Ghosh, S., Markle-Reid, M., Swindle, J., Williams, A., & Triscott, J. A. (2019). Caregivers of older adults with dementia and multiple chronic conditions: exploring their experiences with significant changes. *Dementia*, *19*(8), 1–20. https://doi.org/10.1177/1471301219834423

Pot, A., Blom, M., Bosmans, J., Cuijpers, P., & Zarit, S. H. (2014). The effectiveness of 'mastery over dementia', a guided internet intervention to improve caregivers' mental health. *Gerontologist*, *54*, 140.

Pot, A. M. (2016). Innovative technology for people with dementia and their caregivers. *International Psychogeriatrics*, *27*(1), S177. https://doi.org/10.1017/S1041610215002173

Pot, A.M. (2009). Effectiveness of an eHealth intervention on psychological well-being, feelings of burden and perceived health of family caregivers of people with dementia. *Netherlands Trial Register*, NL1934. https://www.trialregister.nl/trial/1934

Pot, A. M., Gallagher-Thompson, D., Xiao, L. D., Willemse, B. M., Rosier, I., Mehta, K. M., I, D., & Dua, T. (2019). iSupport: a WHO global online intervention for informal caregivers of people with dementia. *World Psychiatry*, *18*(3), 365–366. https://doi.org/10.1002/wps.20684

Poulin, V., Carbonneau, H., Provencher, V., Vincent, C., Giroux, D., Rochette, A., Ouellet, M. C., Dawson, D., Nalder, E., & Gagne-Trudel, S. (2018). Promoting leisure participation post-stroke: A web-based program for stroke survivors and their caregivers. *International Journal of Stroke*, *13*(25), 241. https://doi.org/10.1177/1747493018802481

Ramirez-Gomez, L., Johnson, J., Stewart, A.,Meyer, A., Tan, E., Mischoulon, E., Trina, C., & Jain, F. (2021) Feasibility and acceptability of a virtual adaptation of mentalizing imagery therapy for Spanish language family dementia caregivers. *Alzheimer's Dement*, 17(Suppl 7), e051184. https://doi.org/10.1002/alz-051184

Rathnayake, S., Jones, C., Calleja, P., & Moyle, W. (2019). Family carers' perspectives of managing activities of daily living and use of mHealth applications in dementia care: a qualitative study. *Journal of Clinical Nursing*, *28*(23-24), 4460–4470. https://doi.org/10.1111/jocn.15030

Reblin, M., Ketcher, D., Forsyth, P., Mendivil, E., Kane, L., Pok, J., Meyer, M., Wu, Y. P., Agutter, J. (2018a). Feasibility of implementing an electronic social support and resource visualization tool for caregivers in a neuro-oncology clinic. *Supportive Care in Cancer*, *26*(12), 4199–4206. https://doi.org/10.1007/s00520-018-4293-z

Reblin, M., Ketcher, D., Forsyth, P., Mendivil, E., Kane, L., Pok, J., Meyer, M., Wu, Y. P., Agutter, J. (2018b). Outcomes of an electronic social network intervention with neuro-oncology patient family caregivers. *Journal of Neuro-Oncology*, *139*(3), 643–649. https://doi.org/10.1007/s11060-018-2909-2

Reeves, M. (2018). A randomized trial of a social worker led home-based case management to improve outcomes for caregivers of acute stroke patients during the transition period. *European Stroke Journal*, *3*(1), 125. https://doi.org/10.1177/2396987318770127

Reeves, M. J., Fritz, M. C., Woodward, A. T., Hughes, A. K., Coursaris, C. K., Swierenga, S. J., Nasiri, M., & Freddolino, P. P. (2019). Michigan stroke transitions trial. *Circulation: Cardiovascular Quality and Outcomes*, *12*(7). https://doi.org/10.1161/CIRCOUTCOMES.119.005493

Riegel, B., Christiansen, K., Fontana, S., Lillo, E., Lillo, J., Patey, M., Roman, J., Tompkins, V., & Tkacs, N. (2013). Cognitive behavioral therapy by IPAD for caregivers: a pilot study. *Journal of Cardiac Failure*, *19*(8), S40. https://doi.org/10.1016/j.cardfail.2013.06.134

Rinfrette, E. S. (2010). *Impact on informal caregivers of caring for those with early onset dementia.* [Doctoral dissertation, State University of New York]. Dissertation Abstracts International Section A: Humanities and Social Sciences, *71*(7), 2651.

Roberge, M. C., Hughes, S. H., Weimer, J., Misko, S., Armstrong, T., Sherwood, P., & Donovan, H. (2016). Perceived effectiveness of management strategies used by neuro-oncology family caregivers. *Neuro-Oncology*, *18*, vi156.

Robertson, A., & Plueckhahn, T. (2017). Evaluating the use of online chat for the provision of cancer information and support. *Asia-Pacific Journal of Clinical Oncology*, *13*, 204. https://doi.org/10.1111/ajco.12799

Robillard, J. M., Clel, I., Hoey, J., & Nugent, C. (2018). Ethical adoption: a new imperative in the development of technology for dementia. *Alzheimer's Dementia*, *14*(9), 1104–1113. https://doi.org/10.1016/j.jalz.2018.04.012

Ruggiano, N., Brown, E L., Shaw, S., Geldmacher, D., Clarke, P., Hristidis, V., & Bertram, J. (2019). The potential of information technology to navigate caregiving systems: perspectives from dementia caregivers. *Journal of Gerontological Social Work*, *62*(4), 432-450. https://doi.org/10.1080/01634372.2018.1546786

Rutz, M., Gerlach, M., Schmeer, R., Gaugisch, P., Bauer, A., Wolff, D., Behrends, M., Kupka, T., Raudies, S., Meyenburg-Altwarg, I., & Dierks, M. L. (2019). Providing knowledge and support for caring relatives with the smartphone - the MoCaB project. *Pflege*, *32*(6), 1–10. https://doi.org/10.1024/1012-5302/a000695

Sanson-Fisher, R. (2016). A randomized controlled trial of the effectiveness of 'Enable Me': an e-health innovation for stroke survivors and support persons. *Australian New Zealand Clinical Trials Registry*, ACTRN12616001607460. https://anzctr.org.au/Trial/Registration/TrialReview.aspx?ACTRN=12616001607460

Sanson, C. M., & Hobbs, F. (2016). Cancersupport.eu for cancer patients & caregivers. *Psycho-Oncology*, *25*(3), 110. https://doi.org/10.1002/pon.4272

Santin, O., McShane, T., Hudson, P., & Prue, G. (2018). Using a six-step co-design model to develop and test a peer-led web-based resource (PLWR) to support informal carers of cancer patients. *Psycho-Oncology*, *28*(3), 518–524. https://doi.org/10.1002/pon.4969

Sarkar, U., Gourley, G. I., Lyles, C. R., Tieu, L., Clarity, C., Newmark, L., Singh, K., & Bates, D. W. (2016a). Usability of commercially available mobile applications for diverse patients. *Journal of General Internal Medicine*, *31*(12), 1417–1426. https://doi.org/10.1007/s11606-016-3771-6

Sarkar, U., Gourley, G. I., Lyles, C., Tieu, L., Clarity, C., Newmark, L., Singh, K., & Bates, D. W. (2016b). Mobile apps for vulnerable populations study. *Journal of General Internal Medicine*, *1*, S303. https://doi.org/10.1007/s11606-016-3657-7

Schall, A., Kolling, T., Knopf, M., Klein, B., Oswald, F., & Pantel, J. (2014). Intention to use and acceptance of social-emotional robotics in informal and professional elder and dementia care. *Alzheimer's and Dementia*, *4*(10), P766–P767. https://doi.org/10.1016/j.jalz.2014.05.1468

Schaller, S., Marinova-Schmidt, V., Setzer, M., Kondylakis, H., Griebel, L., Sedlmayr, M., Graessel, E., Maler, J. M., Kirn, S., & Kolominsky-Rabas, P. L. (2016). Usefulness of a tailored eHealth service for informal caregivers and professionals in the dementia treatment and care setting: the eHealthMonitor dementia portal. *JMIR Research Protocol*, *5*(2), e47. https://doi.org/10.2196/resprot.4354

Scharett, E., Madathil, K. C., Lopes, S., Rogers, H., Agnisarman, S., Narasimha, S., Ashok, A., & Dye, C. (2017). An investigation of the information sought by caregivers of Alzheimer's patients on online seer Support groups. *Cyberpsychology, Behavior, and Social Networking*, *20*(10), 640–657. https://doi.org/10.1089/cyber.2017.0274

Scott, T. L., Mittelman, M. S., Beattie, E., Parker, D., & Neville, C. (2015). Translating training in the NYU caregiver intervention in Australia: maintaining fidelity and meeting graduate standards in an online continuing professional education setting. *Educational Gerontology*, *41*(10), 710–722. https://doi.org/10.1080/03601277.2015.1048171

Shapiro, C. L., Jacobsen, P. B., Henderson, T., Hurria, A., Nekhlyudov, L., Ng, A., Surbone, A., Mayer, D. K., Rowl, H., J., Shapiro, C. L., Jacobsen, P. B., Henderson, T., Hurria, A., Nekhlyudov, L., Ng, A., Surbone, A., Mayer, D. K., Rowl, & H., J. (2016). ASCO core curriculum for cancer survivorship education. *JCO Oncology Practice*, *12*(2), 145. https://doi.org/10.1200/JOP.2015.009449

Shreve, J., Baier, R. R., Epstein-Lubow, G., & Gardner, R. L. (2016). Dementia caregivers' technology preferences: design insights from qualitative interviews. *Gerontechnology*, *14*(2), 89–96. https://doi.org/10.4017/gt.2016.14.2.004.00

Singh, M. A. F. (2017). HOMeCare: caring for the dementia caregiver and their loved one via the HOMeCare exercise and mindfulness for health program to improve functional capacity and wellbeing. *Australian New Zealand Clinical Trials Registry*, ACTRN12617000347369. https://anzctr.org.au/Trial/Registration/TrialReview.aspx?ACTRN=12617000347369

Slaboda, J., Fail, R., Bowman, B., Wade, A., Morgan, L., & Norman, G. (2018). Focus group findings: Needs of family caregivers of dementia patients. *Journal of Palliative Medicine*, *21*(5), A47. https://doi.org/10.1089/jpm.2018.0153

Slaboda, J., Fail, R., Bowman, B., Wade, A., Morgan, L., Norman, G., & Meier, D. (2015). Focus group findings on needs and supports for family caregivers caring for a family member with cognitive impairment. *Journal of Pain and Symptom Management*, *55*(2), 683–684. https://doi.org/10.1016/j.jpainsymman.2017.12.403

Slosser, A. E., McKibbin, C. L., Carrico, C. P., Longstreth, M. E., Richardson, K. A., & Barry, R. A. (2018). Barriers and priorities for rural and remote dementia caregivers. *Journal of the American Geriatrics Society*, *66*, S321.

Smith, S. K., Abernethy, A. P., Staley, T., MacDermott, K., O'Donnell, J., & Samsa, G. P. (2014). Evaluation of an online, educational group intervention for oncology patients and caregivers: Pillars 4 life. *Psycho-Oncology*, *23*(1), 47. https://doi.org/10.1002/pon.3478

Staley, K., Bergin, B., Parker, S., All, G., Reynolds, H., & Bouverie, J. (2018). Supporting stroke survivors and carers through our stroke recovery service and online stroke support tool-my stroke guide. *International Journal of Stroke*, *13*(25), 193. https://doi.org/10.1177/1747493018789543

Steel, J. L., Kim, K. H., Butterfield, L., Spring, M., Grady, J., Brower, D., Marsh, J. W., Sun, W., Dew, M. A., Antoni, M., & Tsung, A. (2014). Web-based stepped collaborative care intervention in the context of advanced cancer. *Journal of Clinical Oncology*, *32*(15), 9522. https://doi.org/10.1200/jco.2014.32.15_suppl.9522

Steele Gray, C., Miller, D., Kuluski, K., & Cott, C. (2014). Tying eHealth tools to patient needs: exploring the use of eHealth for community-dwelling patients with complex chronic disease and disability. *JMIR Research Protocol*, *3*(4), e67. https://doi.org/10.2196/resprot.3500

Steffen, A. M., & Gant, J. R. (2016). A telehealth behavioral coaching intervention for neurocognitive disorder family carers. *International Journal of Geriatric Psychiatry*, *31*(2), 195–203. https://doi.org/10.1002/gps.4312

Steiner, V., Pierce, L., Drahuschak, S., Nofziger, E., Buchman, D., & Szirony, T. (2008). Emotional support, physical help, and health of caregivers of stroke survivors. *Journal Neuroscientific Nursing*, *40*(1), 48–54.

Stephen, J., Collie, K., McLeod, D., Rojubally, A., Fergus, K., Speca, M., Turner, J., Taylor-Brown, J., Sellick, S., Burrus, K., & Elramly, M. (2014). Talking with text: communication in therapist-led, live chat cancer support groups. *Social Science & Medicine*, *104*, 178–186. https://doi.org/10.1016/j.socscimed.2013.12.001

Stephen, J., Fergus, K., Sellick, S., Speca, M., Taylor-Brown, J., Turner, J., Collie, K., McLeod, D., & Rojubally, A. (2013). When jurisdictional boundaries become barriers to good patient care. *Current Oncology*, *20*(1), 10–13. https://doi.org/10.3747/co.20.1209

Stephen, J., MacGregor, K., Speca, M., Lee, J., Taylor-Brown, J., Doll, R., Kazanjian, A., Collie, K., Turner, J., Fergus, K., McLeod, D., & Fitch, M. (2010). Feasibility and acceptability of a Canadian program of counselor-led online support groups for people affected by cancer: a national cohort study. *Psycho-Oncology*, *19*(2), S284. https://doi.org/10.1002/pon.1776

Stephen, J., Rojubally, A., Macgregor, K., McLeod, D., Speca, M., Taylor-Brown, J., Fergus, K., Collie, K., Turner, J., Sellick, S., & Mackenzie, G. (2013). Evaluation of CancerChatCanada: a program of online support for Canadians affected by cancer. *Current Oncology*, *20*(1), 39–47. https://doi.org/10.3747/co.20.1210

Stephen, J., Rojubally, A., Speca, M., Turner, J., Collie, K., Taylor-Brown, J., Fergus, K., McLeod, D., Giese-Davis, J., & Linden, W. (2012). Benefits of participation in professionally-facilitated online support groups: quantitative and qualitative outcomes for patients, survivors and family caregivers. *Asia-Pacific Journal of Clinical Oncology*, *3*(8), 143.

Stephen, J., Speca, M., Turner, J., Taylor-Brown, J., Collie, K., Fergus, K., McLeod, D., Macgregor, K., & Patkau, T. (2010). Professionally-led online support groups for cancer patients and caregivers: emerging results from a pan-Canadian research initiative. *Psycho-Oncology*, *19*(2), S37–S38. https://doi.org/10.1002/pon.1776

Stevens, E., Barakat, L., Knafl, K., Hobbie, W., Minturn, J., Ginsberg, J., Ogle, S., Noll, R., Ver Hoeve, E., Leri, D., & Deatrick, J. (2018). Development of training in problem solving (TIPS) for caregivers of young adult survivors of childhood brain tumors. *Pediatric Blood and Cancer*, *65*(S2), S425–S426. https://doi.org/10.1002/pbc.27455

Stevenson, W., Bryant, J., Watson, R., Sanson-Fisher, R., Oldmeadow, C., Henskens, F., Brown, C., Ramanathan, S., Tiley, C., Enjeti, A., Guest, J., Tzelepis, F., Paul, C., & D'Este, C. (2020). A multi-center randomized controlled trial to reduce unmet needs, depression, and anxiety among hematological cancer patients and their support persons. *Journal of psychosocial oncology, 38*(3), 272–292. https://doi.org/10.1080/07347332.2019.1692991

Sun, V., Kim, J. Y., Raz, D. J., Chang, W., Erhunmwunsee, L., Uranga, C., Irel, M., A., Reckamp, K., Tiep, B., Hayter, J., Lew, M., Ferrell, B., & McCorkle, R. (2016). Preparing cancer patients and family caregivers for lung surgery: development of a multimedia self-management intervention. *Journal of Cancer Education*, *33*(3), 557–563. https://doi.org/10.1007/s13187-016-1103-5

Taylor, J., & Pagliari, C. (2019). The social dynamics of lung cancer talk on Twitter, Facebook and Macmillan.org.uk. *Npj Digital Medicine*, *2*(51). https://doi.org/10.1038/s41746-019-0124-y

Telke, S., Leininger, B., Hanson, L., & Kreitzer, M. J. (2022). A Randomized Trial of 21 Days of Loving Kindness Meditation for Stress Reduction and Emotional Well-being Within an Online Health Community for Patients, Family, and Friends Experiencing a Cancer Health Journey. *Journal of integrative and complementary medicine*, 28(2), 158–167. https://doi.org/10.1089/jicm.2020.0512

Tixier, M., Gaglio, G., & Lewkowicz, M. (2009). Translating social support practices into online Services for camily Caregivers. In *Proceedings of the ACM 2009 international conference on Supporting group work*, 71–80. https://doi.org/10.1145/1531674.1531685

Toledo, T., & Akinyemi, E. (2017). Caregiver burnout: application of dialectal behavioral therapy. *American Journal of Geriatric Psychiatry*, *25*(3), S88–S89. https://doi.org/10.1016/j.jagp.2017.01.100

Toot, S., Hoe, J., Ledgerd, R., Burnell, K., Devine, M., & Orrell, M. (2012). Causes of crises and appropriate interventions: the views of people with dementia, carers and healthcare professionals. *Aging & Mental Health*, *17*(3), 328–335. https://doi.org/10.1080/13607863.2012.732037

Torp, S., Hanson, E., Hauge, S., Ulstein, I., & Magnusson, L. (2008). A pilot study of how information and communication technology may contribute to health promotion among elderly spousal carers in Norway. *Health Social Care in the Community*, *16*(1), 75–85. https://doi.org/10.1111/j.1365-2524.2007.00725.x

Tyack, C., Camic, P. M., Heron, M. J., & Hulbert, S. (2017). Viewing art on a tablet computer: a well-being intervention for people with dementia and their caregivers. *Journal of Applied Gerontology*, *36*(7), 864–894. https://doi.org/10.1177/0733464815617287

Velayudhan, L., & Cyster, K. (2017). Development and evaluation of mobile application for people with young-onset dementia and their carers. *Alzheimer's and Dementia*, *13*(7), P169. https://doi.org/10.1016/j.jalz.2017.06.2620

Verdonck-De Leeuw, I., Oskam, I., Krebber, A. M., Cuijpers, P., De Bree, R., & Leemans, R. (2009). The need for and use of psychosocial care, peer support, and e-health in head and neck cancer patients and their partners. *Psycho-Oncology*, *18*(2), S259. https://doi.org/10.1002/pon.1594

Weber, M., Ellwood, A. L., Tracy, M., & Wagner, B. (2010). Caring for the family caregivers of people with brain cancer: Laying the groundwork for a caregiver intervention to be delivered across a wide geographic region. *Psycho-Oncology*, *19*(2), S191-S192. https://doi.org/10.1002/pon.1776

Weiss, J. B., Berner, E. S., Johnson, K. B., Giuse, D. A., Murphy, B. A., & Lorenzi, N. M. (2013). Recommendations for the design, implementation and evaluation of social support in online communities, networks, and groups. *Journal of Biomedical Informatics*, *46*(6), 970–976. https://doi.org/10.1016/j.jbi.2013.04.004

Werner, N. E., Gilmore-Bykovskyi, A., Chen, T., Pardell, C., Shenoy, A. V, Zenker, R., & Kind, A. J. (2017). Human factors engineering design of a mobile application to provide informal caregivers with individualized approaches for managing Alzheimer's disease associated behavioral symptoms: a machine learning approach. *Alzheimer's and Dementia*, *13*(7), P839. https://doi.org/10.1016/j.jalz.2017.06.1177

Wilkerson, D. A., Brady, E., Yi, E. H., & Bateman, D. R. (2018). Friendsourcing peer support for Alzheimer's caregivers using Facebook Social Media. *Journal of Technology in Human Services*, *36*(2), 105–124. https://doi.org/10.1080/15228835.2018.1449709

Wittenberg, E., Xu, J., Goldsmith, J., & Mendoza, Y. (2019). Caregiver communication about cancer: development of a mhealth resource to support family caregiver communication burden. *Psycho-Oncology*, *28*(2), 365–371. https://doi.org/10.1002/pon.4950

Wootten, A. (2015a). PROSTMATE: Web-based technology to support men with prostate cancer. *BJU International*, *116*, 56–57.

Wootten, A. (2015b). Using web-enabled technology to support men with prostate cancer: PROSTMATE. *Psycho-Oncology*, *24*(2), 3. https://doi.org/10.1002/pon.3873

Wootten, A., Abbott, J. A., Osborne, D., Austin, D., Klein, B., Murphy, D., & Costello, A. J. (2014). Developing an online psychological support intervention for partners of men with prostate cancer. *Asia-Pacific Journal of Clinical Oncology*, *10*(Suppl 8), 102. https://doi.org/10.1111/ajco.12304

Wootten, A., Abbott, J., Austin, D., Klein, B., Murphy, D., & Costello, A. (2015). Feasibility and pilot testing of an online psychological support intervention for partners of men with prostate cancer. *Psycho-Oncology*, *24*, 37–38.

Yoon, H. (2013). How do cancer patients and caregivers perceive web-based interventions? A qualitative study. *Western Journal of Nursing Research*, *35*(9), 1228–1229. https://doi.org/10.1177/0193945913487172

Young, C., Hamilton-Page, M., Jacques, S., Bromley, R. A., Wong, G., McElhinney, P., Teed, M., & Arthur, G. (2018). Evidence based development of online peer support: co-creating success. *Canadian Journal of Cardiology, 34*(10), S168. https://doi.org/10.1016/j.cjca.2018.07.154

Yousefi, A., Naeimijoo, P., Ghadiany, M., Lighvan, MA., Bakhtiyari, M., & Arani, AM. (2022). A randomized control trial of the effectiveness of online Mindfulness-Based Cancer recovery program on psychological well-being, caregiver burden and resilience in cancer patients' caregivers. Medical Science. 26, ms181e2098. https://doi.org/10.54905/disssi/v26i123/ms181e2098

Yuce, Y. K., & Gulkesen, K. H. (2013). CaregiverNet: A novel social support intervention for locating and securing wandering Alzheimer's patients as soon as possible. In *2013 9th International Wireless Communications and Mobile Computing Conference*, 1405–1411. https://doi.org/10.1109/IWCMC.2013.6583762

Zafeiridi, P., Paulson, K., Dunn, R., Wolverson, E., White, C., Thorpe, J. A., Antomarini, M., Cesaroni, F., Scocchera, F., Landrin-Dutot, I., Malherbe, L., Lingiah, H., Bérard, M., Gironès, X., Quintana, M., Cortés, U., Barrué, C., Cortés, A., Paliokas, I., Votis, K., & Tzovaras, D. (2018). A web-based platform for people with memory problems and their caregivers (CAREGIVERSPRO-MMD): mixed-methods evaluation of usability. *JMIR Formative Research*, *2*(1), e4. https://doi.org/10.2196/formative.9083

Zauszniewski, J. A., Lekhak, N., Napoleon, B., & Morris, D. L. (2016). Resourcefulness training for women dementia caregivers: acceptability and feasibility of two methods. *Issues in Mental Health Nursing*, *37*(4), 249–256. https://doi.org/10.3109/01612840.2015.1119222

Zelinski, E. M., Zak, D. K., & Grossman, M. (2017). Mobile apps for caregivers generally address some but not all forms of evidence-based support. *Alzheimer's and Dementia*, *13*, P162. https://doi.org/10.1016/j.jalz.2017.06.2607

Zhang, M. W., Chan, S., Wynne, O., Jeong, S., Hunter, S., Wilson, A., & Ho, R. C. (2016). Conceptualization of an evidence-based smartphone innovation for caregivers and persons living with dementia. *Technology and Health Care*, *24*(5), 769–773. https://doi.org/10.3233/THC-161165

Zhu, Z., Liu, Y., Che, X., & Chen, X. (2018). Moderating factors influencing adoption of a mobile chronic disease management system in China. *Informatics for Health and Social Care*, *43*(1), 22–41. https://doi.org/10.1080/17538157.2016.1255631
