# Supplementary material for: Implementation of e–Mental Health Interventions for Informal Caregivers of Adults With Chronic Diseases: Mixed Methods Systematic Review With a Qualitative Comparative Analysis and Thematic Synthesis
Source: JMIR Ment Health. 2022 Nov 30;9(11):e41891. doi: 10.2196/41891 (PMC9752475; doi:10.2196/41891)
Supplement: Multimedia Appendix 4 [file mental_v9i11e41891_app4.pdf]

Multimedia Appendix 4

- Table 4.1: Characteristics of included interventions
- Table 4.2: Data table for the qualitative comparative analysis
- Table 4.3: Three condition truth table for the qualitative comparative analysis
- Table 4.4: Two condition truth table for the qualitative comparative analysis
- Figure 4.1: Spider-plots of PRECIS-2 scoring for individual studies
- Figure 4.3: Risk of bias of included randomized controlled trials (n=14)

Table 4.1: Characteristics of included interventions (number of interventions = 29)

Studies & country	Name, aim, intervention theory and/or type of therapy	Duration & frequency	Support	Stakeholder involvement[a]	Main topics	Materials & procedures	Adherence & fidelity
Population: Informal cancer caregivers							
Beer et al, 2020[b]<br><br>USA	Breathe Easier (app)<br><br>Aim: improve the well-being of lung cancer survivors and their informal caregiver<br><br>Mindfulness based stress reduction	Intervention duration: NS<br>Module duration: NS<br>Number of modules: NS<br>Frequency: NS	Type: self-administered<br><br>Provider: NA<br><br>Training: NA<br><br>Mode: NA<br><br>Duration/frequency: NA	Caregivers<br>Care recipients	1) Meditation and mindfulness techniques (e.g. breathing techniques)	Materials:<br>mobile application (under development)<br><br>Procedures:<br>NS	<u>Participant</u><br>Minimum treatment dose: NS<br><br>Usage: NS<br><br><u>Provider</u><br>NA
Bingisser et al, 2018 & Denzinger et al, 2019[c]<br><br>Switzerland	FAMOCA<br><br>Aim: improve psychological well-being in families with a new parental cancer diagnosis<br><br>CBT	Intervention duration: 16 weeks<br>Module duration: NS<br>Number of modules: 4<br>Frequency: 1 module per 4 weeks	Type: minimal<br><br>Provider: psychologist<br><br>Training: NS<br><br>Mode: telephone<br><br>Duration/frequency: 1 feedback session per 4 weeks	Healthcare professionals (adult and child psychologists, oncologists)	1) Understanding what's going on<br>2) Dealing with everyday family life<br>3) Caring for myself and each other<br>4) Planning the future and integrating the past	Materials[d]:<br>Online modules (text, audio, video) with monthly feedback from psychologist; peer discussion forum; Q&A service with psycho-oncologist; video clips of care/family scenarios<br><br>Procedures:<br>Fixed module order, module released every 4 weeks; information tailored (based on age group)	<u>Participant</u><br>Minimum treatment dose: NS<br><br>Usage: 47% completed 4 modules; 20% completed 1-3 modules; 33% completed 1 module[e]<br><br><u>Provider</u><br>NS

Bodschwinna et al, 2022<br><br>Germany	PartnerCARE<br><br>Aim: improve the well-being of partners of people with cancer<br><br>CBT	Intervention duration: 8 weeks<br>Module duration: 30-60 minutes<br>Number of modules: 6 + 1 introduction session + 1 booster + 4 optional<br>Frequency: ~1 module per week (booster 2 weeks after final main module)	Type: minimal<br><br>Provider: psychologists and postgraduate psychology students<br><br>Training: NS<br><br>Mode: online (written)<br><br>Duration/frequency: 1 per week (at the end of each module)	Healthcare professionals (psychologists/ psychiatrists working in oncology)	Introduction<br>1) Specific burdens<br>2) Inner drivers<br>3) Partnership communication<br>4) Handling negative feelings<br>5) Control and acceptance<br>6) Paths and goals<br><br>Optional: 1) Support of own children; 2) Healthy sleep; 3) Closeness and sexuality; 4) Existential burdens	Materials:<br>Online modules (text, audio, visuals) with activities; feedback after each session; caregiving scenarios<br><br>Procedures:<br>Fixed module order; reminders; text messages with motivational messages (optional)	<u>Participant</u><br>Minimum treatment dose: NS<br><br>Usage: 73% completed the intervention (defined as completing 5 main modules)<br><br><u>Provider</u><br>Planned protocol: planned time spent per feedback session was 10 minutes<br><br>Protocol fidelity: Average time spent on feedback was 14 minutes (SD=5.9)

Buss et al, 2008 & DuBenske et al, 2008[b], 2010 & 2014<br><br>USA	CHESS-LC<br><br>Aim: reduce negative mood and burden in lung cancer caregivers<br><br>Stress and coping theory	Intervention duration: 24 weeks[f]<br>Module duration: NA<br>Number of modules: NA<br>Frequency: NA	Type: tailored standardized<br><br>Provider: NA (automated)<br><br>Training: NA<br><br>Mode: NS<br><br>Duration/frequency: NS	Healthcare professionals (clinical psychology, oncologists)<br>IT experts (clinical systems industrial engineering, communications, graphic design and programming)[g]	1) Information about cancer and caregiving<br>2) Emotions and coping strategies<br>3) Decision making tools<br>4) Goal setting	Materials:<br>Online website; peer discussion forum; Q&A service with cancer information specialist; private forum with caregiver's social network; information resources; clinician report (report generated by CHESS for the care recipient's medical care team; includes information such as care recipient's symptoms and questions from caregiver and care recipient); caregiver stories<br><br>Procedures:<br>Materials available at all times; collects data on the user (e.g. needs assessment, keyword searches) to provide better feedback and content suggestions	Participant<br>Minimum treatment dose: NS<br><br>Usage: 73% logged on at least once; 50 minutes of mean use per month[h]<br><br>Provider<br>NA
Carr et al, 2019 & Pensak et al, 2017[b] & 2021<br><br>USA	Pep-Pal (Psychoeducation and Skills-Based Mobilized Intervention)<br><br>Aim: reduce cancer caregiver distress<br><br>Cognitive behavioural stress management	Intervention duration: 12 weeks<br>Module duration: 20 minutes<br>Number of modules: 9 + 1 instructional module on using the intervention<br>Frequency: 1-2 modules per week; watch each module at least once	Type: standardized<br><br>Provider: NS<br><br>Training: NS<br><br>Mode: e-mail<br><br>Duration/frequency: 1 per week	Caregivers<br>Care recipients<br>Healthcare professionals (physicians, nurses, social workers and clinical psychologists)<br>Chaplains	0) Introduction to Pep-Pal<br>1) Introduction to stress management<br>2) Stress and the mind-body connection<br>3) How our thoughts can lead to stress<br>4) Coping with stress<br>5) Strategies for maintaining energy and stamina<br>6) Coping with uncertainty<br>7) managing relationships<br>8) getting the support you need<br>9) Improving intimacy	Materials:<br>Online modules (videos); "Mini-Peps" (short video activities for relaxation, mood or relationship enhancement); general information resources<br><br>Procedures:<br>Modules available at all times; reminders	Participant<br>Minimum treatment dose: NS<br><br>Usage: 39% watched at least 7 of the 9 modules[i]<br><br>Provider<br>NS

Dragomanovich et al, 2021<br><br>USA	Being Present 2.0<br><br>Aim: reduce distress in metastatic gastrointestinal cancer patients and their caregivers<br><br>Mindfulness based stress reduction	Intervention duration: 8 weeks<br>Module duration: 10-30 minutes; 60 minutes for group guided session<br>Number of modules: 40 individual + 8 group guided sessions<br>Frequency: 5 per week; 1 group guided session per week	Type: guided<br><br>Provider: trained meditation teacher<br><br>Training: 20 hours training (viewing recorded example sessions; role playing)<br><br>Mode: group video-call<br><br>Duration/frequency: 1 per week	Caregivers<br>Care recipients	1) Mindful breathing 1 and introduction to mindfulness<br>2) Mindful breathing 2<br>3) Progressive muscle relaxation 1 and guided imagery<br>4) Progressive muscle relaxation 2<br>5) Body awareness meditation<br>6) Attention Awareness Meditation<br>7) Meditation on living when a loved one has cancer and ocean meditation (caregiver specific module)<br>8) Self-guided meditation and loving kindness mediation	Materials:<br>Online recorded meditations (option of male or female voice); live guided group sessions; website with live session recordings<br><br>Procedures:<br>Modules available at all times; reminders; daily text messages with motivational messages	Participant<br>NS<br><br>Provider<br>Planned protocol: group guided sessions planned to have 15 minutes of teaching; 20 minutes of meditation; 20 minutes discussion<br><br>Protocol fidelity: Average time spent on each part of the guided session: teaching: 15 minutes (SD=2.4); meditation: 17.9 minutes (SD=3.1); discussion 8.6 minutes (SD=5.3)

Köhle et al, 2015[j], 2017, 2018[j] & 2021<br><br>the Netherlands	Hold on, for each other<br><br>Aim: reduce psychological distress in caregiving partners of people with cancer<br><br>ACT and self-compassion	Intervention duration: 6-12 weeks<br>Module duration: NS<br>Number of modules: 6 + 2 optional<br>Frequency: NS	Type: minimal or standardized[k]<br><br>Provider: minimal - psychology student (MSc); standardized – NS (automated)<br><br>Training: Yes<br><br>Mode: online (written)<br><br>Duration/frequency: minimal - weekly feedback; standardized - automated message after each module	Caregivers<br>Experts (undefined)	1) Coping with your emotions<br>2) Your resilience plan - how can you keep going?<br>3) My mind works overtime<br>4) What is now really important?<br>5) Afraid, tired and moment of joy<br>6) The art of communication<br><br>Optional: 1) Moving on with life; 2) A good last period	Materials:<br>Online modules (text, audio, activities) with regular personalized or automated feedback; general information resources; peer support (choice of: (1) share response to activities; (2) share tips; (3) privately message each other)<br><br>Procedures:<br>Fixed module order; text messages with short inspirational text (optional)	Participant<br>Minimum treatment dose: NS<br><br>Usage: 69% completed the intervention; average intervention usage: 108 minutes per week[l]<br><br>Provider<br>NS
Kubo et al, 2018[m] & 2019[m]<br><br>USA	Headspace (App)<br><br>Aim: reduce cancer caregiver distress and improve quality of life<br><br>Mindfulness based stress reduction	Intervention duration: 8 weeks<br>Module duration: 10-20 minutes<br>Number of modules: ~ 56<br>Frequency: 7 modules per week	Type: standardized<br><br>Provider: NA (automated)<br><br>Training: NA<br><br>Mode: push notification<br><br>Duration/frequency: scheduled based on participant preference	NS	1) Basics of mindfulness meditation (breathing exercises, body scan, noting and visualization)<br><br>Optional: Targeted programs could be selected based on preference (e.g. anxiety, stress, acceptance)	Materials:<br>Audio sessions; short videos (education on mindfulness)<br><br>Procedures:<br>Modules available at all times; module recommendations provided however caregivers select modules based on need/interest; reminders	Participant<br>Minimum treatment dose: NS<br><br>Usage: 61.5% used intervention for at least 50% of the study period[n]<br><br>Provider<br>NA

Northouse et al, 2014 & Zulman et al, 2012[b]<br><br>USA	Adapted from the family involvement module of FOCUS<br><br>Aim: reduce cancer caregiver distress and improve quality of life<br><br>Stress and coping theory	Intervention duration: 6 weeks<br>Module duration: NS<br>Number of modules: 3<br>Frequency: 1 module per 2 weeks	Type: tailored standardized[o]<br><br>Provider: NA (automated)<br><br>Training: NA<br><br>Mode: online (written)<br><br>Duration/frequency: NS	Caregivers<br>Care recipients<br>Healthcare professionals (nurses trained in original FOCUS program, behavioural scientists)<br>IT experts (web developers, graphic designers)<br>Usability experts	1) Cancer effects on family, value of teamwork, family strengths<br>2) Family's concerns, addressing problems, communication tips<br>3) Different types of support, finding meaning in illness, looking to the future	Materials:<br>Online modules with activities and automated feedback<br><br>Procedures:<br>Content, activities and feedback tailored based on key characteristics (e.g. age, gender, dyad type) and study measures (e.g. self-efficacy)	<u>Participant</u><br>Minimum treatment dose: NS<br><br>Usage: NS<br><br><u>Provider</u><br>NA
Price-Blackshear et al, 2020<br><br>USA	Couples Mindfulness-Based Intervention<br><br>Aim: reduce distress and improve relationship dynamics between young breast cancer survivors and their partners<br><br>Mindfulness based relationship enhancement	Intervention duration: 8 weeks<br>Module duration: 1 hour<br>Number of modules: 8<br>Frequency: 1 module per week	Type: standardized<br><br>Provider: NS<br><br>Training: NS<br><br>Mode: e-mail<br><br>Duration/frequency: 2 per week	NS	1) Introduction to mindfulness<br>2) Meditation, discussion of intervention experiences<br>3) Meditation, pleasant activities calendar, partner yoga<br>4) Meditation, partner activities, unpleasant events calendar<br>5) Meditation, partner activities, stressful communication calendar<br>6) Meditation, partner yoga, discuss stress and self-compassion<br>7) Meditation, partner activities, discuss challenges and support to mindfulness<br>8) Meditation, partner yoga, reflect on experience	Materials:<br>Video modules; audio activities; study manual<br><br>Procedures:<br>Modules released weekly; reminders	<u>Participant</u><br>Minimum treatment dose: NS<br><br>Usage: Of those who completed post-intervention follow-up, 69% report watching all modules<br><br><u>Provider</u><br>NS

Scott & Beatty, 2013<br><br>Australia	Cancer coping online[p]<br><br>Aim: reduce distress among cancer caregivers<br><br>CBT	Intervention duration: 6-7 weeks<br>Module duration: NS<br>Number of modules: 6<br>Frequency: 1 module per week	Type: standardized<br><br>Provider: NS<br><br>Training: NS<br><br>Mode: e-mail<br><br>Duration/frequency: 1 per week	Care recipients<br>Nurses<br>Cancer volunteers (including some who are cancer survivors)	1) Starting treatment<br>2) Coping with physical symptoms and side effects<br>3) Coping with emotional distress<br>4) Body image, identity and sexuality<br>5) Your family and friends<br>6) Completing treatment	Materials:<br>Online modules with activities; general information resources; personal blog; survivor stories<br><br>Procedures:<br>Modules released weekly; reminders; instant quiz feedback	Participant<br>Minimum treatment dose: NS<br><br>Usage: 56% completed all modules; 44% completed 2-5 modules<br><br>Provider<br>NS
Population: Informal dementia caregivers							
Baruah et al, 2020[b], Baruah, Loganathan et al, 2021[b] & Baruah, Varghese et al, 2021<br><br>India	iSupport (adapted for India)<br><br>Aim: reduce dementia caregiver burden and depression<br><br>CBT	Intervention duration: 12 weeks<br>Module duration: NS<br>Number of modules: 23<br>Frequency: NS	Type: standardized<br><br>Provider: NA (automated)<br><br>Training: NA<br><br>Mode: online (written)<br><br>Duration/frequency: automated message after each module	Caregivers<br>Healthcare professionals (nurse, rehabilitation professionals, social workers, psychologists, psychiatrist, geriatrician)	1) Introduction to dementia<br>2) Being a caregiver<br>3) Caring for me<br>4) Providing everyday care<br>5) Dealing with challenging behaviours	Materials:<br>Online modules with activities and automated feedback after module completion; caregiving scenarios<br><br>Procedures:<br>Modules available at all times; caregivers select order of lesson according to needs/interests; instant quiz feedback	Participant<br>Minimum treatment dose: NS<br><br>Usage: 70.3% visited at least once; 40.5% completed at least one module; 16.2% completed at least 5 modules[q]<br><br>Provider<br>NA

Blom et al, 2015 & Pot et al, 2015<br><br>the Netherlands	Mastery over Dementia<br><br>Aim: reduce dementia caregiver depression and anxiety<br><br>CBT	Intervention duration: 24 weeks<br>Module duration: NS<br>Number of modules: 8 + 1 booster<br>Frequency: NS (booster 4 weeks after last module)	Type: minimal<br><br>Provider: psychologist<br><br>Training; none<br><br>Mode: online (written)<br><br>Duration/frequency: psychologist feedback after each module	Caregivers<br>Healthcare professionals (nurses, psychologists, dementia case managers)	1) Coping with behavioural problems<br>2) Arranging help from others<br>3) Time for yourself<br>4) Thinking and feeling<br>5) Not-helping thoughts<br>6) Helping thoughts<br>7) Stand up for yourself: assertiveness<br>8) Communicate problems	Materials:<br>Online modules (text and videos) with activities and feedback<br><br>Procedures:<br>Fixed module order; feedback must be opened to move to next modules; reminders	<u>Participant</u><br>Minimum treatment dose: NS<br><br>Usage: 45.6% completed 8 modules or 8 modules and the booster[r]<br><br><u>Provider</u><br>NS
Boots et al, 2016[b], 2017 & 2018<br><br>the Netherlands & Christie, Schichel et al, 2020, Christie, Boots et al 2020 & Christie et al, 2021<br><br>the Netherlands, Belgium & Germany	Partner in Balance (blended care)<br><br>Aim: improve dementia caregiver self-efficacy and reduce depression<br><br>Stress and coping theory	Intervention duration: 8 weeks<br>Module duration: NS<br>Number of modules: 4 + 2 face-to-face sessions<br>Frequency: 1 module per 2 weeks	Type: guided<br><br>Provider: psychologists and psychiatric nurses<br><br>Training: 2 hours<br><br>Mode: in-person and online (written)<br><br>Frequency: 2 in-person sessions, written feedback after each module	Caregivers<br>Healthcare professionals (psychiatrist, clinical neuropsychologist, health psychologist, occupational therapists, social psychiatric nurses and nurse practitioners)	1) Acceptance<br>2) Balance in activities<br>3) Communication with family members and environment<br>4) Coping with stress<br>5) Focusing on the positive<br>6) Insecurities and rumination<br>7) Self-understanding<br>8) The changing family member<br>9) Social relations and support	Materials:<br>In-person sessions at start and end of intervention; online modules with activities and feedback; peer discussion forum; video clips of caregiving scenarios<br><br>Procedures:<br>Modules available at all times; caregivers select 4 modules based on their needs/interests	<u>Participant</u><br>Minimum treatment dose: NS<br><br>Usage: 87.9% completed all 4 modules[q]<br><br><u>Provider</u><br>Planned protocol: Supervision from experienced professional; record keeping of protocol deviations and contact time with user<br><br>Protocol fidelity: 77% performed according to protocol; 23% reported deviation[s]

Bruinsma, Peetoom, Boots et al, 2021<br><br>The Netherlands	Partner in Balance (adapted for fronto-temporal dementia)<br><br>Aim: improve dementia caregiver self-efficacy and reduce depression<br><br>Stress and coping theory	Intervention duration: 8-10 weeks<br>Module duration: NS<br>Number of modules: 4 + 2 sessions with support provider<br>Frequency: 1 module per 2 weeks	Type: guided<br><br>Provider: psychologists and dementia case managers (specialized nurses or social workers)<br><br>Training: 2 hours<br><br>Mode: session with support provider in-person, via telephone or video-conference, and online (written)<br><br>Frequency: 2 sessions with support provider, written feedback after each module	Caregivers<br>Healthcare professionals<br>Experts	1) Acceptance<br>2) Balance in activities<br>3) Communication<br>4) Focusing on the positive<br>5) Insecurities and rumination<br>6) Self-understanding<br>7) Changes in relative with dementia<br>8) Social relations and support<br>9) Combining care with work<br>10) Impact on family life<br>11) Sexuality and intimacy<br>12) Worries about hereditary	Materials:<br>Sessions with support provided at start and end of intervention; online modules with activities and feedback; peer discussion forum; video clips of caregiving scenarios<br><br>Procedures:<br>Modules available at all times; caregivers select 4 modules based on their needs/interests	<u>Participant</u><br>Minimum treatment dose: NS<br><br>Usage: 74% competed all four modules<br><br><u>Provider</u><br>NS
Bruinsma, Peetoom, Bakker et al, 2021<br><br>The Netherlands	Partner in Balance (adapted for young-onset dementia)<br><br>Aim: improve dementia caregiver self-efficacy and reduce depression<br><br>Stress and coping theory	Intervention duration: 8-10 weeks<br>Module duration: NS<br>Number of modules: 4 + 2 sessions with support provider<br>Frequency: 1 module per 2 weeks	Type: guided<br><br>Provider: psychologists and dementia case managers (specialized nurses or social workers)<br><br>Training: 2 hours<br><br>Mode: session with support provider in-person, via telephone or video-conference, and online (written)<br><br>Frequency: 2 sessions with support provider, written feedback after each module	Caregivers<br>Healthcare professionals (dementia case managers, psychologists, neurologist, clinical geneticist)<br>Researchers	1) Acceptance<br>2) Balance in activities<br>3) Communication<br>4) Focusing on the positive<br>5) Insecurities and rumination<br>6) Self-understanding<br>7) Changes in relative with young-onset dementia<br>8) Social relations and support<br><br>Spouses only option: Sexuality and intimacy<br><br>Other family members only option: Worries about hereditary	Materials:<br>Sessions with support provider at start and end of intervention; online modules with activities and feedback; peer discussion forum; video clips of caregiving scenarios<br><br>Procedures:<br>Modules available at all times; caregivers select 4 modules based on their needs/interests	<u>Participant</u><br>Minimum treatment dose: NS<br><br>Usage: NS<br><br><u>Provider</u><br>Planned protocol: Bi-weekly check-in with lead author; intervision between experienced and less experienced support providers

Contreras et al, 2021 & 2022<br><br>UK	iACT4CARERS<br><br>Aim: improve the mental health of dementia caregivers<br><br>ACT	Intervention duration: 12 weeks<br>Module duration: NS<br>Number of modules: 8<br>Frequency: 1 module per week	Type: minimal<br><br>Provider: minimally trained therapists from the NHS (e.g. assistant psychologist)<br><br>Training: NS<br><br>Mode: online (written)<br><br>Frequency: ~1 per week	Caregivers	1) Intro to ACT<br>2) Values<br>3) Overcoming external barriers<br>4) Overcoming internal barriers (cognitive fusion)<br>5) Overcoming internal barriers (awareness and openness)<br>6) Self-compassion<br>7) Building a pattern of effective action<br>8) Summary and preparing for future	Materials:<br>Online modules (audio, text, video); exercises; written feedback; peer support group via video-conference (optional)<br><br>Procedures:<br>Fixed module order; module released 1 per week	<u>Participant</u><br>Minimum treatment dose: NS<br><br>Usage: 78% completed all modules<br><br><u>Provider</u>[t]<br>Planned protocol: monthly drop-in supervision; feedback to one caregiver per therapist reviewed by two experts<br><br>Fidelity: ACT-inconsistent feedback was none to minimal

Cristancho-Lacroix et al, 2014[b] & 2015<br><br>France	Diapason<br><br>Aim: reduce Alzheimer's disease caregiver stress<br><br>Social cognitive theory & stress and coping theory	Intervention duration: 12 weeks<br>Module duration: 15-30 minutes<br>Number of modules: 12<br>Frequency: 1 module per week	Type: self-administered<br><br>Provider: NA<br><br>Training: NA<br><br>Mode: NA<br><br>Duration/frequency: NA	Caregivers<br>Healthcare professionals (physicians, psychologists, sociologist)<br>Informatics engineer<br>Older adults	1) Caregiver stress<br>2) Understanding the disease<br>3) Maintaining the loved ones' autonomy<br>4) Understanding their reactions - how to recognize behavioural and emotional troubles<br>5) Coping with behavioural and emotional troubles<br>6) Communicating with loved ones<br>7) Improving their daily lives<br>8) Avoiding fall risks<br>9) Pharmacological and non-pharmacological interventions<br>10) Social and financial support<br>11) About the future<br>12) Summary	Materials:<br>Online modules (text, videos); peer discussion forum; caregiver stories<br><br>Procedures:<br>Fixed module order; module must be viewed entirely to open the next	Participant<br>Minimum treatment dose: NS<br><br>Usage: 71% completed at least 10 modules; intervention accessed mean of 19.7 (SD=12.9) times[u]<br><br>Provider<br>NA
Fauth et al, 2022<br><br>USA	Aim: improve dementia caregivers mental health and perceptions of caregiving<br><br>ACT	Intervention duration: 5-6 weeks<br>Module duration: 20-30 minutes<br>Number of modules: 10<br>Frequency: 2 modules per week	Type: self-administered<br><br>Provider: NA<br><br>Training: NA<br><br>Mode: NA<br><br>Duration/frequency: NA	NS	1) Identifying what matters I<br>2) Identifying what matters II<br>3) Noticing avoiding behaviours<br>4) Letting go of avoiding behaviours<br>5) Noticing difficult thoughts<br>6) Noticing difficult thoughts/behaviours<br>7) Everyday mindfulness I<br>8) Everyday mindfulness II<br>9) Committing to your values<br>10) Overview of ACT skills	Materials:<br>Online modules (text; care vignettes); library with online educational resources; printable module summary<br><br>Procedures:<br>Purposeful module order; caregiver selection of phrases from drop-down menus or responses written by participant were feed into future activities within module	Participant<br>Minimum treatment dose: NS<br><br>Usage: NS<br><br>Provider<br>NA

Griffiths et al, 2016, Kovaleva et al, 2019 & Hepburn et al, 2022<br><br>USA	Tele-Savvy (online version of Savvy Caregiver Program)<br><br>Aim: improve dementia caregivers knowledge, skills and perceptions of caregiving<br><br>Social cognitive theory & stress and coping theory	Intervention duration: 7 weeks<br>Module duration: 8-20 minutes short videos; 75-90 minute video-conferences<br>Number of modules: ~ 49 short videos + 7 video-conference sessions<br>Frequency: ~ 1 short video per day; 1 video-call per week	Type: guided<br><br>Provider: NS<br><br>Training: NS<br><br>Mode: online (group video-call)<br><br>Duration/frequency: 1 video-call per week	Caregivers<br>Experts in Savvy Caregiver Program (original face-to-face intervention)	1) Orientation<br>2) Cognitive losses/Caregiver process<br>3) Confusion, caregiver's emotions<br>4) Dementia stages, performance and fit task to talent<br>5) Contented involvement<br>6) Guiding behaviour, decision-making, mastery<br>7) Family systems, program review	Materials:<br>Online modules (video lessons); video clips of caregiving scenarios; workbook with exercises and general information resources<br><br>Procedures:<br>Weekly scheduled video-conferences with other caregivers; other material available at all times	<u>Participant</u><br>Minimum treatment dose: NS<br><br>Usage: NS<br><br><u>Provider</u><br>NS
Gustafson et al, 2019<br><br>USA	D-CHESS<br><br>Aim: fulfill dementia caregiver's needs (psychological, social and practical)<br><br>Stress and coping theory	Intervention duration: 24[f] weeks<br>Module duration: NA<br>Number of modules: NA<br>Frequency: NA	Type: self-administered<br><br>Provider: NA<br><br>Training: NA<br><br>Mode: NA<br><br>Duration/frequency: NA	Caregivers	1) Information about dementia and caregiving<br>2) Emotions and coping strategies<br>3) Decision making tools<br>4) Goal setting	Materials:<br>Online website; peer discussion forum; Q&A service with dementia information specialist; private forum with caregiver's social network; online journal; symptom/well-being monitoring; caregiver stories; general information resources<br><br>Optional: GPS tracking and sensor devices<br><br>Procedures:<br>Materials available at all times	<u>Participant</u><br>Minimum treatment dose: NS<br><br>Usage: 100% logged on during month 1; 64% logged on during month 6<br><br><u>Provider</u><br>NA

Hales & Fossey, 2018[b], Fossey et al, 2021 & Henderson et al, 2022<br><br>England	Caring for me and you<br><br>Aim: reduce dementia caregiver's anxiety, depression, guilt and resentment.<br><br>CBT	Intervention duration: 26 weeks<br>Module duration: 20 minutes<br>Number of modules: 20<br>Frequency: based on caregiver's preference	Type: guided or undetermined[k, v]<br><br>Provider: guided - graduate psychologists trained in online CBT; undetermined - NS<br><br>Training: guided - based on National Health Service Improving Access to Psychological Therapies protocols; undetermined - NS<br><br>Mode: guided - telephone; undetermined - online (written)<br><br>Frequency: guided – 6-10 telephone sessions; undetermined - NS	Caregivers<br>Healthcare professionals (clinical psychologists and psychiatrists)<br>Academic psychologists<br>Current online CBT providers<br>Current user of an online psychological program	1) Introduction: being a caregiver<br>2) Understanding dementia<br>3) Understanding yourself and your reactions<br>4) Understanding more about yourself<br>5) How you think affects how you feel<br>6) Thinking about things differently (emotion 1)<br>7) Checking out your thoughts (emotion 1)<br>8-13) repeat module 6 and 7 for emotions 2-4<br>14) Understanding someone with dementia's needs<br>15) Ways to respond to stress<br>16) How your thinking can affect your interactions<br>17) Being kind to yourself<br>18) Coping with setbacks<br>19) Creating a blueprint<br>20) Continuing your journey	Materials:<br>Online modules (videos) with activities, progress reports and session summaries; caregiving scenarios<br><br>Procedures:<br>Order of module 6 to 13 altered based on caregiver's needs; personalized emails	Participant<br>Minimum treatment dose: NS<br><br>Usage: 26% of participants completed 80% or more of the modules; 46% completed at least 1 module[w]<br><br>Provider<br>Planned: guided - supervision by clinical psychologists; undetermined - NS
Kajiyama et al, 2018<br><br>USA	Mirela (Webnovela)<br><br>Aim: improve coping, and reduce stress and depression in Hispanic dementia caregivers<br><br>CBT	Intervention duration: 4 weeks<br>Module duration: 15-20 minutes<br>Number of modules: 4[x]<br>Frequency: at least 1 module per week	Type: self-administered<br><br>Provider: NA<br><br>Training: NA<br><br>Mode: NA<br><br>Frequency: NA	NS	1) Coping skills<br>2) Managing difficult behaviours<br>3) Self-care<br>4) How to ask for help from social networks	Materials:<br>Online modules (videos with actors enacting care situations)<br><br>Procedures:<br>Modules available at all times	Participant<br>Minimum treatment dose: NS<br><br>Usage: NS<br><br>Provider<br>NA

Kajiyama et al, 2013<br><br>USA	iCare<br><br>Aim: reduce dementia caregiver stress<br><br>CBT	Intervention duration: 12 weeks<br>Module duration: NS<br>Number of modules: 8<br>Frequency: 1 module per 1-1.5 weeks	Type: self-administered<br><br>Provider: NA<br><br>Training: NA<br><br>Mode: NA<br><br>Frequency: NA	Caregivers<br>Professionals (clinical and academic)	1) Information on dementia<br>2) Dealing with stress<br>3) Learning how to relax<br>4) Pleasant activities<br>5) Learning new communication skills<br>6) Managing difficult behaviours<br>7) Healthy habits<br>8) Planning for the future	Materials:<br>Online modules (video); video clips of caregiving scenarios; general information resources; workbook with activities<br><br>Procedures:<br>Fixed module order	Participant<br>Minimum treatment dose: NS<br><br>Usage: NS<br><br>Provider<br>NA
Kozlov et al, 2021<br><br>USA	Mindfulness coach (app)<br><br>Aim: reduce anxiety, stress and improve coping among caregivers of people with dementia or mild cognitive impairment<br><br>Mindfulness based stress reduction	Intervention duration: 8 weeks<br>Module duration: NS<br>Number of modules: 14 levels<br>Frequency: NS	Type: standardized<br><br>Provider: NA (automated)<br><br>Training: NA<br><br>Mode: push notification<br><br>Frequency: based on caregiver's preference	NS	1) Education on mindfulness<br>2) Guided meditation and seated practice	Materials:<br>Audio sessions that get progressively longer<br><br>Procedures: in-person/telephone orientation on using the app; reminders	Participant<br>Minimum treatment dose: NS<br><br>Usage: average 3.5 hours using app; mean level achieved was 6.4 (SD = 4.6); 19% completed all 14 levels<br><br>Provider<br>NA

Sikder et al, 2019<br><br>USA	Mentalizing Imagery Therapy (MIT) app<br><br>Aim: reduce depression and improve mood among dementia caregivers<br><br>Mentalization	Intervention duration: 4 weeks<br>Module duration: NS<br>Number of modules: 4 essays<br>Frequency: 1 module per week and twice daily audio sessions (week 1)/daily audio sessions (week 2-4)	Type: standardized<br><br>Provider: NA (automated)<br><br>Training: NA<br><br>Mode: push notification<br><br>Frequency: 2 per day maximum	NS	1) Five imagery and mindfulness techniques<br>2) Mindfulness tool (mnemonic to quickly de-stress participants)	Materials:<br>Audio sessions and essays (information about mindfulness techniques); caregiver stories (how caregivers used intervention techniques)<br><br>Procedures:<br>Reminders: call to assist with set-up (optional)	Participant<br>Minimum treatment dose: NS<br><br>Usage: mean days accessed: 14 (SD = 10); 29% accessed intervention for at least 19 days (maximum number of days accessed is 28)<br><br>Provider<br>NA
Xiao et al, 2021[b]<br><br>Australia	iSupport (adapted for Australia)<br><br>Aim: improve dementia caregivers' quality of life<br><br>CBT	Intervention duration: NS weeks<br>Module duration: NS<br>Number of modules: 23<br>Frequency: NS	Type: NS<br><br>Provider: NS<br><br>Training: NS<br><br>Mode: NS<br><br>Frequency: NS	Caregivers<br>Healthcare professionals (caregiver support group coordinators, case managers of dementia care services)	1) Introduction to dementia<br>2) Being a caregiver<br>3) Caring for me<br>4) Providing everyday care<br>5) Dealing with challenging behaviours	Materials:<br>Online modules<br><br>Procedures:<br>Caregivers encouraged to select modules based on their needs	Participant<br>NS<br><br>Provider<br>NS

Population: Informal stroke caregivers							
Demers et al, 2022<br><br>USA	Online Langerian Mindfulness<br><br>Aim: improve mental health in stroke survivors and their caregivers<br><br>Langerian mindfulness	Intervention duration: 3 weeks<br>Module duration: 15-30 minutes<br>Number of modules: 15<br>Frequency: at least 5 per week	Type: standardized<br><br>Provider: NA (automated)<br><br>Training: NA<br><br>Mode: online (written)<br><br>Frequency: 1 per week	Care recipient<br>Clinicians<br>Mindfulness experts<br>Researchers	1) Attention to variability<br>2) Positive and negative events<br>3) Unpredictability<br>4) Sense making<br>5) Novelty seeking and novelty producing<br><br>*one module was tailored to caregivers (or care recipients)	Materials:<br>Online modules (text and audio; caregiver module was text only) with activities and information<br><br>Procedures:<br>Modules available at all times; reminders	Participant<br>NS<br><br>Provider<br>NS
Smith et al, 2012<br><br>USA	Aim: reduce depression in female spouses of stroke survivors<br><br>Stress process model	Intervention duration: 11 weeks<br>Module duration: 17 minutes (for videos)<br>Number of modules: 11 videos + 17 video-call sessions<br>Frequency: 1 video per week; 2 video-calls per week	Type: guided<br><br>Provider: nursing PhD student<br><br>Training: NS<br><br>Mode: online (group video-call and written)<br><br>Frequency: 1-2 video-calls per week	Caregivers<br>Rehabilitation specialists	1) Intervention overview<br>2) Getting in touch with your feelings as a caregiver<br>3) Understanding what it's like to be a care recipient<br>4) Being a good listener<br>5) Non-verbal behaviour<br>6) Choice, control & predictability<br>7) Relaxation and positive imagery to control stress<br>8) Pleasant activities<br>9) Saying goodbye	Materials:<br>Online modules (videos) and activities; peer discussion forum; general information resources<br><br>Procedures:<br>Tailored support from provider based on comments within the peer discussion forum and private written communication	Participant<br>Minimum treatment dose: NS<br><br>Usage: 20% non-compliers (attend less than 10 video-call sessions)<br><br>Provider<br>NS

Abbreviations: ACT: acceptance and commitment therapy; CBT: cognitive behavioral therapy; NA: not applicable; NS: not stated

[a]Stakeholder involvement refers to the inclusion of stakeholder feedback during the development of the intervention. Stakeholder feedback could have been collected through research studies, patient and public involvement or if a stakeholder was a member of the research team.

[b]Development/design report

[c]Denzinger et al, 2019 was only included to incorporate feedback collected from participating parent's about the intervention

[d]Intervention description provided refers to the intervention content for parents. The intervention also contained modules tailored to (1) children; and (2) adolescents
[e]Data from Denzinger et al, 2019
[f]Intervention duration was defined as the primary end-point measurement specified within the study (6 months).
[g]Stakeholder involvement only described for the clinician report component of intervention
[h]Data from DuBenske et al, 2014
[i]Data from Pensak et al, 2021
[j]Preliminary reports to inform development
[k]Type of support was under investigation in a randomized controlled trial
[l]Data from Köhle et al, 2021
[m]Reports included both cancer caregivers and cancer survivors. Only data related to cancer caregivers is included
[n]Data from Kubo et al, 2019
[o]Tailored automated refers to support which was automated (e.g. pre-defined feedback messages), however, the message selected to be sent to the participant was tailored based on baseline variables (e.g. self-efficacy)
[p]Intervention originally designed for cancer survivors, however it was used by informal cancer caregivers in this report without adaptation
[q]Data from Baruah et al, 2021
[r]Data from Pot et al, 2015
[s]Data from Boots et al, 2017
[t]Some data was reported in a feasibility study which was not eligible for inclusion: Kishita, N., Gould, R. L., Farquhar, M., Contreras, M., Van Hout, E., Losada, A., Cabrera, I., Hornberger, M., Richmond, E., & McCracken, L. M. (2021). Internet-delivered guided self-help acceptance and commitment therapy for family carers of people with dementia (iACT4CARERS): a feasibility study. Aging & mental health, 1–9. Advance online publication. https://doi.org/10.1080/13607863.2021.1985966
[u]Data from Cristancho-Lacroix et al, 2015
[v]Type of support could not be classified based on available information
[w]Data from Fossey et al, 2020
[x]Pilot report and therefore only tested 4 intervention modules. Full intervention will have 18 modules completed over an 18 week period

Table 4.2: Data table for the qualitative comparative analysis

Study	Conditions					Outcome	
	Peer support	Professional support	Tunneling[a]	Reminders	Tunneling and/or reminders	Hedges' g[b]	Effective intervention set
Baruah et al, 2021	0	0	0	0	0	-0.15	0
Blom et al, 2015	0	1	1	1	1	0.18	0
Bodschwinna et al, 2022	0	1	1	1	1	0.20	0
Boots et al, 2018	1	1	0	0	0	0.29	0
Cristancho-Lacroix et al, 2015	1	0	1	0	1	0.01	0
DuBenske et al, 2014	1	0	0	0	0	0.44	1
Fossey et al, 2021	0	1	0	0	0	0.27	0
Gustafson et al, 2019	1	0	0	0	0	0.13	0
Hepburn et al, 2022	1	1	0	0	0	0.18	0
Kajiyama et al, 2013	0	0	1	0	1	0.09	0
Köhle et al, 2021	1	1	1	0	1	0.35	1
Kubo et al, 2019	0	0	0	1	1	0.50	1
Pensak et al, 2021	0	0	0	1	1	0.32	1
Smith et al, 2012	1	1	0	0	0	0.77	1

[a]Tunneling refers to a controlled module order
[b]Positive values of Hedges' g favour the intervention

Table 4.3: Three condition truth table for the qualitative comparative analysis

Professional support	Peer Support	Tunneling and/or reminders	Number of studies	Membership in the effective intervention set	Raw consistency
0	0	0	1	0	0
1	0	0	1	0	0
0	1	0	2	1	0.5
0	0	1	3	2	0.7
1	1	0	3	1	0.3
1	0	1	2	0	0
0	1	1	1	0	0
1	1	1	1	1	1

Table 4.4: Two condition truth table for the qualitative comparative analysis

Professional support	Tunneling and/or reminders	Number of studies	Membership in the effective intervention set	Raw consistency
0	0	3	1	0.3
1	0	4	1	0.25
0	1	4	2	0.5
1	1	3	1	0.3

Figure 4.1: Spider-plots of PRECIS-2 scoring for individual studies. Trials were scored between 1 (very explanatory) and 5 (very pragmatic) for each domain of PRECIS-2.

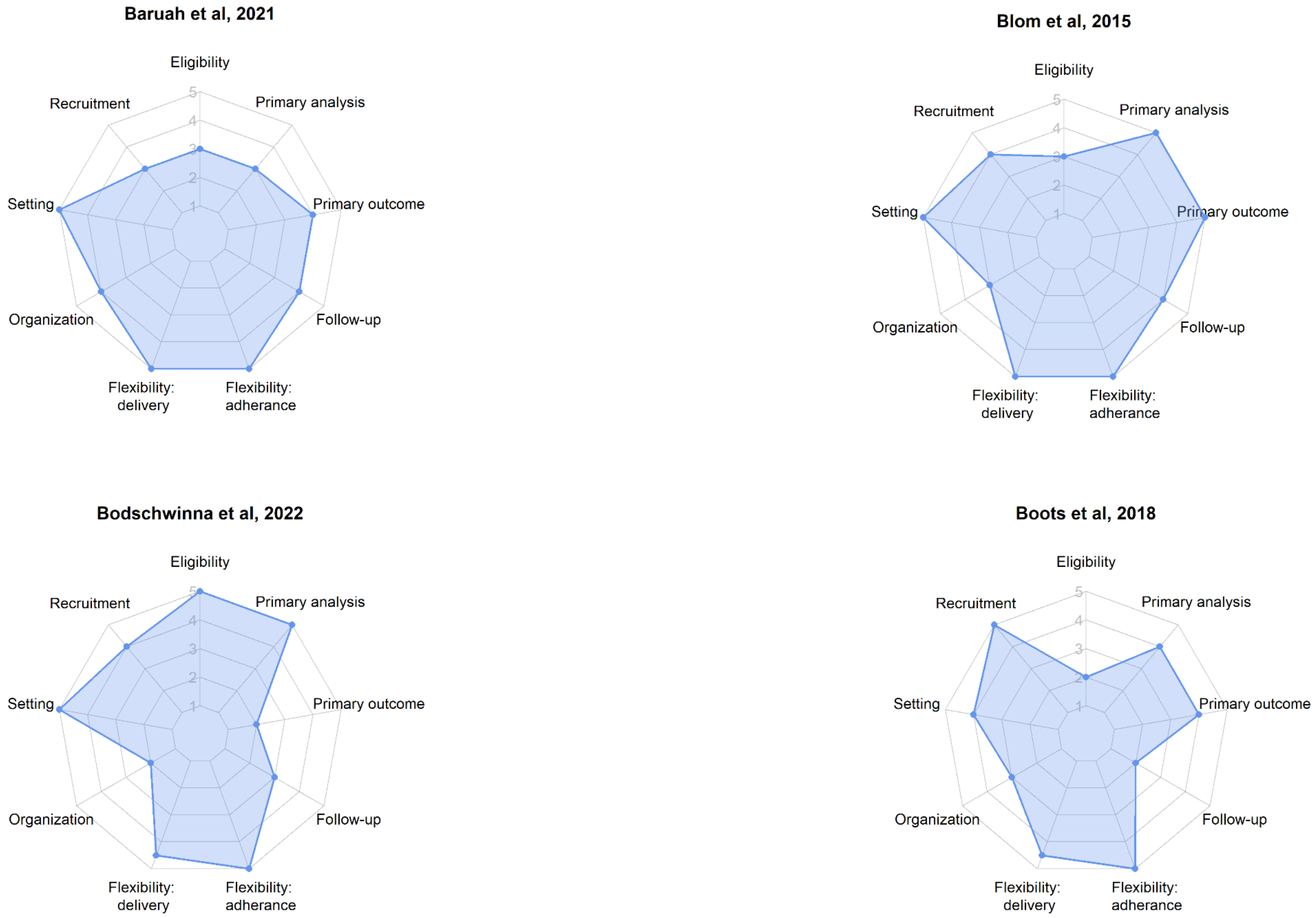

Cristancho-Lacroix et al, 2015

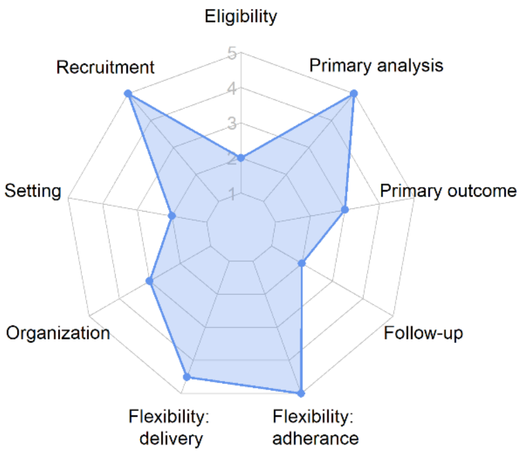

DuBenske et al, 2014

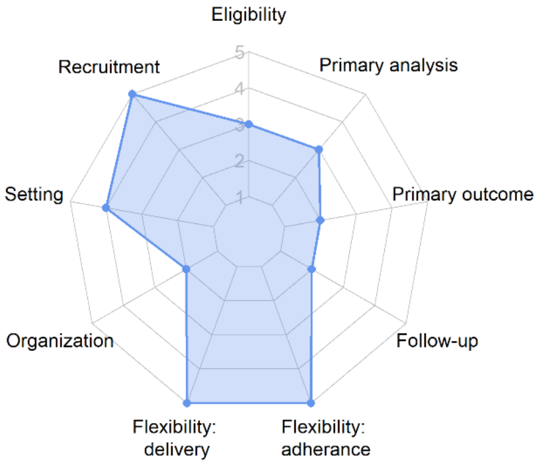

Fossey et al, 2021

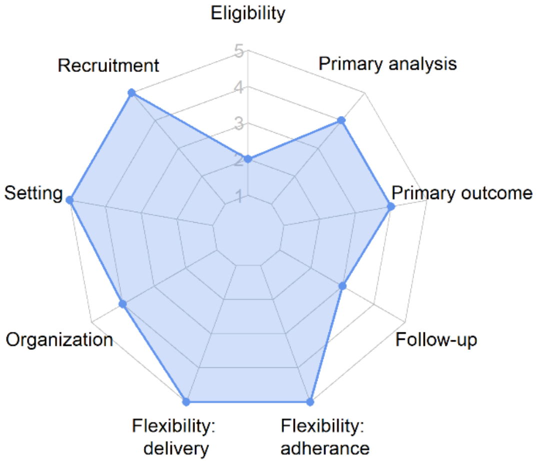

Gustafson et al, 2019

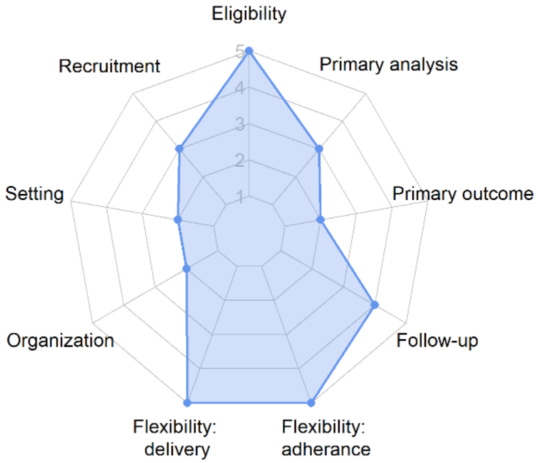

Hepburn et al, 2022

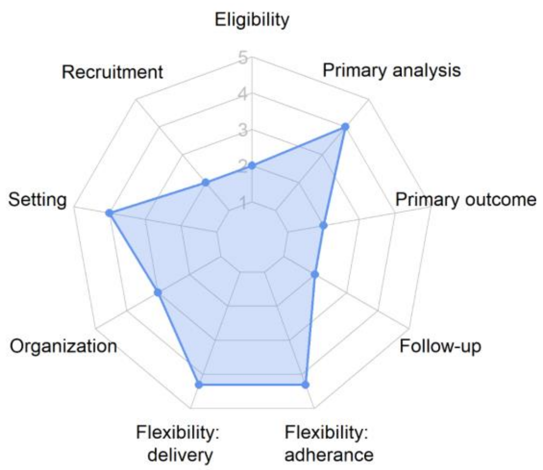

Kajiyama et al, 2013

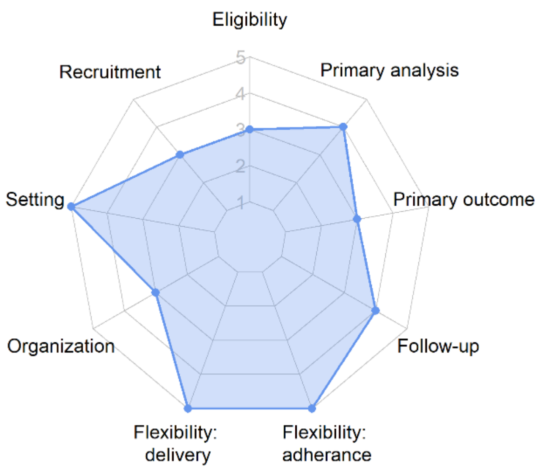

Köhle et al, 2021

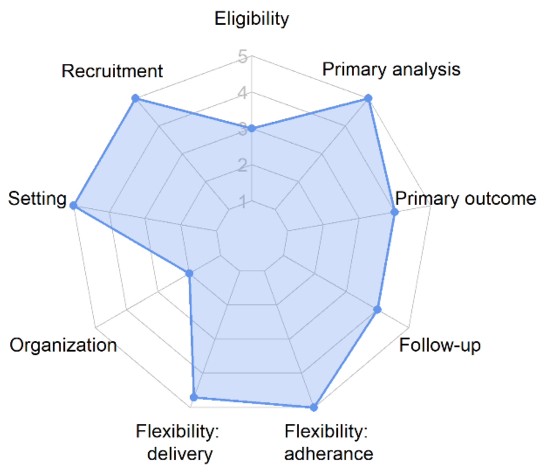

Kubo et al, 2019

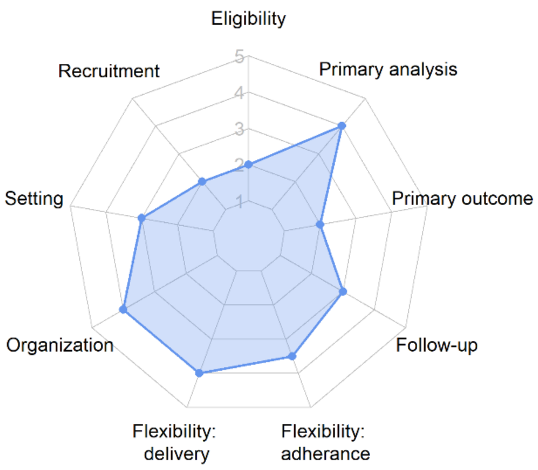

Pensak et al, 2021

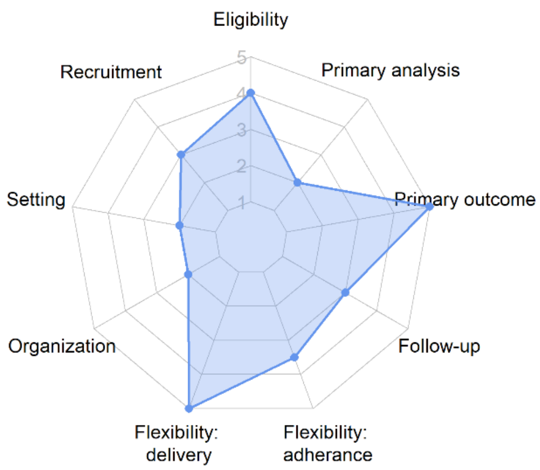

Smith et al, 2012

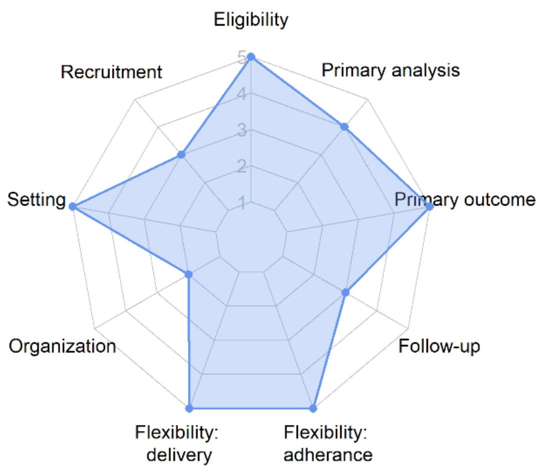

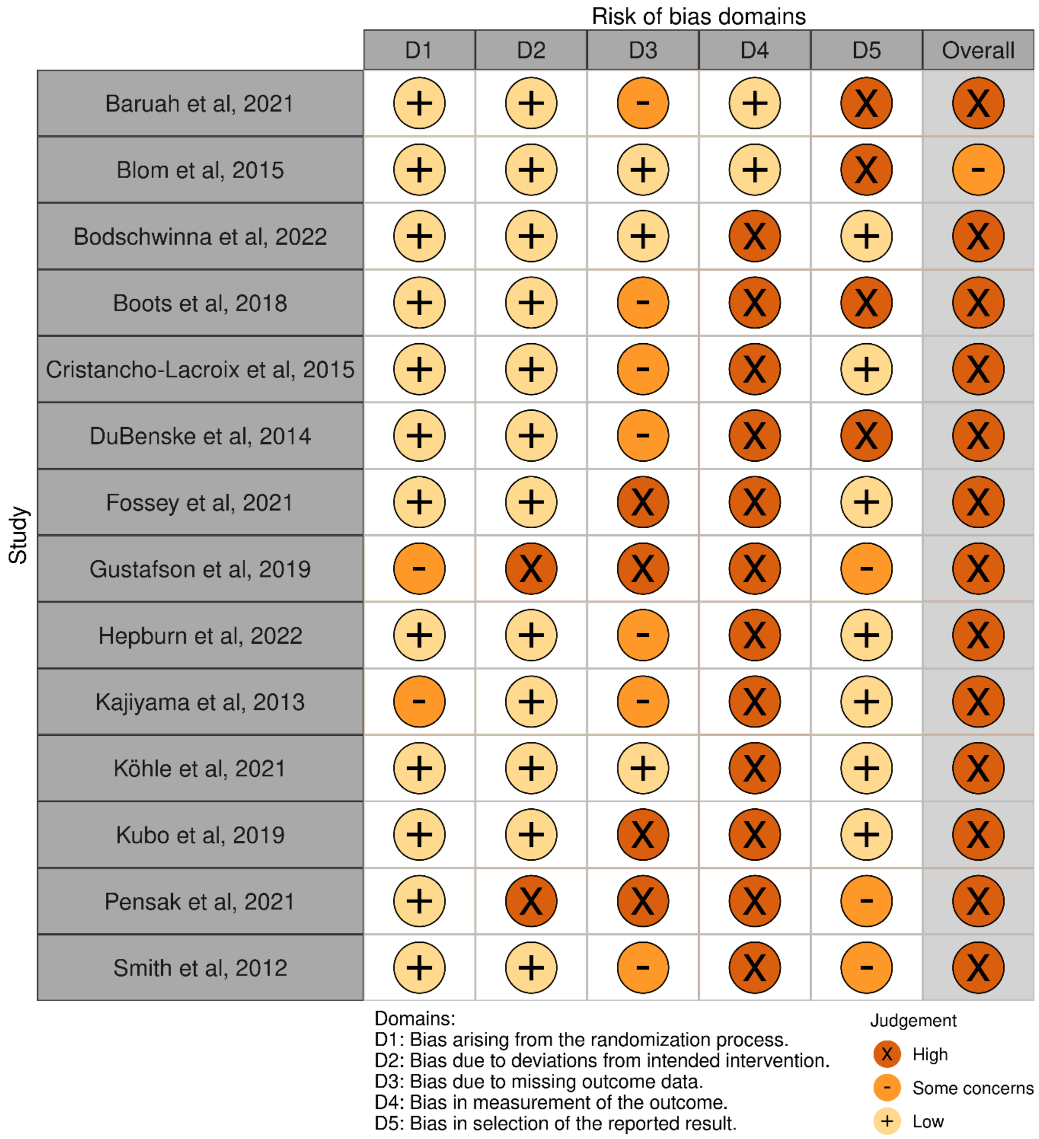

Figure 4.2: Risk of bias of included randomized controlled trials (n=14)
