# Supplementary material for: Implementation of e–Mental Health Interventions for Informal Caregivers of Adults With Chronic Diseases: Mixed Methods Systematic Review With a Qualitative Comparative Analysis and Thematic Synthesis
Source: JMIR Ment Health. 2022 Nov 30;9(11):e41891. doi: 10.2196/41891 (PMC9752475; doi:10.2196/41891)
Supplement: Multimedia Appendix 5 [file mental_v9i11e41891_app5.pdf]

Multimedia Appendix 5

Table 5.1: Identified barriers and facilitators

CFIR Construct	Barrier	Facilitator	Example Quote(s)
**Domain 1: Innovation Characteristics**			
**Innovation Source**	no data	• Trusted/respected innovation source (stakeholders) [1]<br>• Originates from a research project (stakeholders) [2]	"the fact that the intervention was evidence-based and had an academic "name brand recognition" resulting from its origins as a university research project, was a facilitating factor for some municipalities." Author summary from [1] - Facilitator
**Evidence Strength & Quality**	• Lack of pragmatic evidence (stakeholders) [3]<br>• Lack of / hard to measure outcomes relevant for healthcare organizations/decision makers (stakeholders) [1,2]	• Evidence-based practices (caregivers & stakeholders) [1,3,4]<br>• Pragmatic evidence (stakeholders) [2,5]<br>• Knowledge from others with lived experience as evidence (caregivers) [4]	"However, some officials wondered whether these effects would also be obtained outside the research context." Author summary from [3] - Barrier<br><br>"This caregiver also indicated willingness to learn not only evidence-based information but also coping strategies that others employed […]" Author summary from [4] - Facilitator
**Relative Advantage**	• Challenging communication format (e.g. potential for misunderstandings, difficulties with written format) (caregivers & stakeholders) [3,6–8]<br>• Opportunities for distractions (e.g. using mobile phone while using intervention) (caregivers) [4]<br>• Preference for alternative therapy/face-to-face (caregivers & stakeholders) [3,4,6,9–12]	• Convenient and easy to use an online intervention (e.g. no travel, more accessible) (caregivers & stakeholders) [2,4,6,11,13–17]<br>• Easy to express emotions online (caregivers) [18]	"They said that typing sensitive issues on the Partner in Balance platform could be hard for caregivers and coaches, as meanings could be more easily be misconstrued than in face-to-face conversations." Author summary from [3] - Barrier<br><br>"The availability of the information, assignments, and feedback after the intervention was seen as an advantage over mere face-to-face support." Author summary from [13] - Facilitator

**Adaptability**	• Rigid protocols/procedures (caregivers) [6,13,15,19,20]	• Materials available flexibly (e.g. on-demand, based on user-choice) (caregivers & stakeholders) [5,6,9,13,15–18,20–24]<br>• Controlled module order (stakeholders) [13]	"However, four partners were less satisfied with the structure and the fact that they were guided through the intervention, because they felt it was unclear or did not fit their needs. As one of the partners said: "*I noticed during the course that it was difficult to adapt my life and its rhythm to the rhythm of the course. Of course, one does not have exactly those needs in exactly that order… I can imagine it was carefully thought out, but it did sometimes feel like someone was stepping on the brake*."" [20] - Barrier<br><br>"Participants said they appreciated the app's flexibility, specifically that they could do the meditations whenever and wherever they wanted, and that the meditations were short." Author summary from [23] - Facilitator
**Trialability**	no data	• Perceive piloting as valuable (stakeholders) [2]	"[…] as well as the added value of testing interventions through pilots with local collaborations." Author summary from [2] - Facilitator
**Complexity**	• Unfamiliar with technology used (caregivers) [22]<br>• Lack of clear direction within intervention/Complex intervention activities (caregivers & stakeholders) [4–6,13,14,18,25]<br>• Complex implementation activities (stakeholders) [3]	no data	"Also, while Partner in Balance was easy to understand, there were a lot of tasks and organizing involved in making it work (finding coaches, advertising, coordinating, etc), which made it somewhat complex." Author summary from [3] - Barrier

**Design Quality & Packaging**	Content<br>• Lack of tailoring (caregiver demographics, ethnicity, care situation e.g. stage of diagnosis, COVID-19 restrictions) (caregivers & stakeholders) [4,6,9,11–13,15,18,21,26–28]<br>• Idealized and low diversity in caregiving scenarios (caregivers) [4,28]<br>• Lack of materials for providers/stakeholders (stakeholders) [3,8]<br>• Unmet information needs (caregivers & stakeholders) [4–7,9,12,13,16,20,25–27,29–31]<br>• Inappropriate language (too difficult or patronizing tone) (caregivers) [20,24,25]<br>• Inclusion of content/functions that are not helpful or needed (caregivers) [4,6,9,11,18,20,23,28,32]<br>• Need for linguistic and cultural tailoring (caregivers & stakeholders) [26,30]<br><br>Intervention Structure/Components<br>• Lack of personalized support (caregivers) [4,9,12,15,20]<br>• Unmet support needs (caregivers & stakeholders) [2,5,7,9,12–15,17,20,28]<br>• Unclear purpose of intervention functions (caregivers) [4,6]<br>• Confusing or unappealing structure (caregivers & stakeholders) [4–6,20,25,27,30,31]<br>• Negative tone or appearance (caregivers & stakeholders) [5,6,9,13,20,25]<br>• Limited time to access intervention (caregivers) [4,5,15]<br>• Too demanding (caregivers) [26,27,32–34]<br>• Lack of two-way communication between provider and caregiver (stakeholders) [8]	Content<br>• Focus on positive (caregivers & stakeholders) [5,9,13,20]<br>• Useful and practical content (caregivers & stakeholders) [2–7,9,11–16,18–23,25–32,34,37–39]<br>• Tailored content (caregivers & stakeholders) [6,9,20,21,26,30]<br>• Contact with and/or information delivered from other caregivers (via peer forum, group session or caregiving vignettes) (caregivers & stakeholders) [4,6,9,13,15–18,20,21,26,30]<br><br>Intervention Structure/Components<br>• Intervention fully or partially independent (e.g. not dyadic) (caregivers) [9,16,27]<br>• Dyadic format (caregivers) [5,9,19,27,29]<br>• Manages individual needs within dyadic format (stakeholders) [7]<br>• Easy to use/well designed (caregivers & stakeholders) [5,6,8,13–15,18–25,28–31,36,39–41]<br>• Easy to understand language (stakeholders & caregivers) [9,25,26,34]<br>• Incorporation of professional support (caregivers & stakeholders) [4,6,9,13,15,16,18,20,21,25–27,30,32,39]<br>• Anonymity (caregivers) [9,13,16,20]<br>• Ability to track intervention usage (stakeholders) [3]<br>• Not too time-consuming (caregivers) [5,9,15–17,23,31,33]<br>• Incorporation of techniques to support engagement (e.g. homework, progress bars, reminders) (caregivers) [14,24,26,31,34]	Content<br>"*Just the actress, Nancy, it was just like, oh yes, I've had that same problem at home... in real life, that's not how it is. She makes it seem so simple. Like, I went out with my friends, we had a great time. I feel much better. That's not how life is."* Participant quote from [28] - Barrier<br><br>"Also, participants mentioned that the intervention should have a positive approach. According to them, thinking positively and accentuating what still can be done, instead of what no longer can be done, is a source of hope and energy for both the partner and the cancer patient […]" Author summary from [9] – Facilitator<br><br>Intervention Structure/Components<br><br>"They expected more specific and individualized advice, and more "human interaction" with professionals or peers." Author summary from [12] – Barrier<br><br>"[…] a few caregivers commented that it was "user friendly" and several mentioned that the notifications provided "a good reminder" to use the app." Author summary from [14] – Facilitator

	• Inclusion of patients inhibits caregivers ability to express themselves (caregivers) [32]<br><br><u>Delivery format</u><br>• Limited viewing options (e.g. internet dependent, lack of audio/video material) (caregivers & stakeholders) [5–7,13,20,24,26,28,35,36]<br>• Animated delivery format undervalues the information (caregivers) [5]<br>• Technical difficulties (e.g. connectivity issues) (caregivers & stakeholders) [1,2,4,6,23,26,28,31,35]	<u>Delivery format</u><br>• Use of different media (e.g. audio, video) (caregivers & stakeholders) [13,17,20,26]<br>• Face-to-face component (caregivers & stakeholders) [8,13,25]<br>• Usage across devices (e.g. laptop, smartphone) (caregivers & stakeholders) [18,26]	<u>Delivery format</u><br><br>"*I think having it [the program] on an Internet interface would be the really appropriate way to go, but there might be situations where Internet access isn't that available. You might think about having a separate option where you could download it.*" Participant quote from [5] – Barrier<br><br>"A mobile application or a web platform compatible with mobile phones, to enhance the usability of the program and make it more appealing to the younger generation was suggested by the caregivers and professionals alike." Author summary from [26] – Facilitator
**Cost**	• Unclear costs (stakeholders) [1]<br>• Belief intervention unlikely to be cost-effective (stakeholders) [3,42]	• Perceive intervention as providing value for money (stakeholders) [2]<br>• Perceive as low cost (caregivers) [30]	"This municipality felt that the inability of Partner in Balance to guarantee what a license would cost after the project's end was a significant barrier." Author summary from [1] – Barrier<br><br>"Good investment" Author summary from [2] - Facilitator

Domain 2: Outer Setting			
**Needs & Resources of Those Served by the Organization**	• Low population interest in eHealth (stakeholders) [1]<br>• Unsure what caregiver needs/preferences are (stakeholders) [1,3]	• Topics suit caregiver needs (stakeholders) [2,3]	"Some respondents felt that eHealth is mainly pushed through top-down initiatives but that the population of their municipality does not express a desire for it." Author summary from [1] - Barrier<br><br>"The first category of this theme "good content" showed that all groups of stakeholders had positive attitudes toward the Partner in Balance content and thought many of its components were useful and timely. The second category refers to how stakeholders (especially policy makers and health care professionals) thought that Partner in Balance met caregiver needs, [...]."Author summary from [2] - Facilitator
**Cosmopolitanism**	• Lack of time among organizations (stakeholders) [1]	• Approval/collaboration between multiple organizations (stakeholders) [1–3,30]	*"[...] the cooperating partners had full agendas [...]"* Participant quote from [1] - Barrier<br><br>"Regarding how the implementing organizations are linked to other organizations, respondents stated that the interventions needed to be offered through an external party (not through the municipality) and cooperation with care providers would always be necessary, as they would have to agree to execute the interventions. Some municipalities reported that the SFC project had been a good chance to connect and strengthen their local dementia care networks." Author summary from [3] - Facilitator
**Peer Pressure**	no data	• Digitalization in other sectors (stakeholders) [1]	"*All over the community it's the digital things that are successful and also the future and so, it would be strange if the medical part doesn't take part*." Participant quote from [1] - Facilitator

**External Policy & Incentives**	no data	• Fit between intervention and wider policy (stakeholders) [2,3]	"*Yes, I think it fits within the policy yes. It fits within the informal care policy, is increasingly in line with the policy of health insurers, who say if we support informal carers then it will yield results. Also for the informal caregiver and the person they care for, so that they stay better in balance, can last longer, so I think it fits within the policy*." Participant quote from [3] - Facilitator
**Domain 3: Inner Setting**			
**Structural Characteristics**	no data	no data	
**Networks & Communications**	• Lack of internal network to support implementation (stakeholders) [2,3]	• Open communication channels within implementation team (stakeholders) [3]	"The implementation of the eHealth interventions was usually the sole responsibility of one person within the municipality. Municipality officials stressed that this was not enough, and that there should be a team to tackle the implementation together." Author summary from [3] - Barrier<br><br>"Municipalities added that it was easy to set up the necessary meetings with the Partner in Balance team." Author summary from [3] - Facilitator
**Culture**	no data	no data	
**Implementation Climate**	no data	• Lack of alternative supports available for caregivers (caregivers & stakeholders) [26]	"The consensus from all the three [focus group discussions] overall was that the existence of such an online program in itself was very encouraging considering the dearth of existing resource" Author summary from [26] - Facilitator
**a. Tension for Change**	no data	no data	

**b. Compatibility**	• Mismatch between organizations clients and intervention target population (stakeholders) [3,13]<br>• Unfamiliar intervention approach (stakeholders) [7,13,43]<br>• Caregiver support in patient oriented systems (stakeholders) [2,13]<br>• Lack of integration within existing systems (e.g. electronic medical records)(stakeholders) [7,43]<br>• Overlap in function with existing systems (stakeholders) [7]<br>• Perceived low digital literacy among implementers (stakeholders) [3]	• Ability to integrate intervention within workflow (stakeholders) [3,8,13,30]<br>• Flexible usage options for providers (e.g. ways of providing feedback, provider specific settings) (stakeholders) [13,43]<br>• Aligns with organizational priorities and goals (stakeholders) [1,3]	"Coaches mentioned that elderly care organizations in the Netherlands often file caregiver support under patient care, which could create problems for the implementation of caregiver support programs when the person with dementia is not registered." Author summary from [13] - Barrier<br><br>"Municipality officials mentioned that their choice of intervention depended on whether the intervention was in line with the values and policy of the municipality. In this regard, they mentioned that Myinlife and/or Partner in Balance matched their work on sustainability, caregiver support, and "staying close to the citizen."" Author summary from [1] - Facilitator
**c. Relative Priority**	• Organization already involved in other interventions for caregivers (stakeholders) [13]<br>• Low priority (stakeholders) [3]	no data	"Other barriers included involvement in other caregiver support approaches." Author summary from [13] - Barrier
**d. Organizational Incentives & Rewards**	• Lack of incentives (stakeholders) [3]	no data	"[…] interventions had low relative priority […]."Author summary from [3] – Barrier
**e. Goals & Feedback**	• Lack of goal setting (stakeholders) [3]	• Ability to monitor intervention usage (stakeholders) [3]	"For both interventions, there was not enough goal setting and feedback […]."Author summary from [3] – Barrier<br><br>"As management is primarily interested in concrete output, it is important to keep track of the output and use of the interventions." Author summary from [3] - Facilitator
**f. Learning Climate**	no data	• Multiple modes and opportunities for support from supervisors and colleagues (stakeholders) [8]	"*I think it was really good and also the fact that we had so many options to attend the supervision meetings and on different days and different times. I think that was really*

			good because you kind of feel supported […] if you are struggling with some participants it's good that you have that instance where you can talk to someone else and ask their opinion and so yes. I think that having that was really helpful." Participant quote from [8]- Barrier
**Readiness for Implementation**	• Upcoming organizational restructuring (stakeholders) [1,13]	no data	"For instance, politically, imminent elections and the merging of three municipalities into one municipality made concrete planning difficult, as the budget and officials responsible might change." Author summary from [1] - Barrier
**a. Leadership Engagement**	• Low engagement of leadership (stakeholders) [1,3,13]	no data	"*I introduced this. My supervisor, yes, but I work in my department alone. .... We have not really discussed it with anyone else. So, my supervisor is not actively pushing this now either*." Participant quote from [3] - Barrier
**b. Available Resources**	• Lack of resources (stakeholders) [1,3,13]	no data	"[…] municipality officials stressed a lack of resources on the coaches' side, such as time and money, as a foreseeable barrier to effective dissemination and subsequently, implementation." Author summary from [1] - Barrier
**c. Access to Knowledge & Information**	• Unclear clinical guidelines for handling information collected from intervention (stakeholders) [7,43]<br>• Lack of time/support to become familiar with intervention (stakeholders) [8,13]	• Access to training and support materials (stakeholders) [8]	"Clinicians raised a second, related issue: whether data provided by the caregiver alone should be included as part of the patient's medical record." Author summary from [7] – Barrier<br><br>"*However, [Project Chief Investigator] provided a Word document which had some of the ideas that we had during the training, so some ACT consistent feedback that we discussed. So, I referred back to that if I was ever stuck and then again if I was unsure as*

			to how to give feedback or what to give.” Participant quote from [8] - Facilitator
**Domain 4: Individual Characteristics**			
**Knowledge & Beliefs about the Innovation**	• Perceive online interventions as impersonal (caregivers & stakeholders) [4,6,11,12,16,20,22]<br>• Negative views of mental health treatments (caregivers) [9,31]<br>• Concerns about negative impact on caregiver (e.g. increase isolation, negative reactions) (caregivers & stakeholders) [6,13,17,25,31]<br>• Challenging to build therapeutic relationship (stakeholders) [8]<br>• Privacy concerns (caregivers & stakeholders) [3,7,10,17,31,43]<br>• Perceive intervention not appropriate for everyone/cannot be used alone (stakeholders) [1,2]<br>• Perceive information as emotionally challenging or confronting (caregiver) [6,9,12–14,16,20]<br>• Perceive no benefit for caregiver (caregiver) [17]<br>• Perceive as time consuming (stakeholder) [8]<br>• Liability concerns (stakeholder) [3]<br>• Negative view of eHealth and its implementation (stakeholders) [1,2]	• Facilitates sense of connection with other caregivers (caregivers) [4,9,15,17,22,30]<br>• Benefits provider (e.g. learning opportunity) and facilitates caregiver-provider relationship (caregivers & stakeholders) [1,3,7,8,13,21]<br>• Reduce isolation (caregivers) [4,13,15,21,22,25,28,30]<br>• Normalizes and validates lived-experience (caregivers & stakeholders) [4,5,9,13,15,18,20,21,27,28,34]<br>• Benefits caregiver (e.g. improves outlook, well-being, self-care, knowledge, communication, skills, relationships) (caregivers & stakeholders) [1,2,5,6,8,12–23,28,31,32,35,36,38,40,41,44]<br>• Support motivates intervention use (caregivers) [9,13,18,20,31]<br>• Perceive e-mental health as positive (caregivers & stakeholders) [1,31]<br>• Perceive as time efficient (stakeholder) [3,8]<br>• Facilitates support for caregivers in rural settings (stakeholders) [30]<br>• Fills support gap/improves access to support (caregivers & stakeholders) [8,21]	“Some professionals also expressed concerns about the suitability of Internet use for caregivers, since most of them were spouses of patients and likely inexperienced with this technology. Some also thought that computers might increase caregivers’ isolation.” Author summary from [25] – Barrier<br><br>“Caregivers indicated that Pep-Pal was helpful in normalizing many isolating aspects of the caregiving experience, such as the unpredictability of daily caregiving responsibilities. Caregivers also described Pep-Pal as being helpful in providing a sense of social cohesion with other caregivers’ experiences, notably without connecting them to other caregivers.” Author summary from [28] - Facilitator
**Self-Efficacy**	no data	• Building confidence to deliver intervention (stakeholders) [1,3,8]	“Municipality officials reported that successful coaches had confidence in the intervention and their own ability to use it to help their clients.” Author summary from [3] - Facilitator

**Individual Stage of Change**	• Feel care recipient should change (caregivers) [13]<br>• Not providing intervention at the right time (caregivers) [5,9,10,12,13,20,28]<br>• Difficulty accepting help (caregivers) [9,10]<br>• Not emotionally ready (caregivers) [12,13,16]	no data	*"This would have been more useful prior to beginning the current chemo[therapy] regimen and not now at the end of things,"* […] "*Not interested at this time—at the time of initial diagnosis, [I] did spend a lot of time looking up information*." Participant quotes from [10] - Barrier
**Individual Identification with Organization**	no data	• Positive connection with organization staff (caregivers) [4,9,13]	"Participants with a familiar coach reported an intensified relationship after working through the intervention together. Being able to speak freely online and becoming acquainted with the coach on a different level deepened their existing bond." Author summary from [13] - Facilitator
**Other Personal Attributes**	• Perceived or known low digital literacy among caregivers (caregivers & stakeholders) [1,3,4,6,9–11,13,25,26,30]<br>• Low access to internet/computers (caregivers) [9,10,12,13,26]<br>• No interest in type of intervention offered (caregivers) [10,11,20,27,39]<br>• Perceived no need for support/unaware of need (caregivers) [9–12,16,20,27]<br>• Care recipient related challenges to participation (e.g. not wanting care recipient to know they are receiving intervention, don't want to dedicate time to something other than care recipient) (caregivers) [6,9,13,31]<br>• Caregivers are busy/too many other responsibilities (caregivers & stakeholders) [4,5,11,13,16,20–23,26,27,29,30,38]<br>• Support needs met in other ways (e.g. existing social network) (caregivers) [9,16,20,39]<br>• Feelings of shame inhibit sharing experiences (caregivers) [13,20,32]	• Caregivers young age (caregivers & stakeholders) [1,2,4,13,16]<br>• Caregiver being employed (stakeholders) [13]<br>• Caregiver experiencing perceived strain/need for intervention (caregivers) [9,16]<br>• Caregiver receiving inadequate support from social network (caregiver) [9]<br>• Internet skills and familiarity among caregivers (caregivers & stakeholders) [26]<br>• Desire to learn among caregivers (caregivers & stakeholders) [21,30]	"Further conversations with these caregivers revealed that they were too overwhelmed to take on anything new at that time. One had just become a caregiver for an additional relative […]"Author summary from [22] – Barrier<br><br>"*Sometimes you need to tell your story. But my friends were all in a different situation, they just became parents or they were pregnant. A totally different life situation. Therefore, they had problems talking to me. And for my part, I didn't want to be a burden to them either*." Participant quote from [9] - Facilitator

	• Lack of experience with eHealth among stakeholders (stakeholders) [1]		
**Domain 5: Process**			
**Planning**	• Inadequate implementation plans (stakeholders) [1,3]	• Perceived value of planning intervention financing (stakeholders) [2,13]	"The plans that were made at the beginning of the implementation [25] were followed. Nevertheless, these were in many cases insufficient, and in several municipalities, implementation plans are still being made for the future." Author summary from [3] – Barrier<br><br>"importance of […] financing of caregiving support." Author summary from [2] - Facilitator
**Engaging**	• Lack of internal ownership for intervention and implementation (stakeholders) [3]	• Internal ownership for intervention and implementation (stakeholders) [1,3]<br>• Variety of advertising efforts to providers and users (caregivers & stakeholders) [3,17,26]	"The 2 Partner in Balance municipalities that did not consider the implementation to be successful seemed to see the implementation as more of an external project, where the municipality's role was more to facilitate than execute." Author summary from [3] – Barrier<br><br>"What these 3 Partner in Balance municipalities had in common was that they considered the implementation of the intervention to be a success. These municipalities appeared to have a sense of internal responsibility to facilitate the implementation of Partner in Balance and devise creative solutions." Author summary from [3] - Facilitator
**a. Opinion Leaders**	no data	no data	
**b. Formally Appointed Internal Implementation Leaders**	• Lack of engagement from organizational leaders (stakeholders) [3]	no data	"*I introduced this. My supervisor, yes, but I work in my department alone. .... We have not really discussed it with anyone else. So, my supervisor is not actively*

			pushing this now either." Participant quote from [3] - Barrier
**c. Champions**	• Lack of champions/facilitator (stakeholders) [1]	no data	"*It's not like it's ready-made. It's still about people, you have to remember that, you have to facilitate that, you have to motivate that. If you don't do that...everything depends on it, especially in this kind of work. If you think: Yes, now...I have thought it up nicely and it will come naturally...that will not work*." Participant quote from [1] - Barrier
**d. External Change Agents**	no data	• Cooperation with external organizations (stakeholders) [1,3]	"For Partner in Balance, the external cooperation with local health care organizations was an essential part of recruiting the platform's coaches, as they needed to have experience with both dementia and care." Author summary from [1] - Facilitator
**e. Key Stakeholders**	• Lack of practical training (stakeholders) [3]<br>• Low engagement of providers (stakeholders) [1,3]	• High staff familiarity with intervention (stakeholders) [1]<br>• Maintenance of trained staff (stakeholders) [13]<br>• Early engagement in decision-making (stakeholders) [30]	"[Municipalities] would have preferred a more practical, hands-on training in smaller groups, as the training was too theory-focused, and more implementation tips would have been welcome." Author summary from [3] – Barrier<br><br>"One coach stated: *"The program could easily be implemented as regular care if all staff members or a constant group of staff members were trained as coaches*."" Participant quote from [13] - Facilitator

**f. Innovation Participants**	• Recruitment challenges (stakeholders) [1,3,26]<br>• Lack of face-to-face engagement strategies (caregivers) [20]	• Early engagement in decision-making (stakeholders) [1]<br>• Perceived need for strategies to be inclusive and reach diverse groups (e.g. different ages, ethnicities) (caregivers & stakeholders) [1,30,38]<br>• Continuously seeking feedback from users (stakeholders) [26]<br>• Engage entire informal care network (stakeholders) [30]<br>• Face-to-face engagement strategies (stakeholders) [1,30]	"*So finding the coaches of course and maybe...finding the coaches is of course natural, but it is a real challenge...And, of course, reaching sufficient informal caregivers who want to sign up for this.*" Participant quote from [1] – Barrier<br><br>"However, two municipalities assembled a panel of lived-experience experts in dementia and caregiving and chose those activities which the panel identified as most relevant for their community" Author summary from [1] – Facilitator
**Executing**	no data	no data	
**Reflecting & Evaluating**	no data	no data	

Abbreviations: CFIR; Consolidated Framework for Implementation Research

References

1 Christie HL, Schichel MCP, Tange HJ, *et al.* Perspectives from municipality officials on the adoption, dissemination, and implementation of electronic health interventions to support caregivers of people with dementia: Inductive thematic analysis. *JMIR Aging* 2020;**3**:e17255. doi:10.2196/17255

2 Christie HL, Boots LMM, Peetoom K, *et al.* Developing a plan for the sustainable implementation of an electronic health intervention (Partner in Balance) to support caregivers of people with dementia: Case study. *JMIR Aging* 2020;**3**:1–14. doi:10.2196/18624

3 Christie HL, Boots LMM, Tange HJ, *et al.* Implementations of evidence-based ehealth interventions for caregivers of people with dementia in municipality contexts (Myinlife and Partner in Balance): evaluation study. *JMIR Aging* 2021;**4**:e21629. doi:10.2196/21629

4 Kovaleva M, Blevins L, Griffiths PC, *et al.* An online program for caregivers of persons living with dementia: lessons learned. *J Appl Gerontol* 2019;**38**:159–82. doi:10.1177/0733464817705958

5 Pensak NA, Joshi T, Simoneau T, *et al.* Development of a web-based intervention for addressing distress in caregivers of patients receiving stem cell transplants: Formative evaluation with qualitative interviews and focus groups. *JMIR Res Protoc* 2017;**6**. doi:10.2196/resprot.7075

6 Boots LM, de Vugt ME, Withagen HE, *et al.* Development and initial evaluation of the web-based self-management program "Partner in Balance" for family caregivers of people with early stage dementia: an exploratory mixed-methods study. *JMIR Res Protoc* 2016;**5**:e33. doi:10.2196/resprot.5142

7 DuBenske LL, Chih MY, Dinauer S, *et al.* Development and implementation of a clinician reporting system for advanced stage cancer: initial lessons learned. *J Am Med Informatics Assoc* 2008;**15**:679–86. doi:10.1197/jamia.M2532

8 Contreras M, Van Hout E, Farquhar M, *et al.* Therapists' perceptions and acceptability of providing internet-delivered guided self-help acceptance and commitment

therapy (ACT) for family carers of people with dementia (iACT4CARERS): A qualitative study. *Cogn Behav Ther* 2021;**14**:e38. doi:10.1017/S1754470X21000337

9 Köhle N, Drossaert CHC, Oosterik S, *et al.* Needs and preferences of partners of cancer patients regarding a web-based psychological intervention: A qualitative study. *JMIR Cancer* 2015;**1**. doi:10.2196/cancer.4631

10 Buss M, DuBenske L, Dinauer S, *et al.* Patient/Caregiver influences for declining participation in supportive oncology trials. *J Support Oncol* 2008;**6**:168–74.

11 Scott K, Beatty L. Feasibility study of a self-guided cognitive behaviour therapy Internet intervention for cancer carers. *Aust J Prim Health* 2013;**19**:270–4. doi:10.1071/PY13025

12 Cristancho-Lacroix V, Wrobel J, Cantegreil-Kallen I, *et al.* A web-based psychoeducational program for informal caregivers of patients with Alzheimer's disease: A pilot randomized controlled trial. *J Med Internet Res* 2015;**17**:e3717. doi:10.2196/jmir.3717

13 Boots L, de Vugt M, Smeets C, *et al.* Implementation of the blended care self-management program for caregivers of people with early-stage dementia (Partner in Balance): process evaluation of a randomized controlled trial. *J Med Internet Res* 2017;**19**:e423. doi:10.2196/jmir.7666

14 Sikder AT, Yang FC, Schafer R, *et al.* Mentalizing imagery therapy mobile app to enhance the mood of family dementia caregivers: Feasibility and limited efficacy testing. *JMIR Aging* 2019;**2**:1–9. doi:10.2196/12850

15 Contreras M, Van Hout E, Farquhar M, *et al.* Internet-delivered guided self-help Acceptance and Commitment Therapy for family carers of people with dementia (iACT4CARERS): a qualitative study of carer views and acceptability. *Int J Qual Stud Health Well-being* 2022;**17**:2066255. doi:10.1080/17482631.2022.2066255

16 Köhle N, Drossaert CHC, Van Uden-Kraan CF, *et al.* Intent to use a web-based psychological intervention for partners of cancer patients: Associated factors and preferences. *J Psychosoc Oncol* 2018;**36**:203–21. doi:10.1080/07347332.2017.1397831

17 Beer JM, Smith KN, Kennedy T, *et al.* A focus group evaluation of Breathe Easier: a mindfulness-based mHealth app for survivors of lung cancer and their family members. *Am J Heal Promot* 2020;**34**:770–8. doi:10.1177/0890117120924176

18 Bruinsma J, Peetoom K, Bakker C, *et al.* Tailoring and evaluating the web-based 'Partner in Balance' intervention for family caregivers of persons with young-onset dementia. *Internet Interv* 2021;**25**:100390. doi:10.1016/j.invent.2021.100390

19 Zulman DM, Schafenacker A, Barr KLC, *et al.* Adapting an in-person patient-caregiver communication intervention to a tailored web-based format. *Psychooncology* 2012;**21**:336–41. doi:10.1002/pon.1900

20 Köhle N, Drossaert CHC, Jaran J, *et al.* User-experiences with a web-based self-help intervention for partners of cancer patients based on acceptance and commitment therapy and self-compassion: a qualitative study. *BMC Public Health* 2017;**17**:1–16. doi:10.1186/s12889-017-4121-2

21 Bruinsma J, Peetoom K, Boots L, *et al.* Tailoring the web-based 'Partner in Balance' intervention to support spouses of persons with frontotemporal dementia. *Internet Interv* 2021;**26**:100442. doi:10.1016/j.invent.2021.100442

22 Griffiths PC, Whitney MK, Kovaleva M, *et al.* Development and implementation of tele-savvy for dementia caregivers: A department of veterans affairs clinical demonstration project. *Gerontologist* 2016;**56**:145–54. doi:10.1093/geront/gnv123

23 Kubo A, Altschuler A, Kurtovich E, *et al.* A pilot mobile-based mindfulness intervention for cancer patients and their informal caregivers. *Mindfulness (N Y)* 2018;**9**:1885–94. doi:10.1007/s12671-018-0931-2

24 Hales SA, Fossey J. Caring For Me and You: the co-production of a computerised cognitive behavioural therapy (cCBT) package for carers of people with dementia.

Aging Ment Heal 2018;**22**:1287–94. doi:10.1080/13607863.2017.1348475

25 Cristancho-Lacroix V, Moulin F, Wrobel J, *et al.* A web-based program for informal caregivers of persons with alzheimer's disease: An iterative user-centered design. *JMIR Res Protoc* 2014;**3**. doi:10.2196/resprot.3607

26 Baruah U, Shivakumar P, Loganathan S, *et al.* Perspectives on components of an online training and support program for dementia family caregivers in India: a focus group study. *Clin Gerontol* 2020;**43**:518–32. doi:10.1080/07317115.2020.1725703

27 Denzinger A, Bingisser MB, Ehrbar V, *et al.* Web-based counseling for families with parental cancer: Baseline findings and lessons learned. *J Psychosoc Oncol* 2019;**37**:599–615. doi:10.1080/07347332.2019.1602576

28 Carr AL, Jones J, Gilbertson SM, *et al.* Impact of a mobilized stress management program (pep-pal) for caregivers of oncology patients: Mixed-methods study. *JMIR Cancer* 2019;**5**. doi:10.2196/11406

29 Northouse L, Schafenacker A, Barr KLC, *et al.* A tailored Web-based psycho-educational intervention for cancer patients and their family caregivers. *Cancer Nurs* 2014;**37**:321–30. doi:10.1097/NCC.0000000000000159

30 Xiao LD, McKechnie S, Jeffers L, *et al.* Stakeholders' perspectives on adapting the World Health Organization iSupport for dementia in Australia. *Dementia* 2021;**20**:1536–52. doi:10.1177/1471301220954675

31 Bodschwinna D, Lorenz I, Bauereiß N, *et al.* A psycho-oncological online intervention supporting partners of patients with cancer.pdf. *Psychooncology* 2022;**31**:1230–42. doi:10.1002/pon.5917

32 Dragomanovich HM, Dhruva A, Ekman E, *et al.* Being Present 2.0: Online mindfulness-based program for metastatic gastrointestinal cancer patients and caregivers. *Glob Adv Heal Med* 2021;**10**:1–13. doi:10.1177/21649561211044693

33 Price-Blackshear MA, Pratscher SD, Oyler DL, *et al.* Online couples mindfulness-based intervention for young breast cancer survivors and their partners: a randomized-control trial. *J Psychosoc Oncol* 2020;**38**:592–611. doi:10.1080/07347332.2020.1778150

34 Baruah U, Loganathan S, Shivakumar P, *et al.* Adaptation of an online training and support program for caregivers of people with dementia to Indian cultural setting. *Asian J Psychiatr* 2021;**59**:102624. doi:10.1016/j.ajp.2021.102624

35 Demers M, Pagnini F, Phillips D, *et al.* Feasibility of an online Langerian mindfulness program for stroke survivors and caregivers. *OTJR Occup Particip Heal* 2022;**42**:228–37. doi:10.1177/15394492221091266

36 Bingisser MB, Eichelberger D, Ehrbar V, *et al.* Web-based counseling for families with parental cancer: A case report. *Psychooncology* 2018;**27**:1667–9. doi:10.1002/pon.4679

37 Kajiyama B, Thompson LW, Eto-Iwase T, *et al.* Exploring the effectiveness of an Internet-based program for reducing caregiver distress using the iCare Stress Management e-Training Program. *Aging Ment Heal* 2013;**17**:544–54. doi:10.1080/13607863.2013.775641

38 Kajiyama B, Fernandez G, Carter EA, *et al.* Helping Hispanic dementia caregivers cope with stress using technology-based resources. *Clin Gerontol* 2018;**41**:209–16. doi:10.1080/07317115.2017.1377797

39 Pot AM, Blom MM, Willemse BM. Acceptability of a guided self-help Internet intervention for family caregivers: Mastery over dementia. *Int Psychogeriatrics* 2015;**27**:1343–54. doi:10.1017/S1041610215000034

40 Kozlov E, McDarby M, Pagano I, *et al*. The feasibility, acceptability, and preliminary efficacy of an mHealth mindfulness therapy for caregivers of adults with cognitive impairment. *Aging Ment Health* 2021;:1–8. doi:10.1080/13607863.2021.1963949

41 Köhle N, Drossaert CHC, ten Klooster PM, *et al*. Web-based self-help intervention for partners of cancer patients based on acceptance and commitment therapy and self-compassion training : a randomized controlled trial with automated versus personal feedback. *Support Care Cancer* 2021;**29**:5115–25. doi:10.1007/s00520-021-06051-w

42 Henderson C, Knapp M, Fossey J, *et al*. Cost-effectiveness of an online intervention for caregivers of people living with dementia. *J Am Med Dir Assoc* 2022;**S1525-8610**:Advance online publication. doi:10.1016/j.jamda.2022.03.002

43 Dubenske LL, Gustafson DH, Shaw BR, *et al*. Web-based cancer communication and decision making systems: Connecting patients, caregivers, and clinicians for improved health outcomes. *Med Decis Mak* 2010;**30**:732–44. doi:10.1177/0272989X10386382

44 Fauth EB, Novak JR, Levin ME. Outcomes from a pilot online Acceptance and Commitment Therapy program for dementia family caregivers. *Aging Ment Health* 2022;**26**:1620–9. doi:10.1080/13607863.2021.1942432
